# SUMOylation of pseudorabies virus thymidine kinase regulates its nuclear localization and facilitates neuronal replication and reactivation

**DOI:** 10.1371/journal.ppat.1014653

**Published:** 2026-10-05

**Authors:** Xuan Chen, Chuang Li, Dongyue Xing, Jingjing Song, Yun Liu, Yuxin Liu, Qin Huang, Yu Kuang, Xian Li, Jinlei Zhang, Yuqi Zhang, Nianzhi Zhang, Rulan Bai, Changyong Cheng, Jin Yuan, Jun Tang, Rui Zhang

**Affiliations:** 1 State Key Laboratory of Veterinary Public Health and Safety, College of Veterinary Medicine, China Agricultural University, Beijing, China; 2 Department of Basic Veterinary, College of Veterinary Medicine, China Agricultural University, Beijing, China; 3 Department of Preventive Veterinary, College of Veterinary Medicine, China Agricultural University, Beijing, China; 4 Key Laboratory of Applied Biotechnology on Animal Science & Veterinary Medicine of Zhejiang Province, College of Veterinary Medicine of Zhejiang A&F University, Hangzhou, Zhejiang Province, China; 5 Ministry of Education Key Laboratory for Animal Pathogens and Biosafety, College of Veterinary Medicine, Henan Agricultural University, Zhengzhou, Henan, China; University of Southern California, UNITED STATES OF AMERICA

## Abstract

Pseudorabies virus (PRV), an alphaherpesvirus, establishes lifelong latency in neurons and requires efficient reactivation for pathogenesis. Thymidine kinase (TK), encoded by UL23, is a key virulence factor essential for viral replication in non-dividing cells and for reactivation from latency; however, the regulatory mechanisms governing TK function remain poorly defined. Here, we identify TK as a substrate for small ubiquitin-like modifier (SUMO) modification and investigate its functional significance during PRV infection. We show that TK undergoes endogenous SUMOylation during viral infection is conjugated by SUMO-1, SUMO-2, and SUMO-3, with lysine residues K242 and K267 serving as the principal SUMO acceptor sites. Disruption of these SUMOylation sites does not affect TK stability and kinase activity but markedly alters its subcellular localization by reducing nuclear accumulation. Functionally, SUMOylation of TK is dispensable for viral replication in epithelial PK15 cells but facilitates efficient replication in neuronal N2a cells. Using an *in vitro* latency/reactivation model, we further show that SUMOylation-deficient TK does not affect the establishment of latent infection but markedly impairs viral reactivation and subsequent productive replication. In a mouse infection model, disruption of TK SUMOylation attenuates PRV pathogenicity, accompanied by reduced viral loads in the brain and milder histopathological lesions. Collectively, our findings demonstrate that SUMOylation regulates nuclear localization and biological function of PRV TK, thereby facilitating efficient neuronal replication, viral reactivation, and pathogenesis. These findings provide new insights into the post-translational regulation of alphaherpesvirus TK and identify TK SUMOylation as a potential target for controlling PRV infection.

## Author summary

Pseudorabies virus is an important alphaherpesvirus that causes major economic losses in the swine industry and and has recently emerged as an occasional zoonotic pathogen. A hallmark of PRV infection is its ability to establish lifelong latent infection neurons and subsequently reactivate, making viral eradication difficult. In this study, we found that the viral thymidine kinase (TK) protein is modified by a small ubiquitin-like modifier (SUMO) pathway during infection. This modification promotes nuclear accumulation of TK without affecting its kinase activity. Disruption of TK SUMOylation selectively impairs viral replication and reactivation in neuronal cells while having little effect in epithelial cells. Moreover, viruses carrying SUMOylation-deficient TK exhibit markedly reduced pathogenicity in mice. These findings reveal a role of SUMOylation in regulating PRV TK function and demonstrate how a host post-translational modification contributes to efficient neuronal infection and viral pathogenesis. Our study identifies TK SUMOylation as a potential target for developing antiviral strategies against PRV and related alphaherpesviruses.

## Introduction

Pseudorabies virus (PRV), a member of the Alphaherpesvirinae subfamily, is an important neurotropic pathogen that causes significant economic losses in the swine industry and poses potential zoonotic risks [1]. Like other alphaherpesviruses, PRV establishes lifelong latency in the peripheral nervous system and periodically reactivates to produce infectious progeny [2]. Efficient viral replication and reactivation in neurons are therefore central to PRV pathogenesis, yet the molecular mechanisms governing these processes remain incompletely understood.

Thymidine kinase (TK), encoded by the UL23 gene, is a well-characterized virulence factor of alphaherpesviruses. Although dispensable for viral replication in rapidly dividing cells, TK is essential for efficient replication in non-dividing cells such as neurons, where it facilitates nucleotide biosynthesis required for viral DNA replication [3,4]. Consistent with this role, TK is critical for viral reactivation from latency and neurovirulence *in vivo* [5–9]. However, despite its importance, the regulatory mechanisms that control TK function, particularly in the context of neuronal infection and latency–reactivation cycles, remain poorly defined.

Post-translational modifications (PTMs) provide a rapid and reversible means to regulate protein function, localization, and interaction networks. Among these, SUMOylation—the covalent attachment of small ubiquitin-like modifier (SUMO) proteins to lysine residues—has emerged as a key regulator of nuclear processes, including gene expression, DNA repair, and protein trafficking [10]. Increasing evidence indicates that viruses extensively exploit the host SUMOylation machinery to facilitate infection [11]. Viral proteins from diverse families can be SUMOylated, and this modification can regulate viral replication, assembly, and immune evasion [12–15]. In herpesviruses, SUMOylation has been implicated in both latent and lytic phases of infection, where it shapes viral gene expression and host chromatin dynamics [16]. For example, the immediate-early protein ICP0 of herpes simplex virus 1 (HSV-1) functions as a SUMO-targeted ubiquitin ligase (STUbL) that promotes the degradation of SUMOylated host restriction factors, thereby enhancing lytic infection [17]. The latency-associated nuclear antigen (LANA) of Kaposi’s sarcoma-associated herpesvirus (KSHV) is SUMOylated and recruits SUMOylated chromatin modifiers to maintain viral episomes during latency [18]. In Epstein–Barr virus (EBV), SUMOylation of the transcription factor Zta (BZLF1) regulates its transactivation activity and contributes to the switch from latency to lytic replication [19,20].

Despite these advances, whether PRV is subject to SUMOylation and how this modification contributes to its function during infection remain unknown. In this study, we identify PRV TK as a SUMO-modified protein and define lysine residues K242 and K267 as its principal SUMO acceptor sites. We demonstrate that SUMOylation promotes nuclear localization of TK through a dynamic and reversible mechanism involving SUMO-specific proteases. Functionally, we show that TK SUMOylation is dispensable for viral replication in epithelial cells but is required for efficient replication and reactivation in neuronal cells. Moreover, disruption of TK SUMOylation markedly attenuates PRV pathogenicity *in vivo*. Together, our findings uncover a role for SUMOylation in regulating TK function and provide mechanistic insight into how SUMO-dependent trafficking of a viral metabolic enzyme contributes to herpesvirus neurotropism and virulence.

## Results

### PRV TK protein is modified by SUMO

To identify SUMOylated PRV proteins in mammalian cells, we performed a candidate-based SUMOylation screen. The viral proteins (including Us1, Us3, UL13, TK, and UL48), which possess well-established enzymatic or regulatory functions during PRV infection, were selected for analysis.. Flag-tagged plasmids encoding individual viral proteins or the positive control PML4 were co-transfected into HEK293 cells together with HA-tagged SUMO-1 and the SUMO-conjugating enzyme Ubc9. Following rapid lysis in RIPA buffer supplemented with N-ethylmaleimide (NEM), Flag-tagged proteins were immunoprecipitated using an anti-Flag antibody and analyzed by immunoblotting with an anti-HA antibody. As shown in Fig 1A, Flag-TK exhibited additional higher-molecular-weight bands that were recognized by the anti-HA antibody, indicating covalent modification by HA-SUMO-1. In contrast, no comparable SUMOylated species were observed for the other PRV proteins examined. As expected, the positive control PML4 displayed robust SUMOylation under the same conditions.

**Fig 1:**
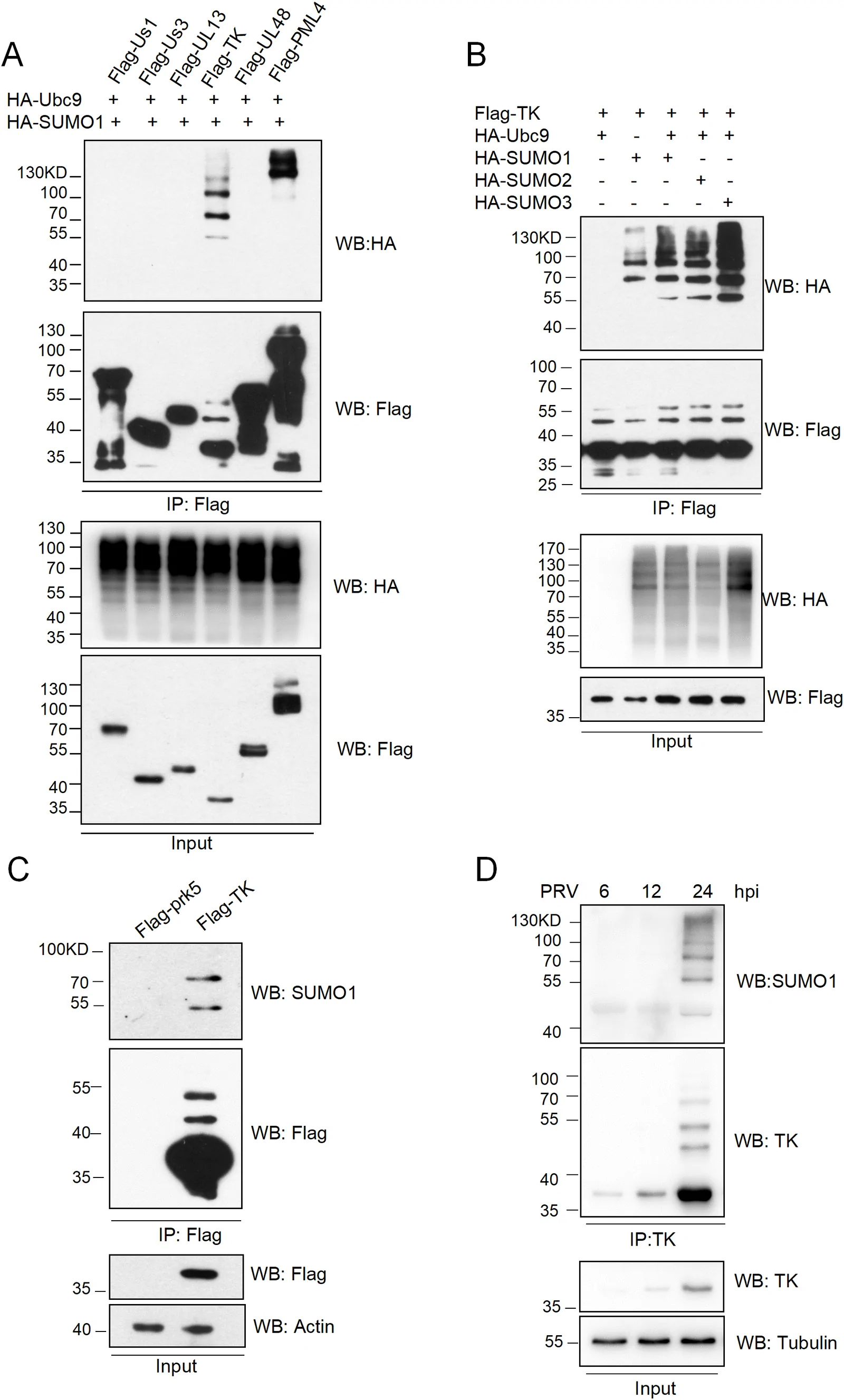
PRV TK protein is modified by SUMO. **(A)** HEK293 cells were co-transfected with plasmids expressing Flag-tagged PRV proteins (US1, US3, UL13, TK, or UL48) or Flag-PML4 (positive control), together with HA-Ubc9 and HA-SUMO1. At 24 h post-transfection, whole-cell lysates and Flag immunoprecipitates were analyzed by immunoblotting using anti-HA and anti-Flag antibodies. **(B)** HEK293 cells were co-transfected with Flag-TK together with HA-Ubc9 and HA-SUMO1, HA-SUMO2, or HA-SUMO3. At 24 h post-transfection, whole-cell lysates and Flag immunoprecipitates were analyzed by immunoblotting using anti-HA and anti-Flag antibodies. **(C)** HEK293 cells were transfected with Flag-TK or the empty vector (Flag-prk5). At 24 h post-transfection, whole-cell lysates and Flag immunoprecipitates were analyzed by immunoblotting using anti-SUMO1 and anti-Flag antibodies to detect endogenous SUMO1 conjugation. **(D)** PK15 cells were infected with PRV (MOI = 0.1) for the indicated times. Cell lysates were subjected to denaturing immunoprecipitation using an anti-TK antibody, followed by immunoblotting with anti-SUMO1 and anti-TK antibodies to detect endogenous SUMOylation of TK during PRV infection.

In addition to SUMO-1, the closely related paralogs SUMO-2 and SUMO-3 are ubiquitously expressed and conjugated to target proteins through a similar mechanism. We therefore examined whether TK could also be modified by these paralogs. The SUMOylation assays demonstrated that TK can be conjugated by HA-SUMO-1, HA-SUMO-2, and HA-SUMO-3, as evidenced by the appearance of multiple slower-migrating bands (Fig 1B), indicating that TK is a substrate for all three SUMO isoforms.

To determine whether TK undergoes SUMOylation by endogenous SUMO, Flag-TK was immunoprecipitated and analyzed using an anti-SUMO-1 antibody. As shown in Fig 1C, multiple higher-molecular-weight SUMO-1-reactive bands were detected in the Flag-TK immunoprecipitates but not in the Flag-prk5 control, confirming that TK is conjugated by endogenous SUMO-1. Finally, we examined whether TK is SUMOylated during PRV infection. Immunoprecipitation followed by immunoblot analysis revealed the presence of SUMOylated TK species at 24 h post-infection (hpi) (Fig 1D), indicating that TK undergoes SUMO modification during viral infection.

### Lysine 242 and lysine 267 are the major SUMO acceptor sites in TK

We next sought to identify the lysine residue(s) in TK responsible for SUMO conjugation. Because SUMO-1 is covalently attached to lysine residues, individual (K16R, K56R, K242R, or K267R), double, and quadruple (4KR) lysine-to-arginine mutants were generated (Fig 2A) and assessed for SUMOylation. As shown in Fig 2B, mutation of either K242 or K267 markedly reduced SUMO-1 conjugation of TK, whereas substitution at K16 or K56 had little effect (Fig 2B). Notably, simultaneous mutation of K242 and K267, as well as the 4KR mutant, completely abolished SUMOylation of full-length TK (Fig 2C), indicating that K242 and K267 serve as the primary SUMO-1 acceptor sites. To further validate these findings, SUMOylated TK was examined using an anti-SUMO-1 antibody. Consistent with the results obtained using HA-SUMO-1, endogenous SUMO-1 conjugation was readily detected in wild-type TK (Flag-TK-WT) but was completely absent in the Flag-K242/K267R mutant (Fig 2D), further confirming that lysine 242 and lysine 267 are the critical acceptor sites for SUMO-1 modification of TK.

**Fig 2:**
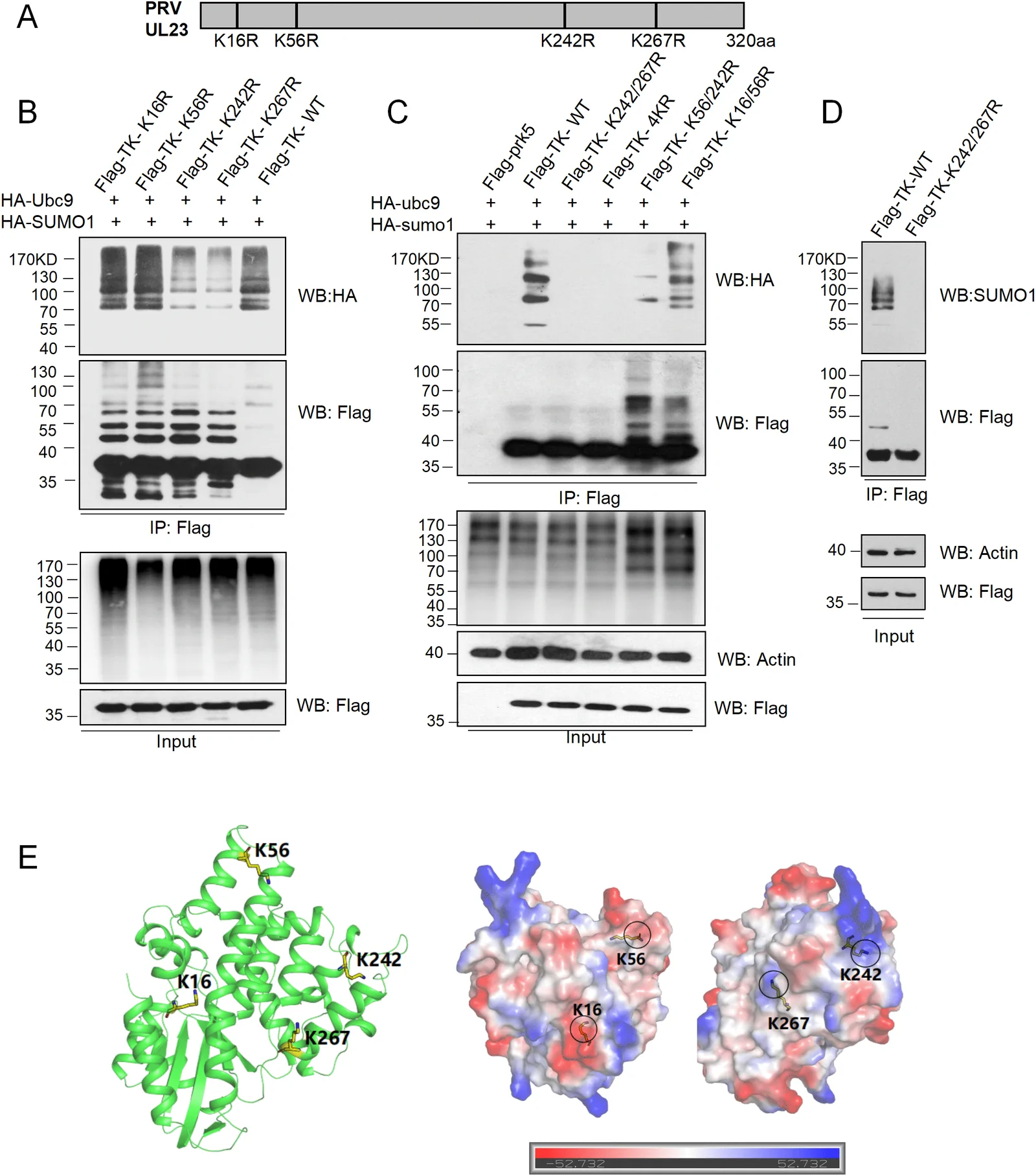
Lysine 242 and lysine 267 are the major SUMOylation sites of PRV TK. **(A)** Schematic representation of the predicted SUMOylation site mutants of PRV TK. **(B, C)** Identification of SUMOylation sites in PRV TK. HEK293T cells were co-transfected with plasmids expressing Flag-tagged wild-type TK (WT) or the indicated lysine-to-arginine (K-to-R) mutants together with HA-Ubc9 and HA-SUMO1. At 24 h post-transfection, whole-cell lysates and Flag immunoprecipitates were analyzed by immunoblotting using anti-HA and anti-Flag antibodies. The 4KR mutant contains substitutions at all four predicted lysine residues. **(D)** HEK293 cells were transfected with Flag-TK-WT or Flag-TK-K242/267R for 24 **h.** Whole-cell lysates and Flag immunoprecipitates were analyzed by immunoblotting using anti-SUMO1 and anti-Flag antibodies to detect endogenous SUMO1 conjugation. **(E)** Structural model of PRV TK predicted by AlphaFold (Model ID: AF-G3G8S8-F1). Ribbon representation (left) and electrostatic surface potential (right) are shown. The four candidate SUMOylation lysine residues are indicated.

SUMO-1 displays a predominantly negatively charged surface, whereas lysine residues can contribute positively charged regions that facilitate SUMO interaction. To further examine the structural basis of TK SUMOylation, we analyzed the surface charge distribution of TK using PyMOL. As shown in Fig 2E, K242 and K267 are located within positively charged α-helical regions, whereas K16 and K56 reside in negatively charged surface areas. This charge complementarity suggests that K242 and K267 may preferentially mediate electrostatic interactions with SUMO-1. Together, these observations support a model in which TK interacts with and is modified by SUMO-1 primarily through K242 and K267.

### Mutation of the SUMOylation sites in TK does not affect its stability and kinase activity

SUMOylation is known to modulate protein stability and functional activity. To assess whether SUMOylation influences the stability of TK, HeLa cells expressing Flag-TK-WT or K242R/K267R mutant were treated with cycloheximide (CHX) for the indicated times, and protein levels were analyzed by immunoblotting. As shown in Fig 3A, TK-WT and the K242R/K267R mutant exhibited comparable degradation kinetics following CHX treatment, indicating that disruption of SUMOylation does not alter TK stability. Because SUMOylation can antagonize or cooperate with ubiquitination, we next examined whether mutation of the SUMOylation sites influences ubiquitination of TK. HEK293 cells were co-transfected with TK-WT, K242/267R, or 4KR mutants together with a ubiquitin (Ub) expression plasmid, followed by treatment with the proteasome inhibitor MG132. Western blot analysis revealed comparable levels of ubiquitination between TK-WT and the SUMO-deficient mutants (Fig 3B), indicating that SUMOylation does not influence the ubiquitination of TK. Finally, we investigated whether SUMOylation influences the intrinsic kinase activity of TK. Flag-TK-WT, Flag-TK-K242R/K267R, and Flag-TK-4KR proteins were immunoprecipitated using anti-Flag beads and subjected to *in vitro* kinase assays using the ADP-Glo Kinase Assay system. As shown in Fig 3C, no significant differences in kinase activity were observed among the three proteins. These results demonstrate that disruption of SUMOylation does not impair the intrinsic catalytic activity of TK.

**Fig 3:**
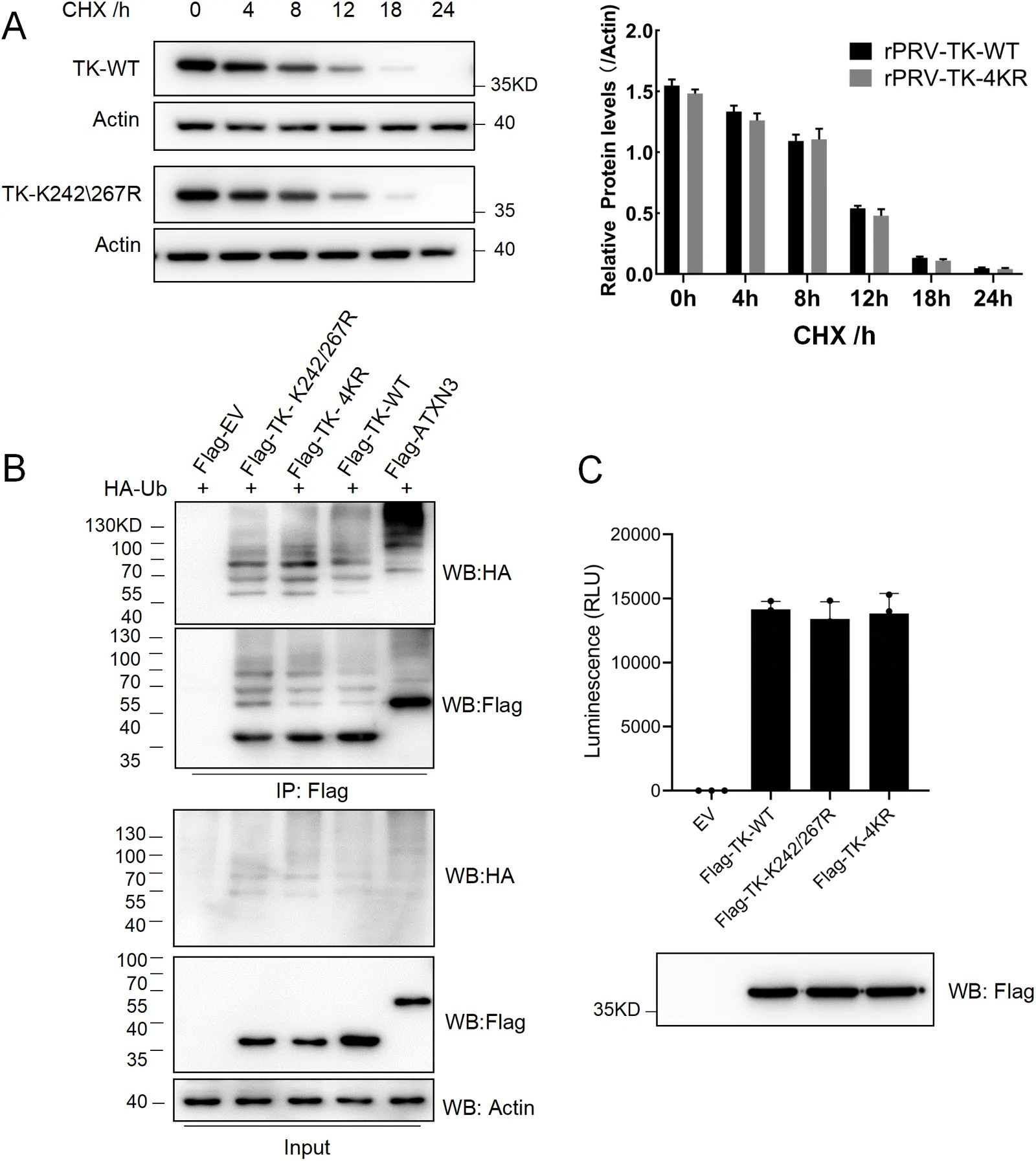
Mutation of TK SUMOylation sites does not alter TK stability or kinase activity. **(A)** HeLa cells were transfected with plasmids expressing Flag-TK-WT or Flag-TK-K242/267R. At 24 h post-transfection, cells were treated with cycloheximide (CHX; 50 μg/mL) for the indicated times. Whole-cell lysates were analyzed by immunoblotting to evaluate TK protein stability. **(B)** HEK293T cells were co-transfected with plasmids expressing Flag-TK-WT, Flag-TK-K242/267R, or Flag-TK-4KR together with HA-Ub. Cells were treated with MG132 (20 μM) for 4 h before harvest. Whole-cell lysates and Flag immunoprecipitates were analyzed by immunoblotting using anti-HA and anti-Flag antibodies. Flag-ATXN3 and the empty vector (Flag-prk5) were included as positive and negative controls, respectively. **(C)** Kinase activities of purified Flag-TK-WT, Flag-TK-K242/267R, and Flag-TK-4KR proteins were measured using the ADP-Glo Kinase Assay. Purified proteins were incubated with 100 μM thymidine and 100 μM ATP for 40 min, and kinase activity was quantified as relative luminescence units (RLU). Data are presented as mean ± SD from three independent experiments. The purified Flag-TK proteins used in the kinase assay were verified by immunoblotting and are shown below.

Collectively, these findings indicate that SUMOylation of TK is dispensable for its stability, ubiquitination, and kinase activity.

### SUMOylation contributes to efficient nuclear localization of TK

Having established that disruption of SUMOylation does not affect the stability, ubiquitination, or kinase activity of TK, we next investigated whether SUMOylation regulates its subcellular localization. Immunofluorescence analysis showed that TK-WT and the K16/56R mutant displayed diffuse nuclear localization. In contrast, mutants carrying substitutions at one or both SUMOylation sites (K242R, K267R, or K242R/K267R) showed markedly reduced nuclear localization and accumulated as punctate structures in the cytoplasm (Fig 4A). To corroborate these observations, nuclear and cytoplasmic fractions were prepared and analyzed by immunoblotting following immunoprecipitation. As shown in Fig 4B, TK-WT was predominantly localized in the nucleus (Fig 4B, input lanes 3 and 4), whereas mutation of K242 and K267 significantly increased its cytoplasmic distribution (Fig 4B, input lanes 5 and 6). Consistently, SUMO-1 conjugated TK was detected exclusively in the nuclear fraction of TK-WT but not in the TK-K242\267R mutant (Fig 4B, IP lanes 4 and 6). Collectively, these results indicate that SUMOylation of TK is required for its efficient nuclear accumulation.

**Fig 4:**
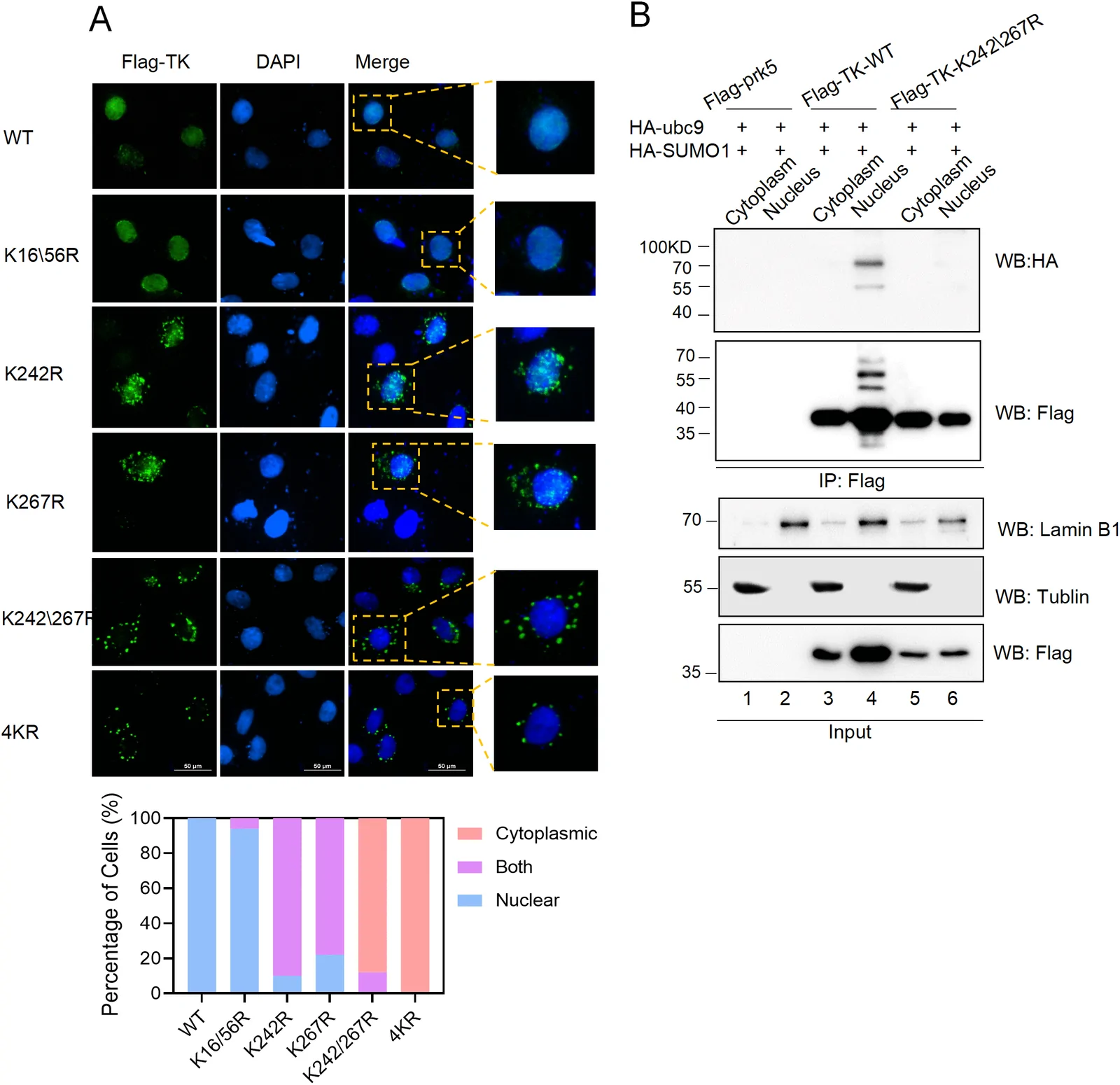
SUMOylation contributes to efficient nuclear localization of TK. **(A)** HeLa cells were transfected with plasmids expressing Flag-TK-WT or the indicated lysine-to-arginine (K-to-R) mutants. At 24 h post-transfection, cells were analyzed by confocal immunofluorescence microscopy using an anti-Flag antibody (green), and nuclei were counterstained with DAPI (blue). Subcellular localization was quantified by scoring the percentages of cells exhibiting nuclear, cytoplasmic, or both nuclear and cytoplasmic localization. At least 50 cells from three independent experiments were analyzed. **(B)** HeLa cells were transfected with plasmids expressing Flag-TK-WT, Flag-TK-K242/267R, or Flag-TK-4KR. At 24 h post-transfection, nuclear and cytoplasmic fractions were prepared and analyzed by immunoblotting using anti-Flag antibody. Tubulin and Lamin B1 were used as cytoplasmic and nuclear fraction markers, respectively.

### Desumoylation of TK by SUMO-special proteases (SENPs) reduces its nucleus localization during PRV infection

Sumoylation is a highly dynamic and reversible post-translational modification regulated by SUMO-specific proteases (SENPs), which catalyze the removal of SUMO from substrate proteins. To determine whether SENPs regulate TK SUMOylation and subcellular localization, SENP1, SENP2, and SENP3, as well as their catalytically inactive mutants (SENP1m, SENP2m, and SENP3m), were individually fused to GFP and co-expressed with HA-SUMO-1 and Flag-TK. As shown in Fig 5A, co-expression of SENP1 or SENP2 markedly reduced the SUMOylated forms of TK, whereas SENP3 or the catalytically inactive mutants (SENP1m, SENP2m, and SENP3m) had no apparent effect, with SUMOylated TK species remaining comparable to those observed in cells transfected with the empty vector (EV). These results indicate that SENP1 and SENP2, but not SENP3, efficiently mediate the deconjugation of SUMO-1 from TK.

**Fig 5:**
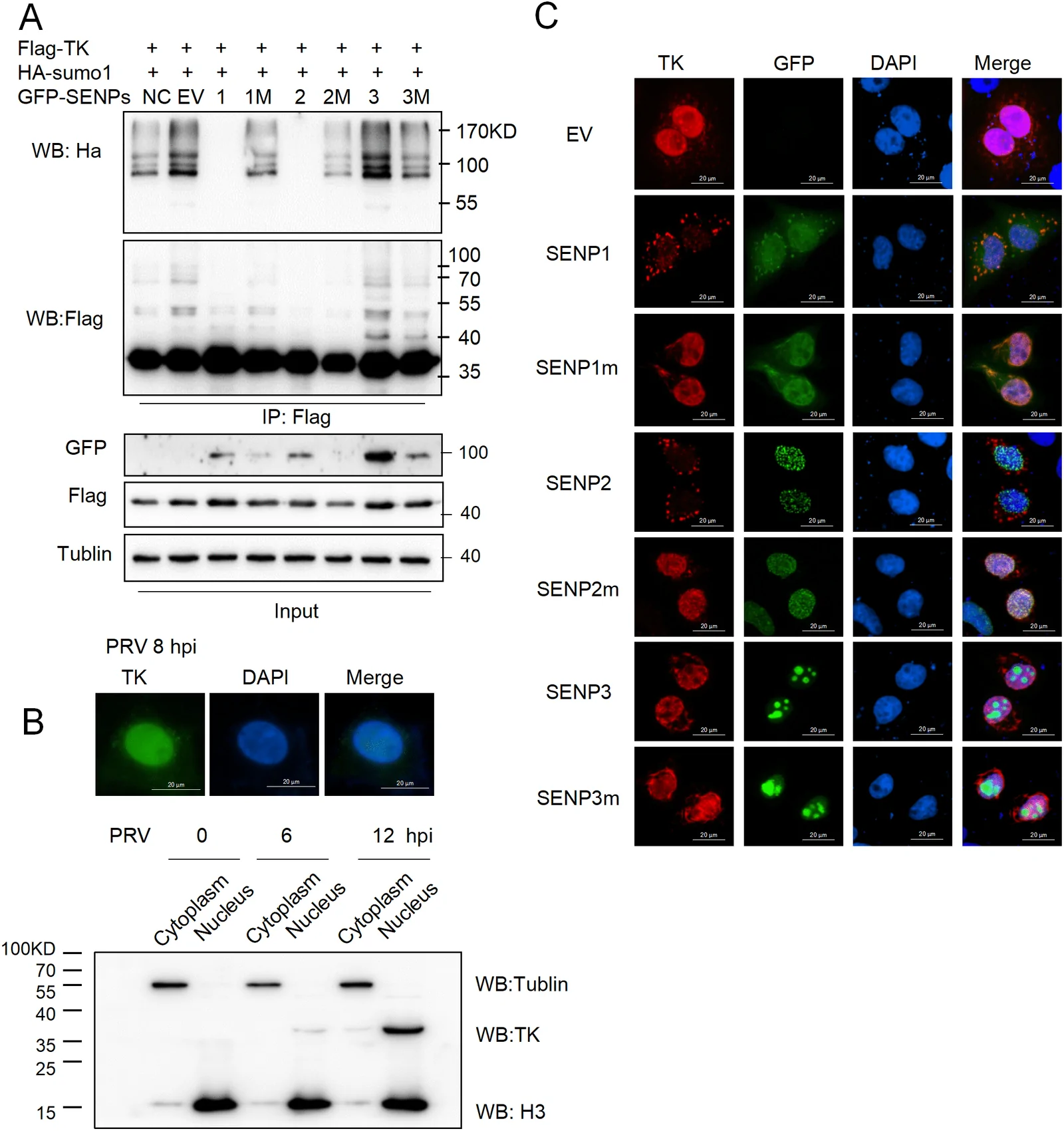
DeSUMOylation by SUMO-specific proteases (SENPs) reduces the nuclear localization of PRV TK during PRV infection. **(A)** HEK293 cells were co-transfected with Flag-TK, HA-SUMO1, and the indicated GFP-tagged SENP expression plasmids. At 24 h post-transfection, whole-cell lysates and Flag immunoprecipitates were analyzed by immunoblotting using anti-HA and anti-Flag antibodies. **(B)** PK15 cells were infected with PRV (MOI = 1) for the indicated times. TK expression was analyzed by immunofluorescence microscopy (upper panels) and immunoblotting (lower panels). **(C)** PK15 cells were transfected with the indicated GFP-SENP plasmids and infected with PRV (MOI = 1). At 24 h post-infection, cells were analyzed by confocal immunofluorescence microscopy using anti-TK (red) and anti-GFP (green) antibodies. Nuclei were counterstained with DAPI (blue).

To determine whether TK is regulated by SUMOylation during PRV infection by exploiting SENP-mediated deSUMOylation, TK **localization was examined at different time points post-infection (MOI** = **1) in** PK**15 cells by immunofluorescence staining and cellular fractionation. The results showed that** TK **predominantly accumulated in the nucleus throughout the course of infection (**Fig 5B**). We next assessed the effect of** SENP**s on** TK **subcellular localization during** PRV **infection using immunofluorescence analysis (**Fig 5C**). In cells overexpressing** SENP**1 or** SENP**2,** TK **was largely redistributed to the cytoplasm, where it appeared as punctate aggregates. In contrast, in** SENP**3-overexpressing cells, only a minor fraction of** TK **was detected in the cytoplasm, with the majority remaining in the nucleus. In cells expressing the catalytically inactive** SENP **mutants,** TK **exhibited a diffuse nuclear distribution similar to that observed under control conditions (**Fig 5C**). Collectively, these findings demonstrate that** SENP**1- and** SENP**2-mediated de**SUMO**ylation reduces the nuclear accumulation of** TK **during** PRV **infection, supporting a role for** SUMO**ylation in promoting its nuclear localization.**

### SUMOylation-deficient TK impairs PRV replication in neuronal but not epithelial cells

To define the functional relevance of TK SUMOylation during PRV infection, we generated recombinant viruses carrying SUMOylation-deficient mutations. Specifically, rPRV-TK-4KR, harboring lysine-to-arginine substitutions at K16, K56, K242, and K267, and its wild-type counterpart (rPRV-TK-WT) were engineered by inserting the respective TK expression cassettes into the viral genome of TK-null virus (PRV-TK-KO) at the Us3 locus using CRISPR/Cas9 (Fig 6A). Viral rescue and expression of TK, Us3, VP5, and gB were confirmed by next-generation sequencing (S1 Fig), PCR and western blotting (Fig 6B), with PRV-WT and PRV-TK-KO included as controls.

**Fig 6:**
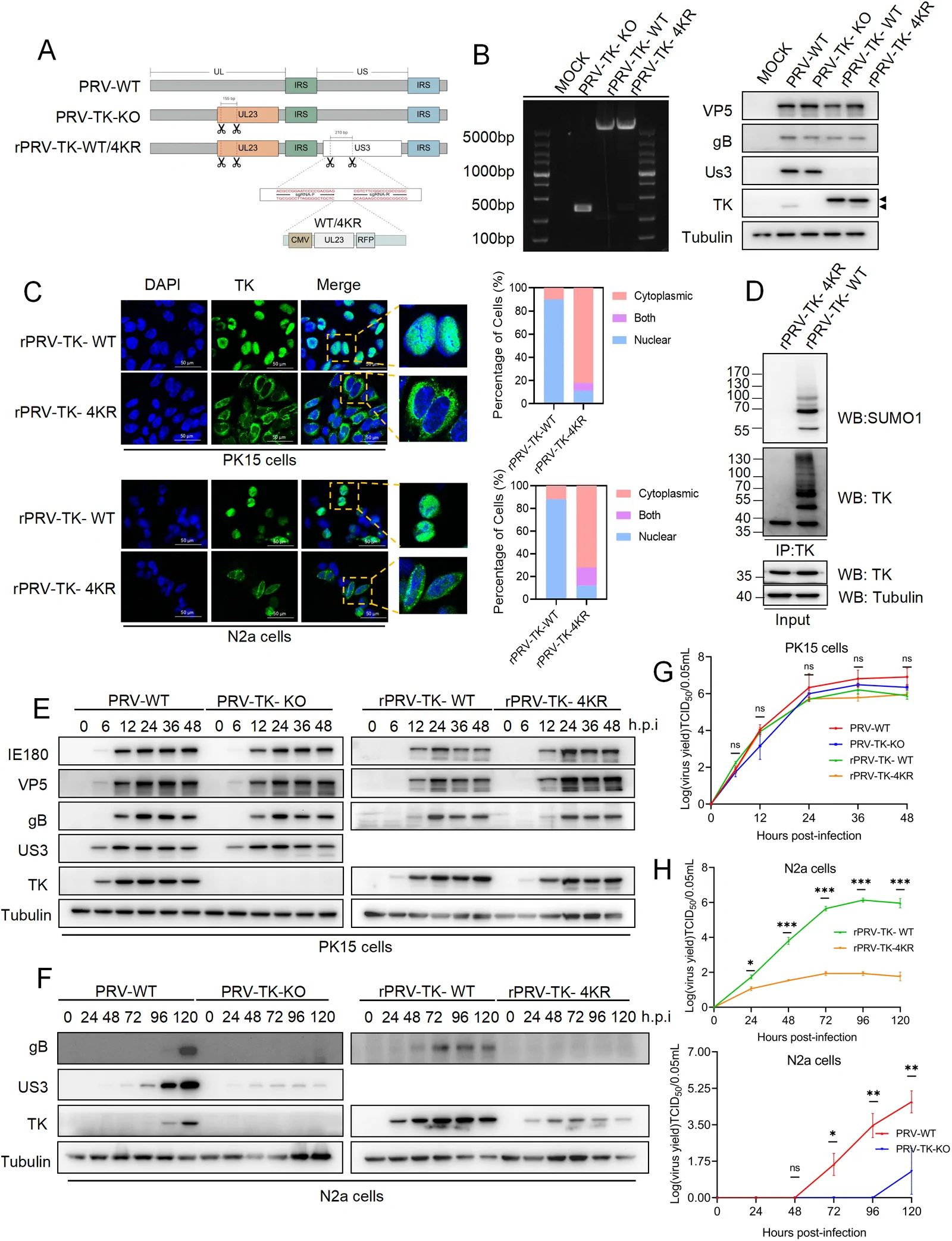
SUMOylation-deficient mutation of TK impairs PRV replication in N2a cells but not in PK15 cells. **(A)** Schematic illustration of the recombinant viruses rPRV-TK-WT and the SUMOylation-deficient mutant rPRV-TK-4KR. The recombinant viruses were engineered by inserting the respective TK expression cassettes into the viral genome of TK-null virus (PRV-TK-KO) at the Us3 locus using CRISPR/Cas9 **(B)** Characterization of recombinant viruses. PK15 cells infected with the indicated viruses were analyzed at 24 h post-infection by PCR (left) and immunoblotting using antibodies against the indicated PRV proteins (right). **(C)** PK15 and N2a cells were infected with rPRV-TK-WT or rPRV-TK-4KR (MOI = 1) for 24 h and analyzed by confocal immunofluorescence microscopy using an anti-TK antibody. Nuclei were counterstained with DAPI. Subcellular localization was quantified by scoring the percentages of cells exhibiting nuclear, cytoplasmic, or both nuclear and cytoplasmic localization. At least 50 cells from three independent experiments were analyzed. **(D)** PK15 cells were infected with PRV or the indicated recombinant viruses (MOI = 0.1) for 24 **h.** Cell lysates were subjected to denaturing immunoprecipitation using an anti-TK antibody, followed by immunoblotting with anti-SUMO1 and anti-TK antibodies to detect endogenous SUMOylation of TK during infection. **(E–H)** PK15 and N2a cells were infected with the indicated viruses at the indicated time points. Viral protein expression was analyzed by immunoblotting **(E, F)**, and culture supernatants were collected for determination of infectious virus titers by TCID_50_ assay. Statistical significance was determined using one-way ANOVA followed by Tukey’s multiple-comparison test. *P < 0.05, **P < 0.01, ***P < 0.001.

Immunofluorescence analysis revealed that, in both PK15 and N2a cells, TK derived from rPRV-TK-4KR was predominantly redistributed to cytoplasmic puncta, whereas TK from PRV-WT and rPRV-TK-WT exhibited robust nuclear accumulation (Fig 6C), indicating that SUMOylation is required for proper nuclear targeting of TK during infection.To directly determine whether TK is SUMOylated in the context of recombinant viral infection, we performed SUMOylation analysis using PK15 cells infected with either rPRV-TK-WT or rPRV-TK-4KR. Consistent with the results obtained from ectopic expression assays, TK from rPRV-TK-WT-infected cells showed detectable endogenous SUMO modification, whereas no apparent SUMOylated TK signal was observed in cells infected with rPRV-TK-4KR (Fig 6D), confirming that the introduced lysine mutations effectively abolished TK SUMOylation during authentic PRV infection. We next assessed the impact of TK SUMOylation on viral replication in PK15 and N2a cells. In PK15 cells, no measurable differences were observed among PRV-WT, PRV-TK-KO, rPRV-TK-4KR, and rPRV-TK-WT in viral protein expression (Fig 6E) and infectious titers (Fig 6G) over 0–48 h post-infection. In contrast, in N2a cells, the SUMOylation-deficient mutant rPRV-TK-4KR exhibited a pronounced replication defect relative to its wild-type counterpart rPRV-TK-WT (Fig 6F and 6H). Although PRV-WT was capable of replicating in N2a cells, replication was markedly delayed, with detectable viral protein expression (Fig 6F) and progeny production (Fig 6H) only at 72 h post-infection, compared to 24 h for rPRV-TK-WT. This enhanced replication of rPRV-TK-WT is likely attributable to CMV-driven overexpression of TK, which may potentiate viral replication in neuronal cells. Consistent with prior studies, PRV-TK-KO failed to replicate in N2a cells.

Together, these data indicate that TK SUMOylation is dispensable for PRV replication in rapidly dividing epithelial cells (such as PK15) but is required for efficient replication in neuronal cells (such as N2a). These findings support a model in which SUMOylation-dependent nuclear localization of TK contributes to PRV adaptation to the neuronal environment.

### SUMOylation of TK is required for efficient PRV reactivation from latency in neuronal cells

Previous studies have shown that TK is dispensable for the establishment of PRV latency but is critical for efficient viral reactivation in neurons [8,9]. To determine whether TK SUMOylation contributes to this process, we established an *in vitro* latency-like and reactivation model in N2a cells. In brief, latency was induced by culturing infected cells at 42 °C for 3 days, followed by a shift to 37 °C for 5 days to trigger reactivation. The model was validated by qPCR, TCID_50_ assays, and western blotting. Infection with PRV-WT or PRV-TK-KO (MOI = 1) under 42 °C conditions resulted in a marked reduction in viral genome copies (gB), as well as the transcription levels of immediate early gene IE180 and Latency-Associated Transcript (LAT) of PRV, compared to 37 °C controls (S2A–S2C Fig). Consistently, no detectable viral genome amplification, infectious particles, or viral protein expression was observed under these conditions (S2D–S2F Fig), indicating successful establishment of latency-like infection.

Upon temperature shift to 37 °C, PRV-WT-infected cells exhibited progressive increases in viral genome copies, IE180 and LAT expression, and infectious virus production beginning at 1 day post-reactivation. In contrast, PRV-TK-KO-infected cells remained at baseline levels across all assays, with no detectable viral genome replication, protein expression, or progeny virus production (S3A–S3F Fig). These data confirm that TK is required for efficient reactivation in this system.

To assess the role of TK SUMOylation, N2a cells were infected with PRV-WT, PRV-TK-KO, PRV-US3-KO, PRV-TK/US3-KO, rPRV-TK-WT, or rPRV-TK-4KR (MOI = 1), subjected to latency induction (42 °C, 3 days), and then shifted to 37 °C (5 days) for reactivation. Notably, PRV-US3 KO and PRV-TK/US3 KO were included as control viruses to exclude potential effects arising from disruption of the Us3 locus in the recombinant viruses. The results showed that after 3 days of incubation at 42 °C, all viruses exhibited similarly low levels of extracellular viral genome copies (Fig 7A, left panel), IE180 and LAT transcripts (Fig 7B, upper panel), intracellular viral DNA (Fig 7C, left panel), viral protein expression (Fig 7D, left panel), and infectious virus production (Fig 7E, left panel), with no significant differences from the mock control but marked differences from the lytic infection control maintained at 37 °C. These results indicate that all viruses, including rPRV-TK-WT and rPRV-TK-4KR, established comparable latent-like infections before reactivation.

**Fig 7:**
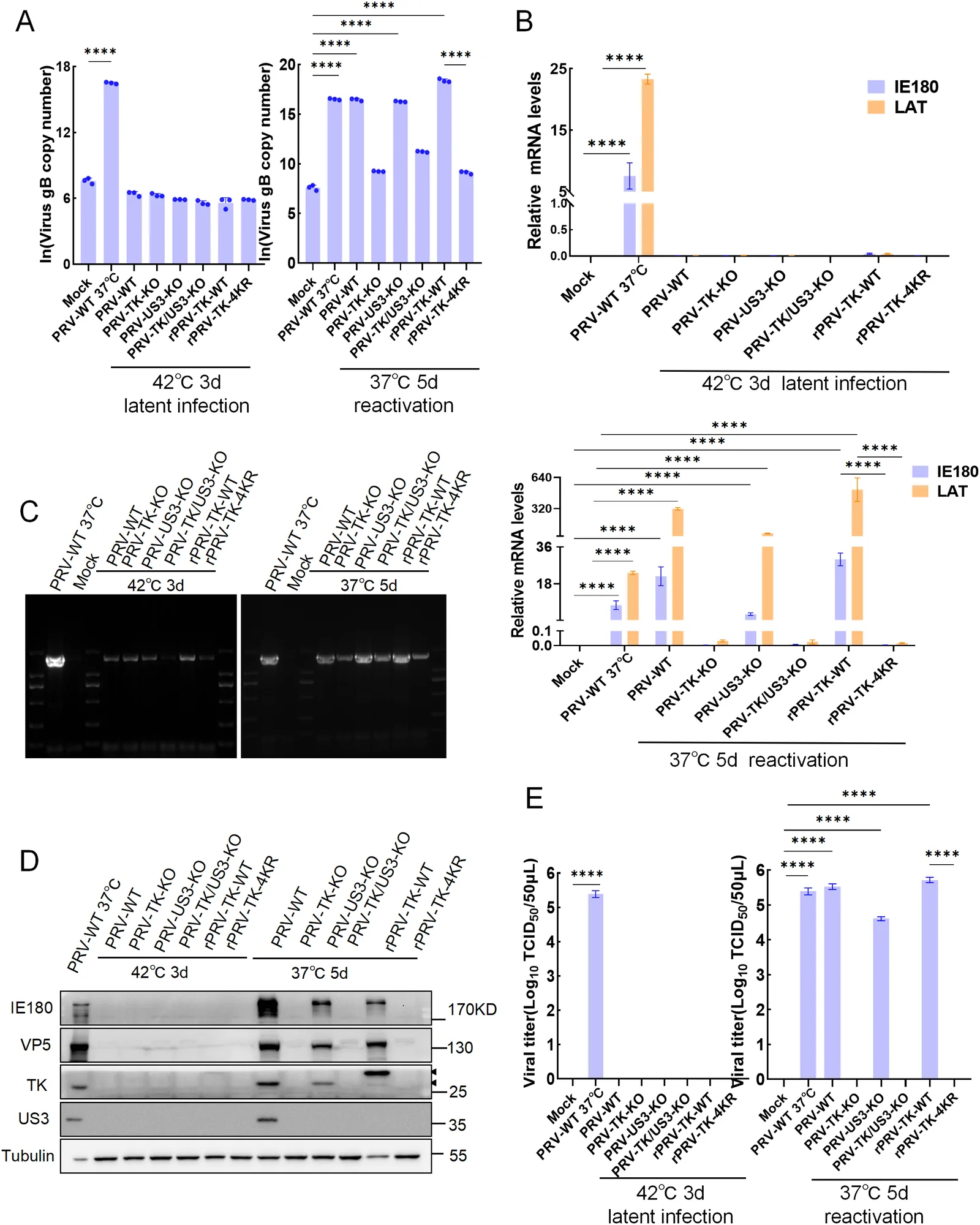
SUMOylation of TK is required for efficient PRV reactivation from latency in N2a cells. N2a cells were infected with the indicated viruses (MOI = 1) and cultured at either 37 °C or 42 °C with medium replacement every 24 **h.** A latency-like state was established by incubation at 42 °C for 3 days, followed by a temperature shift to 37 °C for 5 days to induce viral reactivation. Cells and culture supernatants were collected after latency induction (42 °C, 3 days) or following reactivation (37 °C, 5 days). **(A)** Extracellular PRV gB DNA copy numbers in culture supernatants were determined by qPCR. **(B)** IE180 and LAT transcript levels in infected cells were determined by RT-qPCR. **(C)** Intracellular PRV DNA copy numbers in infected cells were determined by qPCR. **(D)** Viral protein expression in cell lysates was analyzed by immunoblotting. **(E)** Infectious virus titers in culture supernatants were determined by TCID50 assay on PK15 cells. Quantitative data are presented as mean ± SD from three independent experiments. Statistical significance was determined using one-way ANOVA followed by Tukey’s multiple-comparison test. ****P < 0.0001.

Following temperature shift to 37 °C, qPCR analysis showed that viral genome copies in the supernatant recovered to high levels in PRV-WT, PRV-US3-KO, and rPRV-TK-WT groups, comparable to the lytic control, whereas PRV-TK-KO, PRV-TK/US3-KO, and rPRV-TK-4KR remained at baseline levels (Fig 7A, right panel). Consistently, IE180 and LAT transcription (Fig 7B, down panel), intracellular viral genome levels (Fig 7C, right panel), viral protein expression (Fig 7D, right panel), and infectious titers (Fig 7E, right panel) were robustly restored in rPRV-TK-WT but remained markedly impaired in rPRV-TK-4KR. Similar defects were observed in the PRV-TK-KO and PRV-TK/US3-KO groups, which also failed to recover viral replication after the temperature shift.

Collectively, these findings demonstrate that disruption of TK SUMOylation does not impair establishment of the latent-like state but specifically compromises efficient viral reactivation and productive replication following latency induction in neuronal cells.

### TK SUMOylation deficiency attenuates PRV pathogenicity *in vivo*

In addition to its role in viral replication, TK is a key determinant of PRV virulence. To determine the contribution of TK SUMOylation to PRV pathogenicity *in vivo*, five-week-old female BALB/c mice were intranasally inoculated with 5 × 10^5^ TCID_50_ of PRV-WT, PRV-US3-KO, PRV-TK/US3-KO, rPRV-TK-WT, rPRV-TK-4KR, or DMEM as a mock control, and monitored for 14 days.

Mice infected with PRV-WT developed severe pruritus and scratching behavior by 4 days post-infection (dpi), and all animals succumbed to infection within 6 dpi (Fig 8A). Consistent with the established role of Us3 in PRV neurovirulence, PRV-US3-KO-infected mice exhibited similar clinical signs but showed delayed disease progression, with 100% mortality occurring by 7 dpi. In comparison, mice infected with rPRV-TK-WT displayed further attenuation, exhibiting a reduced mortality rate (80%) and a prolonged survival time relative to both PRV-WT and PRV-US3-KO groups..This additional attenuation may reflect differences in the expression timing and/or expression level of TK caused by insertion of the TK expression cassette into the Us3 locus, leading to differences from the native expression pattern of TK during the viral replication cycle.

**Fig 8:**
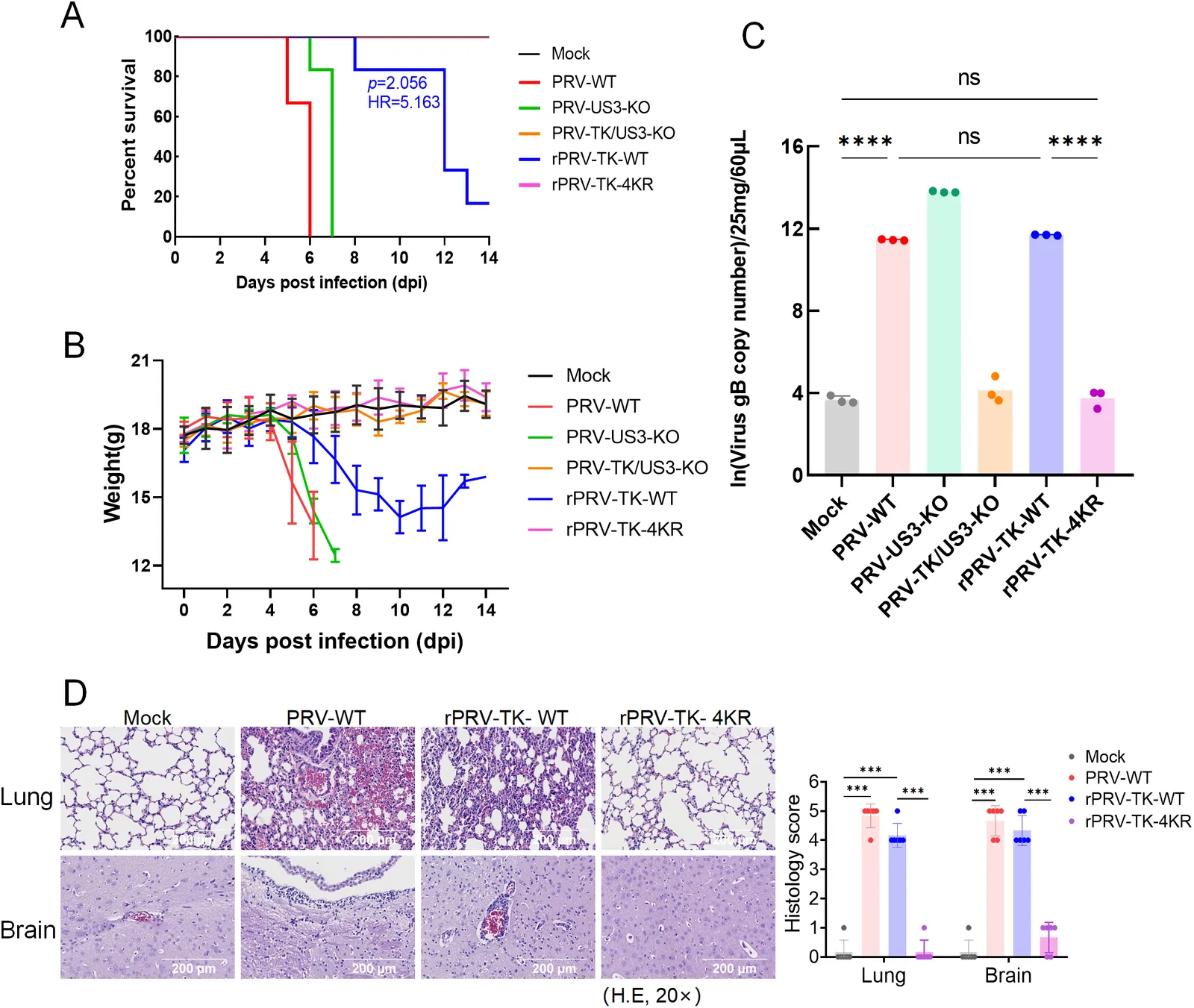
SUMOylation deficiency of TK attenuates PRV pathogenicity *in vivo.* Five-week-old female BALB/c mice (n = 6 per group) were intranasally infected with PRV-WT, PRV-US3-KO, PRV-TK/US3-KO, rPRV-TK-WT, rPRV-TK-4KR, or Mock control at 5 × 10^5^ TCID_50_ in 20 μL. **(A)** Kaplan–Meier survival curves were monitored for 14 days post-infection (dpi). Survival differences were analyzed using the log-rank (Mantel–Cox) test, and hazard ratios (HRs) were estimated using the Cox proportional hazards model in GraphPad Prism. **(B)** Body weight changes are presented as mean ± SD. **(C)** Surviving mice were euthanized at 14 dpi, whereas mice in the PRV-WT and PRV-US3-KO groups were euthanized at the moribund stage (6 and 7 dpi, respectively). Viral genome copies in brain tissues were quantified by absolute qPCR targeting the PRV gB gene. Data are presented as mean ± SD (n = 6 mice per group). **(D)** Representative hematoxylin and eosin **(H&E)**-stained sections of brain and lung tissues are shown. Histopathological lesions were evaluated independently in a blinded manner using a semiquantitative scoring system (0–5), based on the severity of inflammatory cell infiltration, perivascular cuffing, neuronal degeneration/necrosis, and tissue damage. Histopathological scores are presented as mean ± SD (n = 6 mice per group). Statistical significance was determined using one-way ANOVA followed by Tukey’s multiple-comparison test.

Remarkably, mice infected with the SUMOylation-deficient mutant rPRV-TK-4KR remained completely healthy throughout the observation period, showing no mortality (Fig 8A) and no obvious clinical symptoms comparable to those of the mock controls. Similar phenotypes were observed in the PRV-TK/US3-KO group. Consistent with the survival data, PRV-WT-, PRV-US3-KO-, and rPRV-TK-WT-infected mice exhibited substantial body weight loss, whereas mice infected with rPRV-TK-4KR or PRV-TK/US3-KO maintained stable body weights throughout the experiment (Fig 8B).

To further evaluate viral pathogenicity, brain viral loads and histopathological changes were analyzed. PRV-WT- and PRV-US3-KO-infected mice exhibited high viral loads in the brain, while rPRV-TK-WT reached comparable levels. Notably, the slightly higher viral genome copies observed in the PRV-US3-KO group may be partly related to the different sampling times, as these mice reached the moribund stage at 7 dpi, one day later than PRV-WT-infected mice (6 dpi). In contrast, viral genomes in rPRV-TK-4KR-infected mice remained at baseline levels, similar to those in PRV-TK/US3-KO- and mock-infected controls (Fig 8C). Histopathological examination revealed severe pulmonary hemorrhage and pronounced neuronal degeneration, necrosis, and atrophy in PRV-WT-infected mice. The rPRV-TK-WT group displayed reduced pulmonary damage but showed notable inflammatory infiltration and perivascular cuffing in the brain. By comparison, rPRV-TK-4KR-infected mice exhibited largely normal lung architecture and only minimal neuronal alterations (Fig 8D).

The attenuated phenotype of rPRV-TK-WT relative to PRV-WT, despite comparable viral loads, may reflect alterations associated with the recombinant viral backbone, such as disruption of the Us3 locus or ectopic, promoter-driven TK expression, which could partially modulate viral tropism or host responses. Importantly, the loss of pathogenicity observed with rPRV-TK-4KR is consistent with our *in vitro* findings that SUMOylation promotes TK nuclear localization and efficient replication in neuronal cells. Together, these data support a model in which TK SUMOylation facilitates neurotropic replication and disease progression, thereby acting as a critical determinant of PRV virulence *in vivo*.

## Discussion

Thymidine kinase (TK) is one of the most conserved proteins encoded by alphaherpesviruses and plays a pivotal role in viral DNA synthesis, particularly in neurons and other non-dividing cells where intracellular nucleotide pools are limited. Although the enzymatic function of TK has been extensively characterized, whether post-translational modifications regulate its biological activities has remained largely unknown. In the present study, we identify PRV TK as a SUMO-modified viral protein and demonstrate that SUMOylation is associated with efficient nuclear accumulation of TK, neuronal replication, viral reactivation, and pathogenicity. Importantly, we further show that disruption of the identified SUMOylation sites does not affect the intrinsic kinase activity of TK, supporting the conclusion that the observed phenotypes are attributable to altered SUMOylation rather than impaired catalytic function.

SUMOylation has emerged as an important regulatory mechanism during herpesvirus infection, modulating viral protein stability, localization, protein-protein interactions, and host immune evasion. Several herpesviral proteins, including ICP0, ICP4, EBNA3C, and K-bZIP, have been reported to undergo SUMO modification to optimize different stages of the viral life cycle [21–24]. However, little is known about whether alphaherpesvirus TK is regulated by SUMOylation. Here, we demonstrate that PRV TK can be modified by SUMO1, SUMO2, and SUMO3, with SUMO3 producing relatively stronger modification under overexpression conditions. Because endogenous SUMO1 is the predominant SUMO paralog detected in most mammalian cells and has been more extensively characterized in antiviral responses, subsequent mechanistic studies focused primarily on SUMO1. Importantly, endogenous SUMO1 conjugation of TK was confirmed both in transfected cells and during authentic PRV infection, indicating that TK SUMOylation is a physiological event.

Mutational analyses identified K242 and K267 as the principal residues contributing to TK SUMOylation. Structural modeling further suggested that although K16 and K56 are exposed on the protein surface, their local electrostatic environment may reduce SUMO conjugation efficiency. Because potential low-level modification at these sites could not be completely excluded, we generated the 4KR mutant to eliminate all predicted SUMO acceptor lysines and thereby minimize any residual SUMOylation in functional analyses. The 4KR mutant displayed markedly reduced SUMOylation while maintaining kinase activity comparable to wild-type TK. Together with the consistent effects on subcellular localization, neuronal replication, reactivation, and virulence, these data support a functional role for TK SUMOylation.

To further explore the evolutionary conservation of this regulatory mechanism, we compared TK amino acid sequences from several representative alphaherpesviruses, including HSV-1, BHV-1, EHV-1, and MDV. Sequence alignment revealed that the predicted SUMOylation-associated lysine residues are partially conserved across these viruses (S4A Fig). K16 is completely conserved, whereas K242 and K267 are conserved in most alphaherpesviruses except HSV-1 and BHV-1, respectively. In contrast, K56 is less conserved and is present only in PRV and EHV-1. Among the viruses analyzed, PRV and EHV-1 display the highest conservation of all four lysine residues. Interestingly, HSV-1 TK exhibited substantially weaker SUMOylation than PRV TK in our supplementary experiments (S4B Fig), which may be associated with the substitution of K56 and K242 by arginine. Although this interpretation remains speculative, these comparative analyses suggest that SUMOylation of TK may represent a partially conserved regulatory mechanism among alphaherpesviruses, with potential species-specific differences in SUMO acceptor site utilization.

One of the major findings of this study is that SUMOylation promotes efficient nuclear accumulation of TK. Both mutation of SUMOylation sites and enzymatic deSUMOylation by SENP1 or SENP2 resulted in redistribution of TK from the nucleus to the cytoplasm, indicating that SUMOylation dynamically regulates TK subcellular localization. Unlike many nuclear proteins, TK lacks a canonical nuclear localization signal, suggesting that its nuclear trafficking may depend on non-classical mechanisms. Increasing evidence indicates that SUMOylation can regulate nucleocytoplasmic transport by recruiting proteins containing SUMO-interacting motifs (SIMs), thereby promoting assembly of transport complexes or stabilizing protein retention within specific nuclear compartments. Previous studies have shown that SUMO-dependent interactions participate in the nuclear transport of multiple cellular proteins through cooperation with importins and components of the nuclear pore complex [25,26]. For example, members of the importin-α family, RanBP2 (Nup358), and other nucleoporins contain functional SIMs or interact preferentially with SUMO-modified cargoes, thereby facilitating nuclear import or retention [27]. Beyond regulating protein trafficking, SUMOylation is also a well-established mechanism for modulating protein-protein interactions by creating docking sites for SIM-containing proteins or altering the affinity of existing protein complexes. Therefore, SUMOylation of PRV TK may not only facilitate its interaction with host nuclear transport factors but also influence its association with viral or cellular proteins involved in viral DNA replication and replication compartment organization. In viral infections, SUMO-dependent protein interactions have also been implicated in the assembly of viral replication compartments and the reorganization of promyelocytic leukemia (PML) nuclear bodies [28–33]. Based on these observations, we speculate that SUMOylated TK may recruit one or more SIM-containing host factors, potentially including importins or nuclear pore-associated proteins, to facilitate its nuclear translocation and/or retention within viral replication compartments. Whether SUMOylation directly regulates the interaction of TK with specific host or viral proteins remains to be determined. Identification of these SUMO-dependent interacting partners will be an important direction for future studies and will help further elucidate the molecular mechanism by which SUMOylation regulates TK function during PRV infection.

Interestingly, loss of TK SUMOylation produced striking phenotypes in neuronal N2a cells but had little detectable effect on viral replication in PK15 epithelial cells. These observations are consistent with the well-established biological role of herpesvirus TK. In rapidly dividing epithelial cells, abundant endogenous deoxythymidine triphosphate (dTTP) pools can largely compensate for reduced nuclear TK function. In contrast, neurons possess relatively limited nucleotide reserves and therefore rely more heavily on virus-encoded TK to support efficient viral DNA synthesis. Consequently, impaired nuclear accumulation of SUMO-deficient TK is expected to exert a much greater impact on viral replication in neuronal cells. Importantly, although viral growth kinetics were similar in PK15 cells, SUMOylation-deficient TK still exhibited reduced nuclear localization in these cells, indicating that altered localization represents a direct consequence of impaired SUMOylation rather than a secondary effect of reduced viral replication. Furthermore, immunofluorescence analysis of recombinant viruses in N2a cells confirmed that the 4KR mutation similarly impaired TK nuclear accumulation in neuronal cells, demonstrating that this regulatory mechanism is conserved across cell types.

Previous studies have established that TK is dispensable for establishment of latency but essential for efficient viral reactivation [8,9]. Consistent with these observations, our *in vitro* latency/reactivation model demonstrated that deletion of TK completely abolished viral reactivation. Importantly, viral genome copy numbers, viral DNA levels, viral gene transcription, viral protein expression, and infectious virus production were all similarly suppressed among PRV-WT, PRV-US3-KO, rPRV-TK-WT, and rPRV-TK-4KR during the 42°C latency-induction phase, indicating comparable establishment of the latent-like state before reactivation. Following temperature shift, however, only viruses expressing SUMOylation-competent TK efficiently resumed productive replication, whereas the SUMO-deficient virus remained largely unable to reactivate. These findings suggest that the reactivation defect is unlikely to result simply from reduced replication capacity prior to latency induction but is instead associated with impaired TK function during the reactivation process.

Our animal experiments further support the biological importance of TK SUMOylation during PRV infection *in vivo*. Because the recombinant viruses were generated by insertion of the TK expression cassette into the Us3 locus, we included PRV-US3-KO as an additional control to distinguish the effects of Us3 deletion from those of TK SUMOylation. Consistent with previous reports, deletion of Us3 attenuated neurovirulence. Nevertheless, rPRV-TK-4KR remained substantially less virulent than rPRV-TK-WT, exhibiting complete survival, reduced tissue viral loads, and markedly alleviated histopathological lesions. We also observed that rPRV-TK-WT displayed modestly lower virulence than PRV-US3-KO. Because sequencing confirmed the integrity of the engineered genomic region, we speculate that this additional attenuation is more likely attributable to altered timing or expression level of TK caused by ectopic insertion into the Us3 locus rather than unintended mutations. This represents a limitation of the current recombinant virus design and will be addressed in future studies through the development of alternative genome-editing strategies.

Several limitations of this study should be acknowledged. First, our analysis focused on a small set of PRV proteins selected based on their known enzymatic or regulatory functions and was not intended to systematically identify SUMO substrates across the PRV proteome. Therefore, additional SUMO-modified viral proteins may remain to be identified. Second, although we demonstrate that TK is directly SUMOylated and that this modification regulates its nuclear localization and biological functions, the SUMO-dependent host factors that mediate these processes remain unknown. Identifying these interacting proteins will be important for elucidating the molecular mechanism underlying TK nuclear trafficking. Finally, the functional role of TK SUMOylation was primarily investigated using the N2a temperature-shift latency-like/reactivation model and an acute mouse infection model. Although these models recapitulate key features of PRV neuronal infection, latency/reactivation, and pathogenesis, further validation in primary neurons and in vivo latency models will strengthen the physiological relevance of our findings. In addition, while loss of TK SUMOylation markedly reduced viral burden in the brain, the present study does not distinguish whether this results primarily from impaired neuroinvasion or reduced viral replication after entry into the central nervous system. Future studies combining analyses of viral dissemination with immunohistochemical detection of viral antigens will help further define the role of TK SUMOylation during PRV neuroinvasion and CNS infection.

In summary, our study identifies TK as a previously unrecognized SUMO substrate in PRV and demonstrates that SUMOylation contributes to efficient nuclear accumulation of TK without affecting its intrinsic kinase activity. This post-translational modification promotes viral replication in neuronal cells, facilitates efficient reactivation from latency, and contributes to viral pathogenicity in vivo. These findings reveal an additional layer of regulation governing alphaherpesvirus TK function and provide new insights into how host SUMOylation machinery modulates the viral life cycle.

## Materials and methods

### Ethics statement

All animal research was performed in compliance with the Beijing Laboratory Animal Welfare and Ethics guidelines, as issued by the Beijing Administration Committee of Laboratory Animals, and the China Agricultural University (CAU) Institutional Animal Care and Use Committee guidelines (ID: SKLAB-B-2010–003) approved by the Animal Welfare Committee of CAU. All experiments with live viruses were performed in Biosafety Level II laboratory (BSL-2) in CAU. All animal research involved in this study was approved by the Animal Welfare Committee of CAU (No.AW03503202-2-2).

### Cell culture and viruses

HEK293 cells (human embryonic kidney cells), HeLa cells (human cervical carcinoma cells), PK15 cells (porcine kidney epithelial cells) and N2a cells (mouse neuroblastoma cells) were cultured in Dulbecco’s minimum essential medium (DMEM) supplemented with 10% (v/v) fetal bovine serum (FBS) and penicillin (100U/mL)-streptomycin (100 μg/mL). The cell culture medium, serum, and antibiotics were purchased from Invitrogen. All cells were maintained at 37 °C in 5% CO_2_.

The PRV (Bartha-K61) and HSV-1 (KOS strain) was purchased from Weike Biotech Co.,Harbin, China. The recombinant PRV TK-knockout virus (PRV-TK-KO) was generated by CRISPR/Cas9 system as described previously [34]. The viruses were propagated and titrated on PK15 cells. The viral titer was determined and expressed as the 50% tissue culture infective dose (TCID50)/ml.

### Antibodies and reagents

The antibodies against viral proteins used in this study, including IE180, VP5, US3, TK and gB of PRV were raised in mice following a standard procedure [35]. The following antibodies were used for immunoprecipitation, Immunofluorescence staining and western blotting: anti-Flag (F1804) and anti-Flag M2 beads (A2220) from Sigma-Aldrich (St. Louis, MO, USA). Anti-α-Tubulin (PM054) from MBL (Nagoya, Japan). Anti-GFP antibody (sc-9996) from Santa Cruz Biotechnology (Santa Cruz, CA, USA). Anti-HA antibody (3F10) Roche (Basel, Switzerland). Anti-SUMO1 antibody (MA5–70565 and MA5–35272) from Thermo Fisher Scientific (Waltham, MA, USA). Anti-β-Actin antibody (A5441) from Sigma-Aldrich (St. Louis, MO, USA). Anti-Lamin B1 antibody (ab65986) from Abcam (Cambridge, UK). Anti-Histone H3 antibody (9715) from Cell Signaling Technology (Danvers, MA, USA). Horseradish peroxidase (HRP)-conjugated goat anti-mouse and anti-rabbit secondary antibodies, FITC-conjugated goat anti-mouse and anti-rabbit secondary antibodies, TRITC-conjugated goat anti-mouse secondary antibody, and DAPI (4′,6-diamidino-2-phenylindole) were purchased from DingGuoChangSheng Biotech Co., Ltd., Beijing. Cycloheximide (CHX) was from Amresco, and MG132 proteasome inhibitor was purchased from MCE.

### Plasmids and transfection

The full-length TK gene was amplified from the Bartha-K61 genome and cloned into the pRK5 vector with an N-terminal Flag tag. Site-directed mutagenesis was performed using overlap extension PCR to substitute lysine residues (K16, K56, K242, and K267) with arginine, either individually, in combination, or simultaneously (4KR mutant). All primers used for amplification of the wild-type TK gene and generation of mutant constructs are listed in Table 1.

**Table 1:** Primers used for TK wild-type and mutant-related gene amplification.

Primer	Sequence (5’-3’)
UL23-F: UL23-R: K16R-F: K16R-R: K56R-F: K56R-R: K242R-F: K242R-R: K267R-F: K267R-R:	GGATCCATGCGCATCCTCCGGATCTACCTC AAGCTTTCACACCCCCATCTCCGACGTGAA GCCTACGGCACCGGCAGGAGCACCACGGCCCGG CCGGGCCGTGGTGCTCCTGCCGGTGCCGTAGGC GATGCGCAGACCCGGAGGCAGAACGGCAGCCTG CAGGCTGCCGTTCTGCCTCCGGGTCTGCGCATC CTCTTCGGCGCGTACAGGGCGCCCGAGCTCTGC GCAGAGCTCGGGCGCCCTGTACGCGCCGAAGAG GACGCGCTCGTGGCCAGGCTGCTGCCGCTGCGC GCGCAGCGGCAGCAGCCTGGCCACGAGCGCGTC

Plasmids were transiently transfected into HEK293T cells using Chemifect transfection reagent (Fengrui Biotechnology, Beijing, China) and into PK15 cells using M5 Hiper Lipo2000 transfection reagent (Invitrogen, Thermo Fisher Scientific, Waltham, MA, USA), following the manufacturers’ instructions.

### Immunoprecipitation and western blotting

Immunoprecipitation (IP) and western blotting (WB) were performed as previously described [36] with minor modifications. Briefly, 24–48 h post-transfection, cells were harvested and lysed in IP lysis buffer (50 mM Tris–HCl, pH 8.0, 150 mM NaCl, 1% Triton X-100, 10% glycerol, 1 mM DTT) supplemented with protease inhibitor cocktail (Roche, Basel, Switzerland) and 20 mM N-ethylmaleimide (NEM) where indicated. Cell lysates were clarified by centrifugation at 12,000 × g for 10 min at 4°C and pre-cleared with protein G Sepharose beads (GE Healthcare, Chicago, IL, USA) for 2 h at 4°C with rotation.

For immunoprecipitation, clarified lysates were incubated with the indicated primary antibodies overnight at 4°C, followed by incubation with protein G Sepharose beads for an additional 2–4 h. Alternatively, anti-Flag M2 affinity gel (Sigma-Aldrich, St. Louis, MO, USA) was used directly for 4 h at 4°C. The beads were washed extensively (3–5 times) with lysis buffer and then boiled in SDS loading buffer for 5 min to elute bound proteins.

For detection of endogenous SUMOylated TK under denaturing conditions, cells were lysed in denaturing lysis buffer containing 1% SDS, 20 mM N-ethylmaleimide (NEM), and protease inhibitor cocktail, followed by heating at 95°C for 10 min to disrupt non-covalent protein-protein interactions while preserving covalent SUMO conjugates. Lysates were subsequently diluted tenfold with NP-40 lysis buffer to reduce the SDS concentration to 0.1% prior to immunoprecipitation. Equal amounts of protein were incubated overnight at 4°C with anti-SUMO1 antibody, followed by incubation with Protein A/G agarose beads. After extensive washing, bound proteins were eluted in SDS sample buffer, resolved by SDS-PAGE, and analyzed by immunoblotting using anti-TK or anti-Flag antibody as indicated.

For western blot analysis, whole-cell lysates were prepared using NP-40 lysis buffer (50 mM Tris–HCl, pH 8.0, 150 mM NaCl, 1% NP-40, 10% glycerol, 20 mM NaF, 1 mM DTT) supplemented with protease inhibitors. Protein samples were separated by SDS–PAGE and transferred onto nitrocellulose membranes (GE Healthcare). Membranes were blocked with 5% non-fat milk in PBST (PBS containing 0.1% Tween-20) for 1 h at room temperature and incubated with the indicated primary antibodies (1:1000 dilution) overnight at 4°C. After washing, membranes were incubated with appropriate HRP-conjugated secondary antibodies (1:10000 dilution) for 1 h at room temperature. Protein bands were visualized using enhanced chemiluminescence (ECL) reagents (Thermo Fisher Scientific, Waltham, MA, USA) according to the manufacturer’s instructions.

### ADP-Glo kinase assay

Kinase activity of TK proteins was determined using the ADP-Glo Kinase Assay Kit (Promega, Cat.No. V6930) according to the manufacturer’s instructions. Briefly, Flag-TK-WT, Flag-TK-K242/267R, and Flag-TK-4KR proteins were transiently expressed in HEK293T cells and purified by anti-Flag affinity immunoprecipitation. Equal amounts of purified proteins were incubated with ATP and thymidine substrate (Sigma T1895) in kinase reaction buffer at room temperature for 40 min. Following completion of the kinase reaction, ADP-Glo Reagent was added to terminate the reaction and deplete the remaining ATP. Subsequently, Kinase Detection Reagent was added to produce a luciferase-based luminescent signal proportional to kinase activity. Each experiment was performed in triplicate.

### Immunofluorescence staining

Cells were fixed with 2% of paraformaldehyde for 15 min, permeabilized with 0.2% Triton X-100 for 15 min on ice, and blocked with 1% BSA (bovine serum albumin) in PBS for 30 min. The cells were then incubated with specific primary antibodies (1:200 dilution) for 1 h at room temperature, followed by FITC (fluorescein isothiocyanate)- or TRITC (tetramethyl rhodamine isothiocyanate)-conjugated secondary antibodies (1:1000 dilution) for 30 min. Nuclei were stained with DAPI for 3–5 min. Images were captured using a Leica TCS SP8 laser scanning confocal microscope (Leica Microsystems, Wetzlar, Germany). The captured images were processed and analyzed using SPOT software (Nikon).

### Construction of recombinant transfer plasmids and sgRNA expression plasmids

The PRV US3 gene was selected as the insertion site for the wild-type TK (TK-WT) and its quadruple mutant (TK-4KR; K16R, K56R, K242R, K267R). Recombinant viruses were generated using a CRISPR/Cas9-mediated genome editing strategy. Briefly, TK-WT or TK-4KR expression cassettes was inserted into nucleotides 1–220 of the US3 gene through Cas9-induced double-strand DNA cleavage followed by CRISPR/Cas9-mediated homologous recombination (HR). Recombinant transfer plasmids containing the TK-WT or TK-4KR expression cassette were constructed by sequentially assembling the US3 homologous region (nucleotides 1–220), the cytomegalovirus (CMV) promoter, an RFP reporter gene, and the pre-constructed TK-WT or TK-4KR fragment into the pCloneEZ-Blunt-Amp-HC vector (Shanghai Xuanya Biotechnology Co., Ltd., China). sgRNA sequences targeting the US3 locus (nucleotides 1–220) were designed using the CRISPR design tool CRISPOR (http://crispor.tefor.net) and cloned into the pUC19 vector to generate sgRNA expression plasmids. The sgRNA expression plasmid and the Cas9 expression plasmid were co-transfected with the recombinant transfer plasmid into PRV-infected cells to facilitate targeted cleavage of the US3 locus and insertion of the TK expression cassette. Recombinant viruses were identified based on RFP fluorescence, purified by plaque isolation, and further verified by PCR and Sanger sequencing. Primers used for construction and validation of recombinant transfer plasmids and sgRNA expression plasmids are listed in Table 2.

**Table 2:** Primers used for the construction of recombinant transfer plasmids and sgRNA expression plasmids.

Primer	Sequence (5’-3’)
US3-F: US3-R: CMV-F: CMV-R: RFP-F: RFP-R: sgRNA1: sgRNA2:	GCCCTTCGCGTCGCGGACGCCGACGCCGGAATCCCC CAAACGGGTTTATTGCAGCGCCCGGCCGGCGGGCCA CTGCAATAAACCCGTTTGTCATACTATCGCATCGTT GATCCGGAGGATGCGCATGGTGGCACCGGTAGCGCT GAAGAAAACCCCGGTCCTATGGCCTCCTCCGAGGAC ATGGCTGATTATGATCAGTTATCTAGATCCGGTGGA ACGCCGGAATCCCCGACGAG CGTCTTCGTGGCCCGCCGGC

### Rescue and validation of recombinant viruses

Genomic DNA was extracted from the recombinant PRV TK knockout virus (PRV-TK-KO) using a genomic DNA extraction kit (Takara). PK15 cells were co-transfected with PRV-TK-KO genomic DNA, sgRNA expression plasmids, a Cas9 expression plasmid, and either the TK-WT or TK-4KR recombinant transfer plasmid, according to the manufacturers’ instructions for Lipofectamine 2000. Viral supernatant was harvested 72 hours post-transfection, and single-clone viruses were isolated via end-point dilution assay (10^−1^-10^−7^).

### Latency-like infection and reactivation assay

The *in vitro* latency-like/reactivation model in N2a cells was established as previously described with minor modifications [37]. Briefly, N2a cells were infected with virus at a multiplicity of infection (MOI) of 1 and subsequently cultured at either 37 °C or 42 °C, with medium replaced every 24 h. To establish latency, infected cells were maintained at 42 °C for 3 days, followed by a shift to 37 °C for an additional 5 days to induce viral reactivation.

Cells and culture supernatants were collected at indicated time points (0–8 days post-infection) for downstream analyses. Specifically, the extracellular PRV gB DNA copy numbers in culture supernatants were determined by qPCR, the IE180 and LAT transcript levels in infected cells were determined by RT-qPCR, the intracellular PRV DNA copy numbers in infected cells were determined by qPCR, the viral protein expression in cell lysates was analyzed by immunoblotting, and the infectious virus titers in culture supernatants were determined by TCID50 assay on PK15 cells. including viral DNA quantification, total RNA extraction, TCID_50_ assays, and viral protein detection. All samples were immediately stored at −80 °C until further processing. Each experimental condition was performed with three independent biological replicates.

Viral DNA from culture supernatants was extracted using the FastPure Viral DNA Mini Kit (RC311–01, Vazyme Biotech Co., Ltd., Nanjing, China) according to the manufacturer’s instructions. Purified DNA samples were stored at −20 °C prior to quantitative analysis.

### Experiments with mice

Animal care and experimental protocols were approved by the Animal Welfare Committee of China Agricultural University. Five-week-old specific-pathogen-free (SPF) female BALB/c mice were randomly assigned to the indicated experimental groups (n = 6). Mice were intranasally inoculated with 5 × 10^5 TCID_50_ of the indicated PRV strains in 20 μL DMEM or with an equal volume of DMEM as the control. Mice were monitored daily for clinical signs and body weight changes. Moribund mice and surviving mice at the endpoint of the experiment (14 days post-infection) were humanely euthanized.

At necropsy, organs including the brain and lungs were collected. For viral load analysis, tissues were weighed, homogenized in sterile PBS (10% [w/v]) containing antibiotics, and clarified by centrifugation at 12,000 × g for 10 min at 4°C. The resulting supernatants were collected for viral genome quantification by quantitative PCR. For histopathological analysis, tissue samples were fixed in 4% paraformaldehyde, embedded in paraffin, sectioned, and stained with hematoxylin and eosin (H&E) according to standard procedures.

### Quantitative PCR (q-PCR)

Total genomic DNA was extracted from mouse brain tissues using a genomic DNA mini extraction kit (Beyotime Biotechnology, China) following the manufacturer’s instructions. Quantitative PCR (qPCR) was performed using Fast SYBR Mixture (Cowin Biotech, China) on a ViiATM 7 Real-Time PCR System (Applied Biosystems). Each reaction was performed in a 20 μL volume containing 10 μL of Fast SYBR Mixture, 0.4 μM of each primer, and 2 μL of template DNA. The amplification program consisted of an initial denaturation step at 95°C for 10 min, followed by 40 cycles of denaturation at 95°C for 15 s and annealing/extension at 60°C for 30s. A melting curve analysis was performed at the end of each reaction to confirm amplification specificity. All samples were analyzed in technical triplicates. Viral genome copy numbers were calculated based on the Ct values obtained from qPCR analysis. Primers used for qPCR are listed in Table 3.

**Table 3:** Primers used for qPCR.

Primer	Sequence (5’-3’)
gB-F: gB-R: mGAPDH-F: mGAPDH-R: IE180-F: IE180-R: LAT-F: LAT-R:	AACCACCGGCGCTACTTTAA GCTGGATCTCGCTGTAGTCC AGCCAAAAGGGTCATCATCTC GGACTGTGGTCATGAGCCCTTC TCGTGCGCCTCATCTACAG TGGCAGAACTGGTTGAAGCG CCGCGATACATCCAACAC GAACAGCCTCTGGATGAC

### Statistical analysis

All statistical analyses were performed using GraphPad Prism 9.0 (GraphPad Software, San Diego, CA, USA). Unless otherwise indicated, all in vitro experiments were independently repeated at least three times (biological replicates). For qPCR analyses, each biological sample was analyzed in technical triplicate. Data are presented as the mean ± standard deviation (SD). Comparisons between two groups were performed using an unpaired two-tailed Student’s t test, whereas comparisons among multiple groups were performed using one-way analysis of variance (ANOVA) followed by the appropriate post hoc multiple-comparison test. The number of biological replicates (n) is indicated in the corresponding figure legends. For animal experiments, six mice were included in each experimental group (n = 6). A P value < 0.05 was considered statistically significant.

## Supporting information

S1 Fig Sequence validation of the TK expression cassette insertion and engineered SUMOylation-site mutations in recombinant PRV strains. (A) Schematic representation of the TK expression cassette inserted into the Us3 locus of the parental PRV-TK-KO genome, showing the 5′ and 3′ Us3 insertion junctions, the CMV promoter, RFP reporter, and TK expression cassette.(B) Alignment of the TK coding sequences from rPRV-TK-WT and rPRV-TK-4KR. The four engineered lysine-to-arginine substitutions, K16R, K56R, K242R, and K267R, are highlighted. Sequence identities are indicated above the corresponding sequences. The alignment confirms the correct insertion of the TK expression cassette and the expected mutations in rPRV-TK-4KR.
(TIF)

S2 Fig Effect of TK deletion on the establishment of PRV latency. N2a cells were infected with virus at a multiplicity of infection (MOI) of 1 and cultured at either 37°C or 42°C, with medium replaced every 24 h. Latency was established by maintaining infected cells at 42°C for 3 days. Cells and culture supernatants were collected at the indicated time points for analysis of viral gB DNA copy number (A), transcription levels of IE180 (B) and LAT (C), viral DNA quantification (D), infectious virus titers determined by TCID₅₀ assay (E), and viral protein expression (F). Data are presented as mean ± SD from three independent experiments. Statistical significance was determined using one-way ANOVA (****p < 0.0001).
(TIF)

S3 Fig Effect of TK deletion on PRV reactivation from latency. N2a cells were infected with virus at a multiplicity of infection (MOI) of 1 and cultured at either 37°C or 42°C, with medium replaced every 24 h. Latency was established by incubation at 42°C for 3 days, followed by a shift to 37°C for an additional 5 days to induce viral reactivation. Cells and culture supernatants were collected at the indicated time points for analysis of viral gB DNA copy number (A), transcription levels of IE180 (B) and LAT (C), viral DNA quantification (D), infectious virus titers determined by TCID₅₀ assay (E), and viral protein expression (F). Data are presented as mean ± SD from three independent experiments. Statistical significance was determined using one-way ANOVA (*p < 0.05, ****p < 0.0001).
(TIF)

S4 Fig Conservation analysis of TK SUMOylation sites among alphaherpesviruses. A. Multiple sequence alignment of the amino acid regions containing the identified lysine SUMOylation sites of TK from pseudorabies virus (PRV), herpes simplex virus 1 (HSV-1), bovine herpesvirus 1 (BHV-1), equine herpesvirus 1 (EHV-1), and Marek’s disease virus (MDV). Conserved lysine residues corresponding to the PRV TK SUMOylation sites are indicated. (B) Comparative analysis of SUMOylation of HSV-1 TK and PRV TK. HEK293 cells were co-transfected with plasmids expressing Flag-tagged HSV-1 TK or PRV TK together with HA-Ubc9 and HA-SUMO1 or HA-SUMO2. At 24 h post-transfection, whole-cell lysates and Flag M2 affinity-purified proteins were analyzed by immunoblotting with anti-HA and anti-Flag antibodies to assess TK SUMOylation.
(TIF)

S5 Fig The raw images of WB and IF. (PDF)

S1 Table Source data for main figure plots and graphs. (XLSX)
