# Supplementary material for: SUMOylation of pseudorabies virus thymidine kinase regulates its nuclear localization and facilitates neuronal replication and reactivation
Source: PLoS Pathog. 2026 Oct 5;22(10):e1014653. doi: 10.1371/journal.ppat.1014653 (PMC13649132; doi:10.1371/journal.ppat.1014653)

Figure 1A

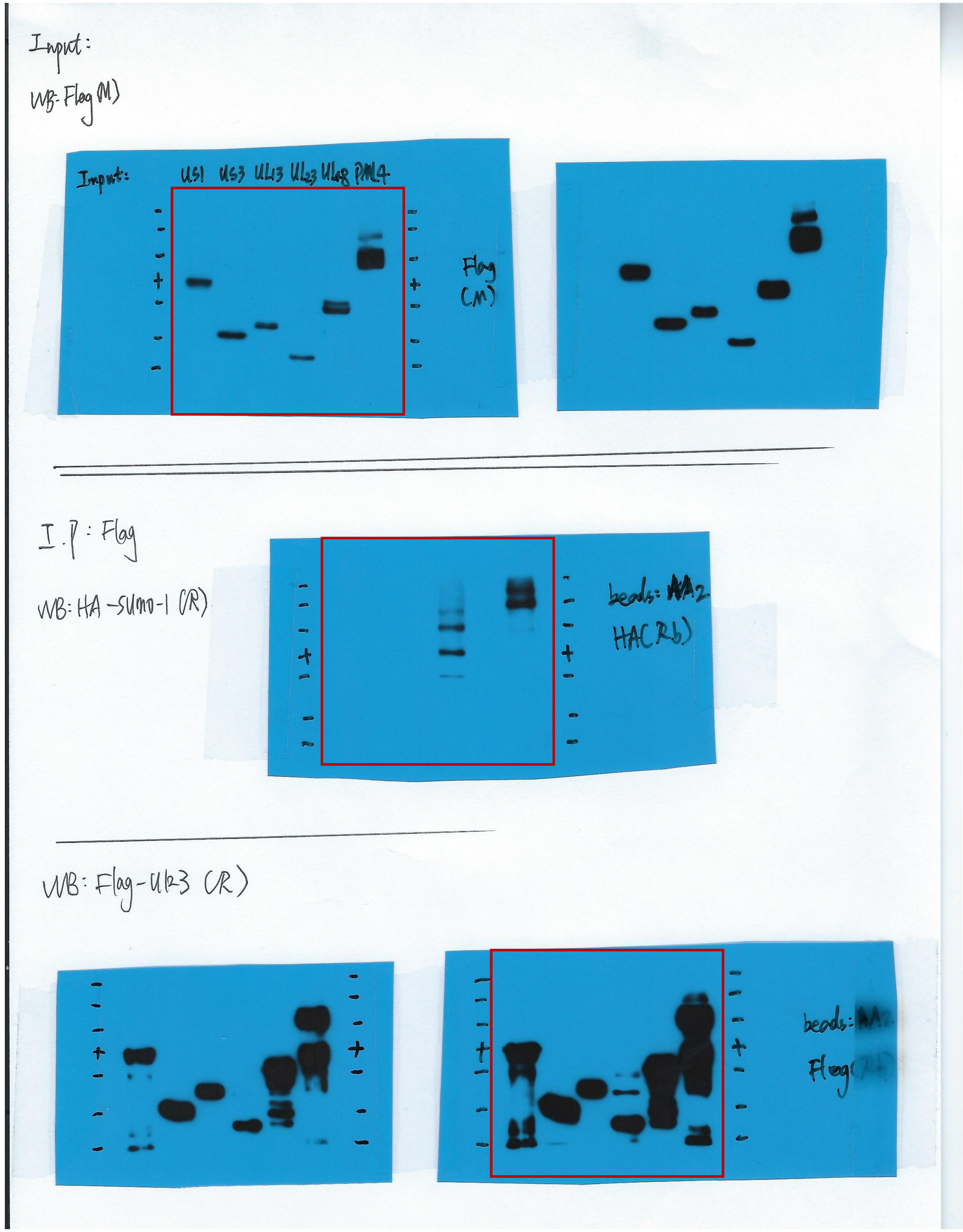

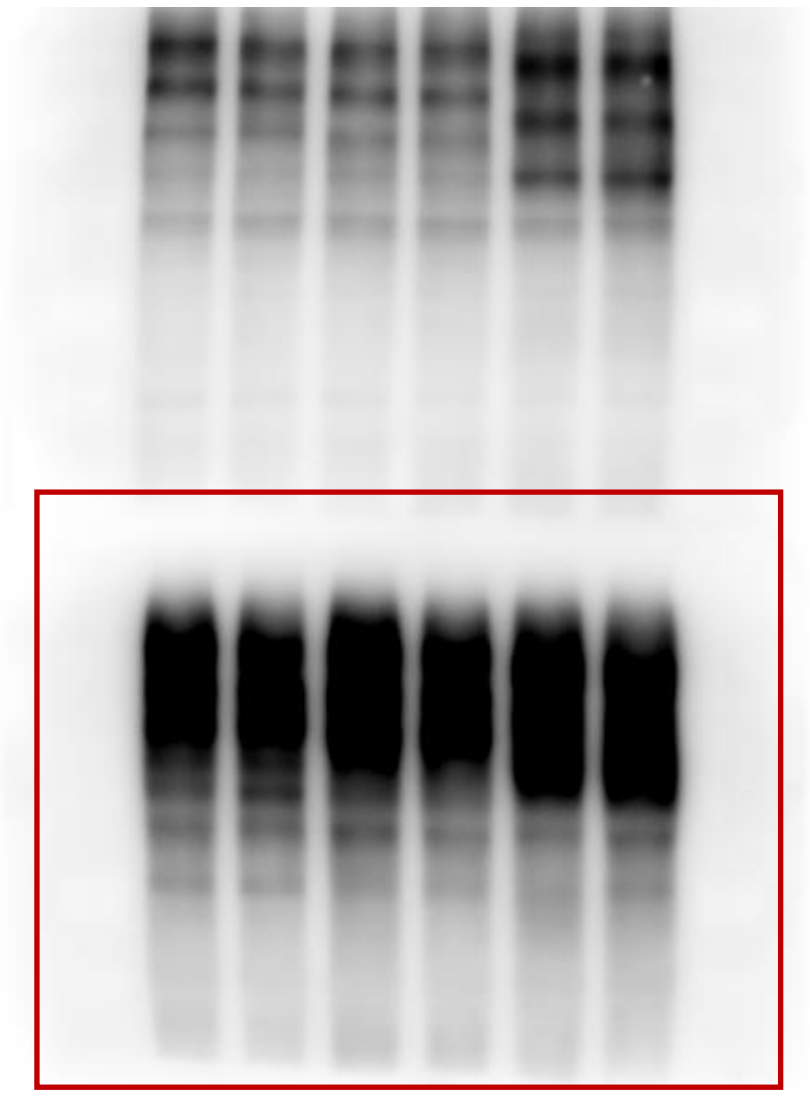

Figure 1B

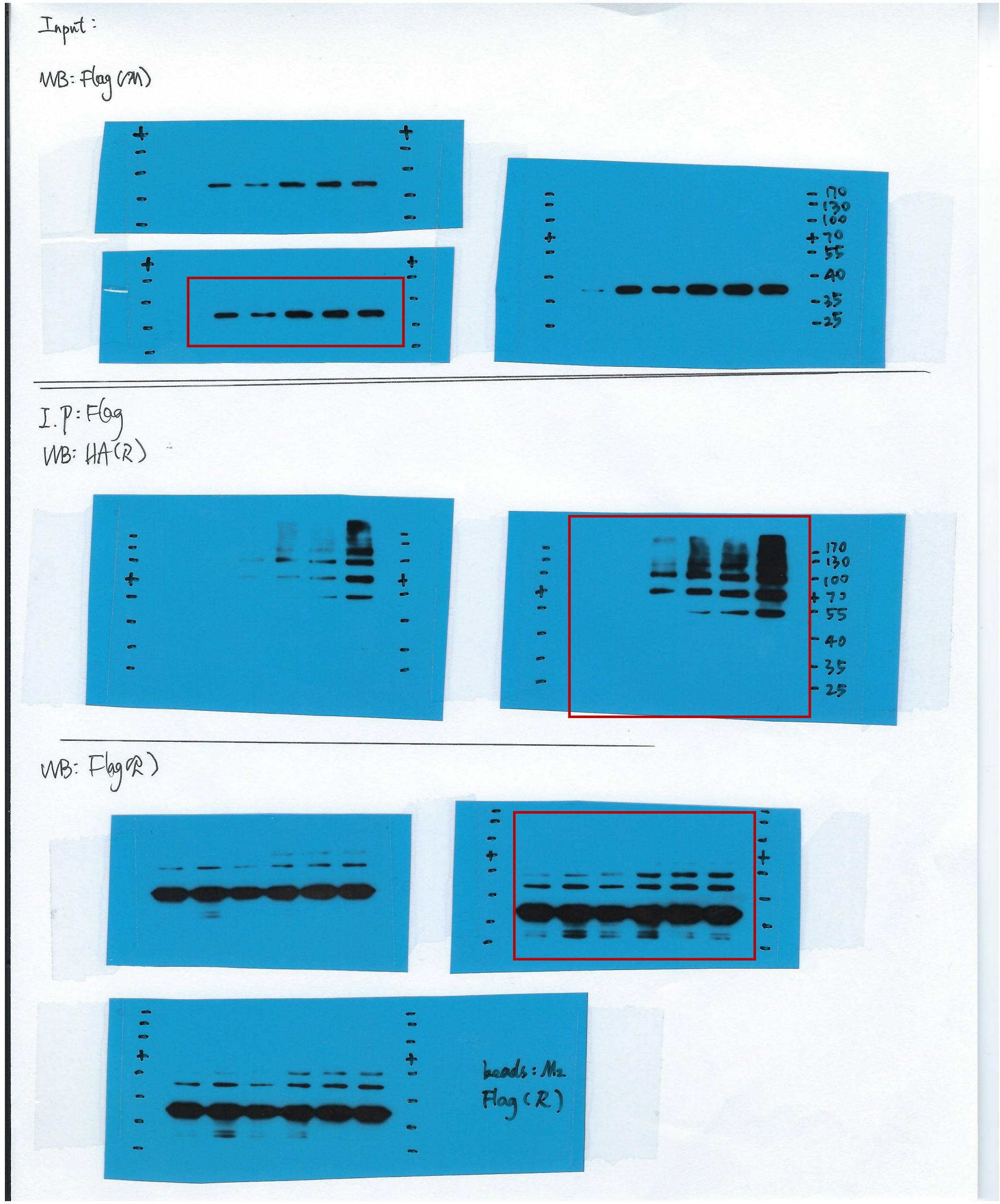
Input:
WB: Flag (M)
170
130
100
70
55
40
35
25
I.P: Flag
WB: HA (R)
170
130
100
70
55
40
35
25
WB: Flag (R)
beads: M2
Flag (R)

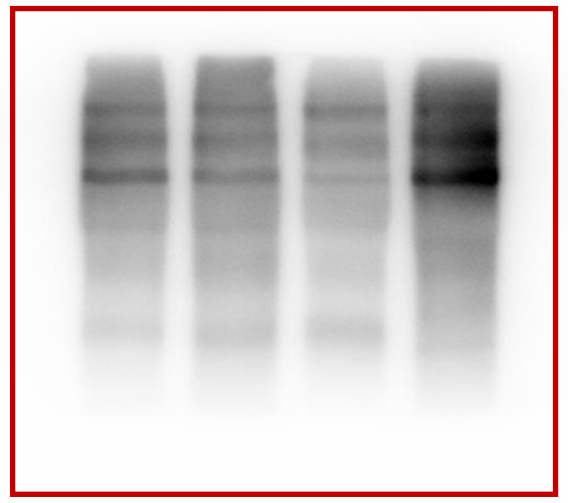

Figure 1C

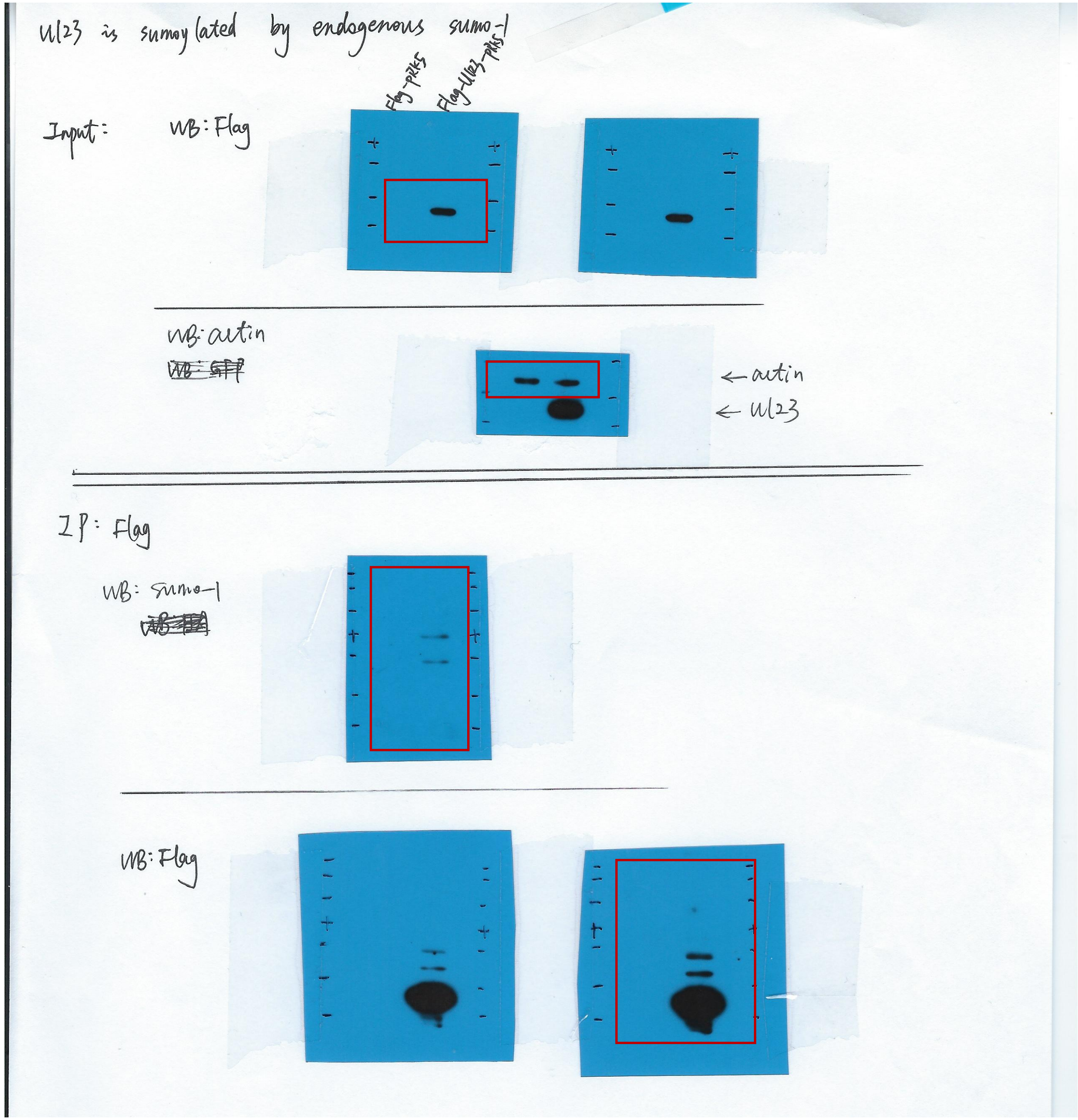
Ul23 is sumoylated by endogenous sumo-1
Flag-pRK5
Flag-Ul23-pRK5
Input:
WB: Flag
WB: actin
WB: GFP
← actin
← Ul23
IP: Flag
WB: sumo-1
WB: Flag

Figure 1D

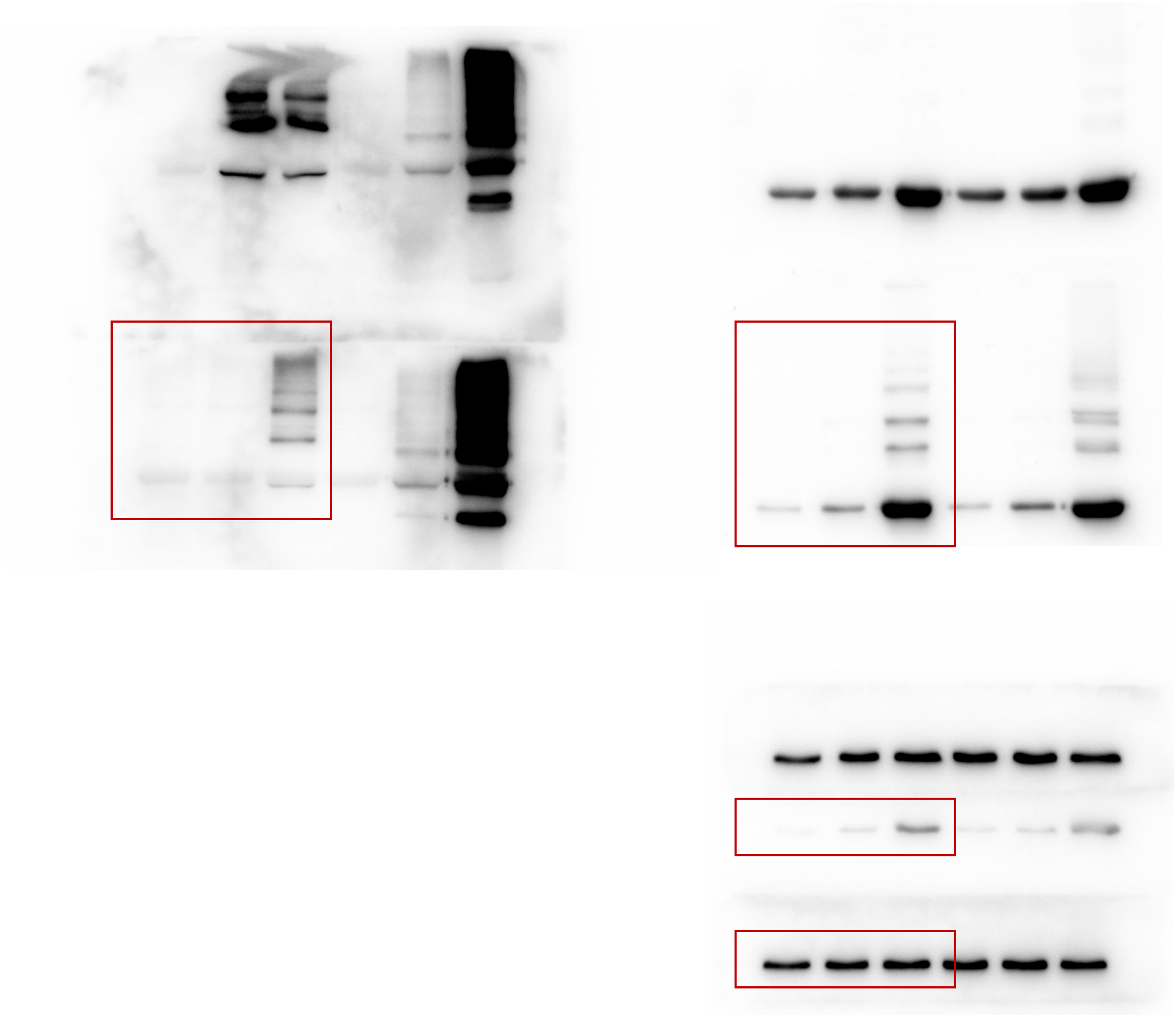

Figure 2B

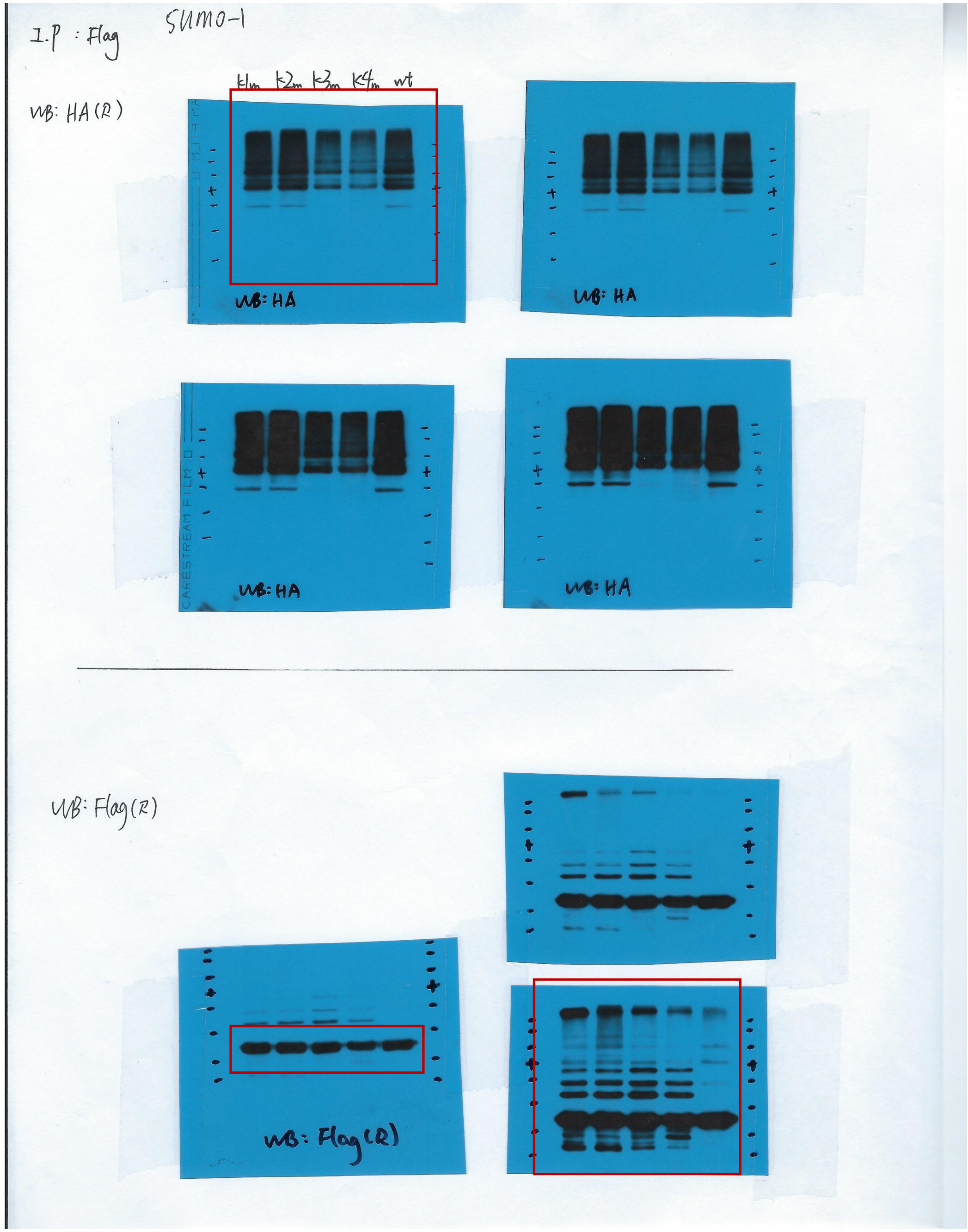
I.P : Flag
SUMO-1
WB: HA(R)
K1m K2m K3m K4m wt
WB: HA
WB: HA
WB: HA
WB: HA
WB: Flag(R)
WB: Flag(R)

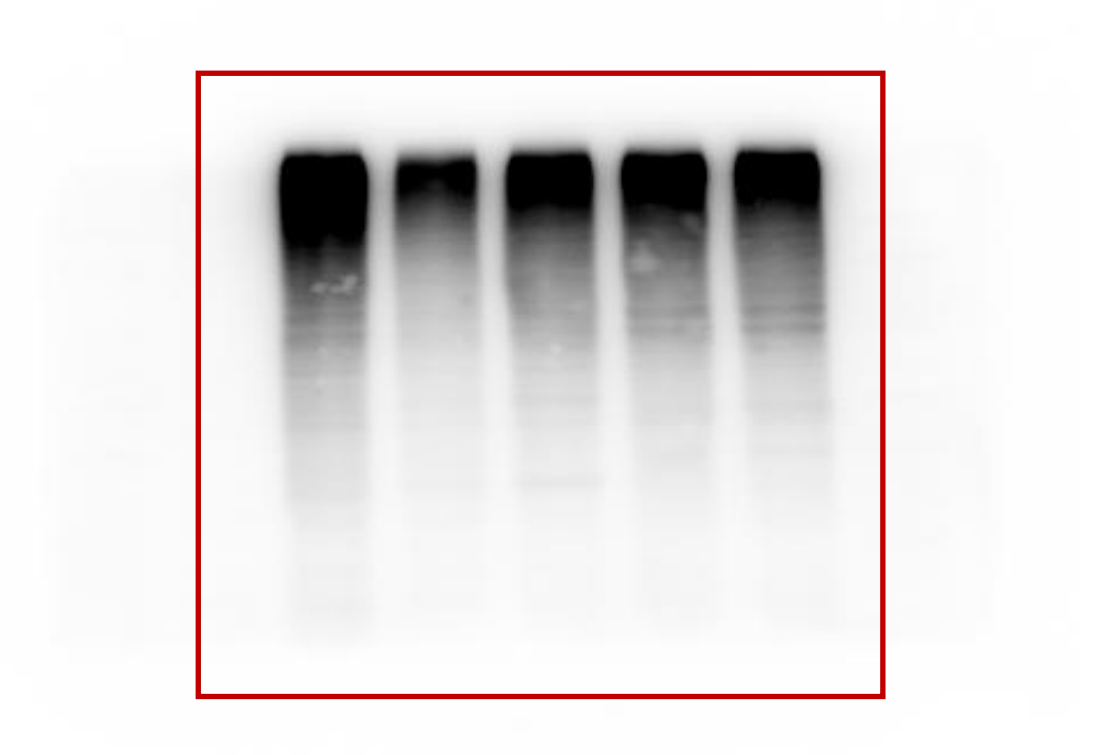

Figure 2C

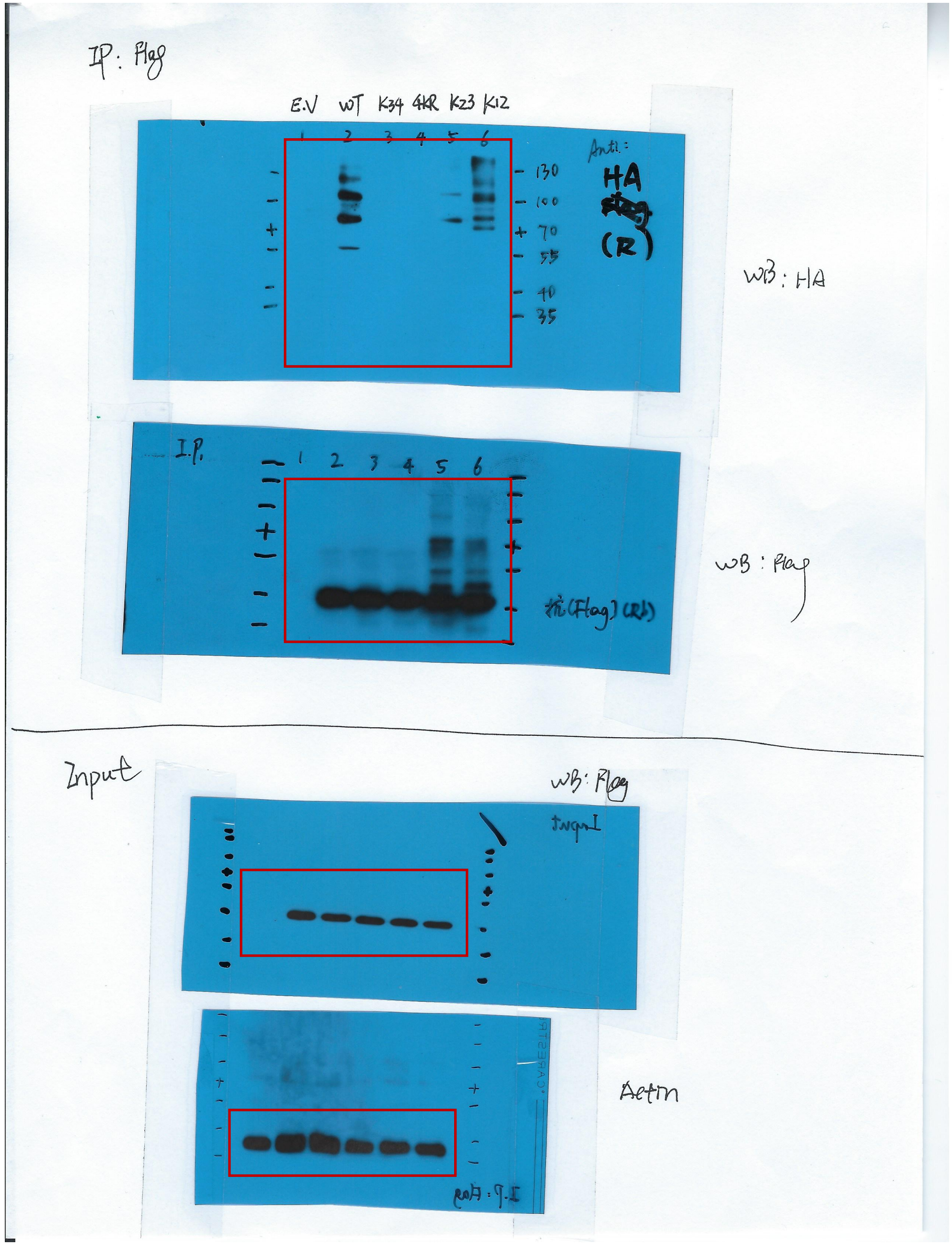
IP: Flag
E.V WT K34 4KR K23 K12
1 2 3 4 5 6
130
100
70
55
40
35
HA
(R)
WB: HA
I.P.
1 2 3 4 5 6
WB: Flag
Input
WB: Flag
Actin

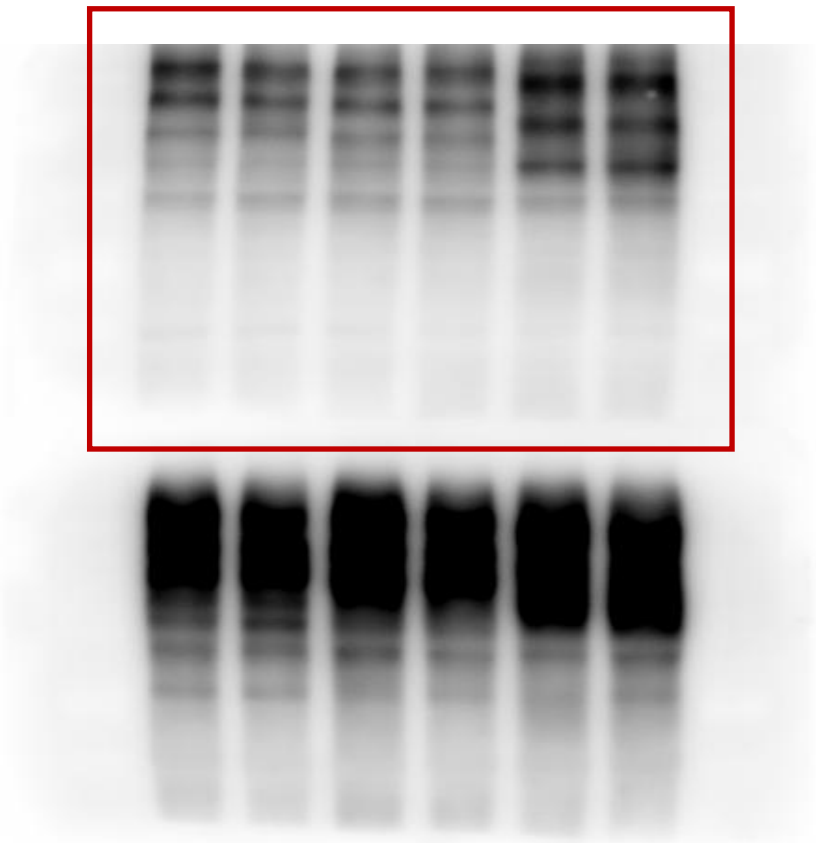

Figure 2D

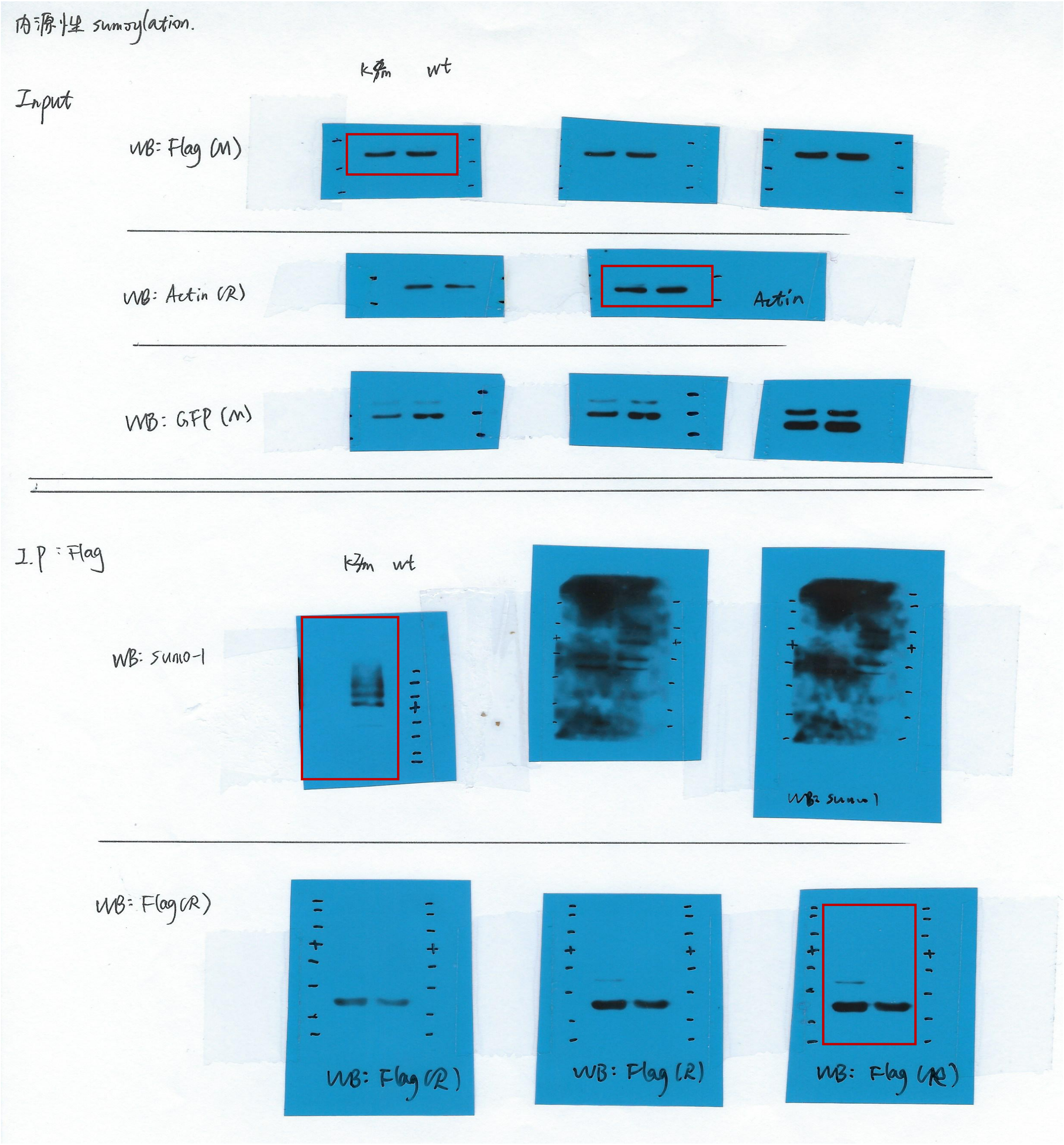

内源性 sumoylation.
K3m wt
Input
WB: Flag (M)
WB: Actin (R)
Actin
WB: GFP (M)
I.P : Flag
WB: sumo-1
WB: sumo1
WB: Flag (R)

Figure 3A

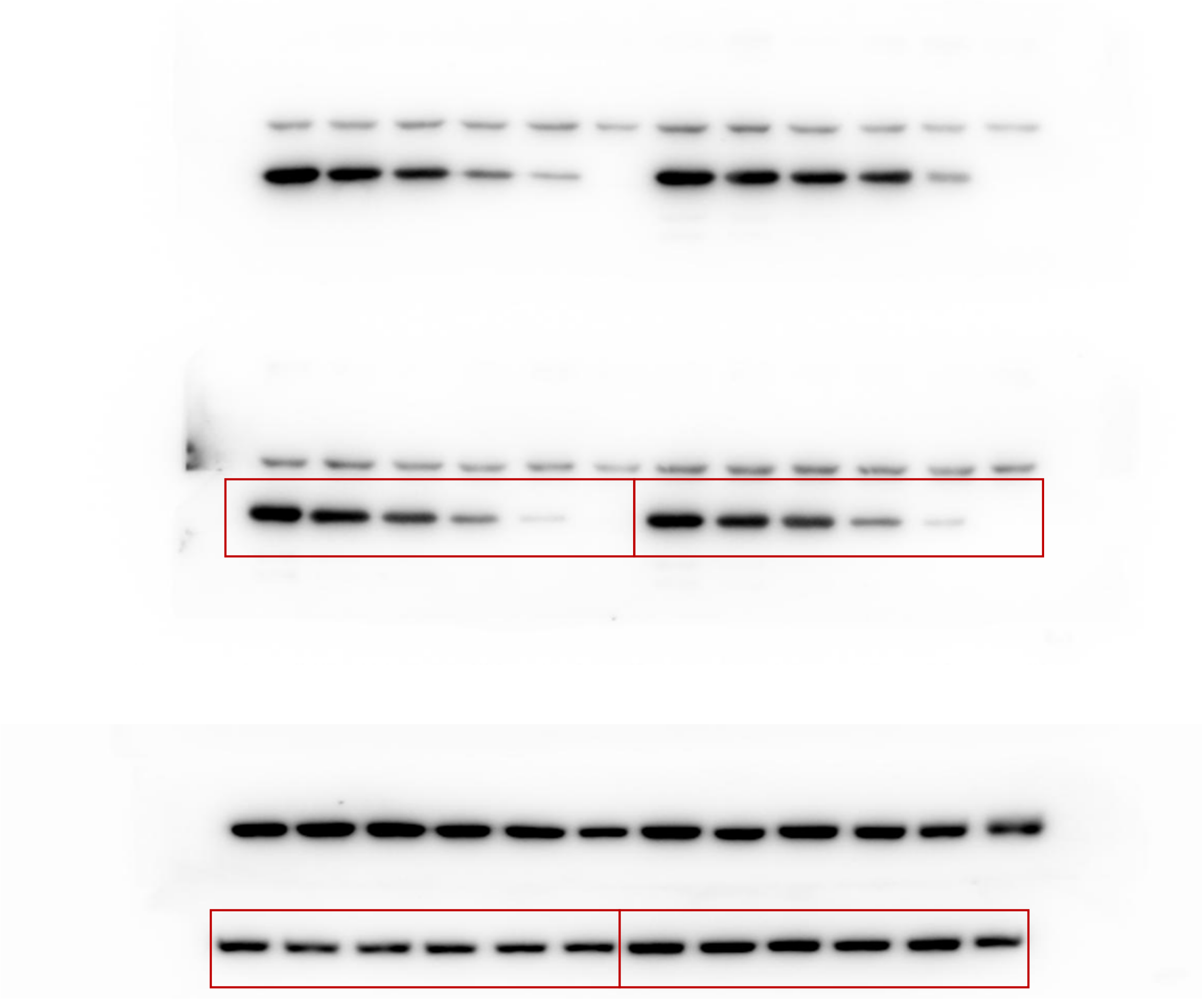

Figure 3B

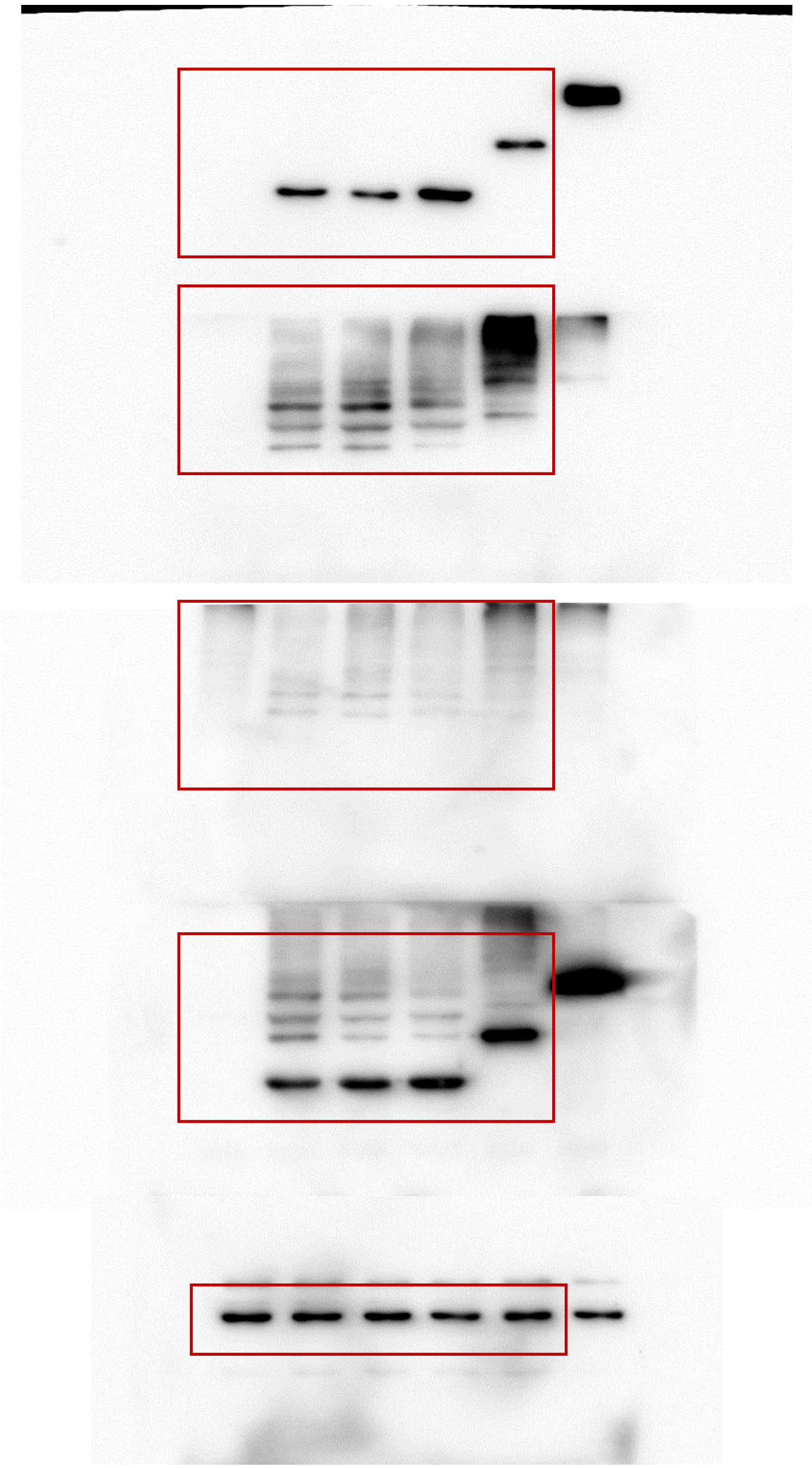

Figure 3C

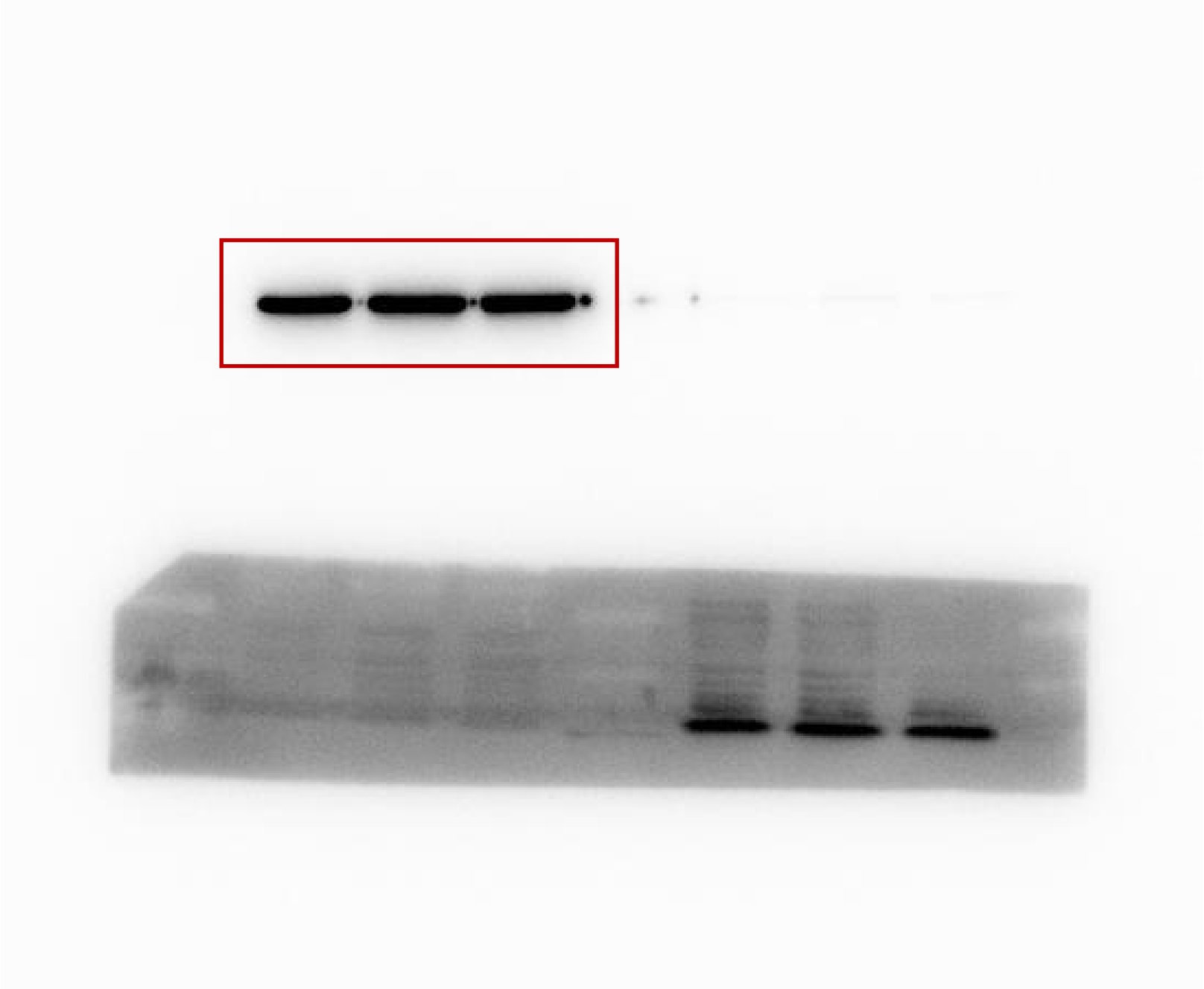

Figure 4A

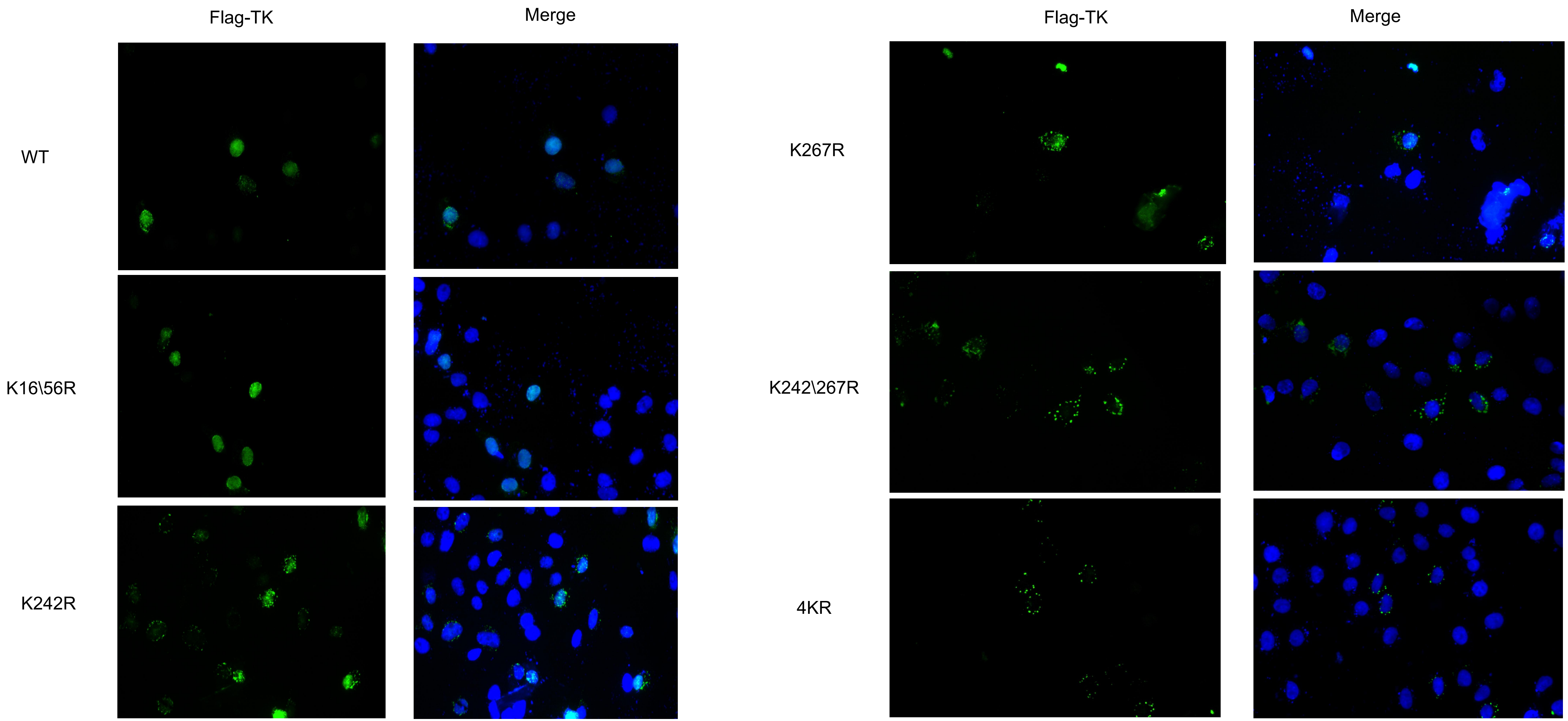
Flag-TK
Merge
WT
K16\56R
K242R
Flag-TK
Merge
K267R
K242\267R
4KR

Figure 4B

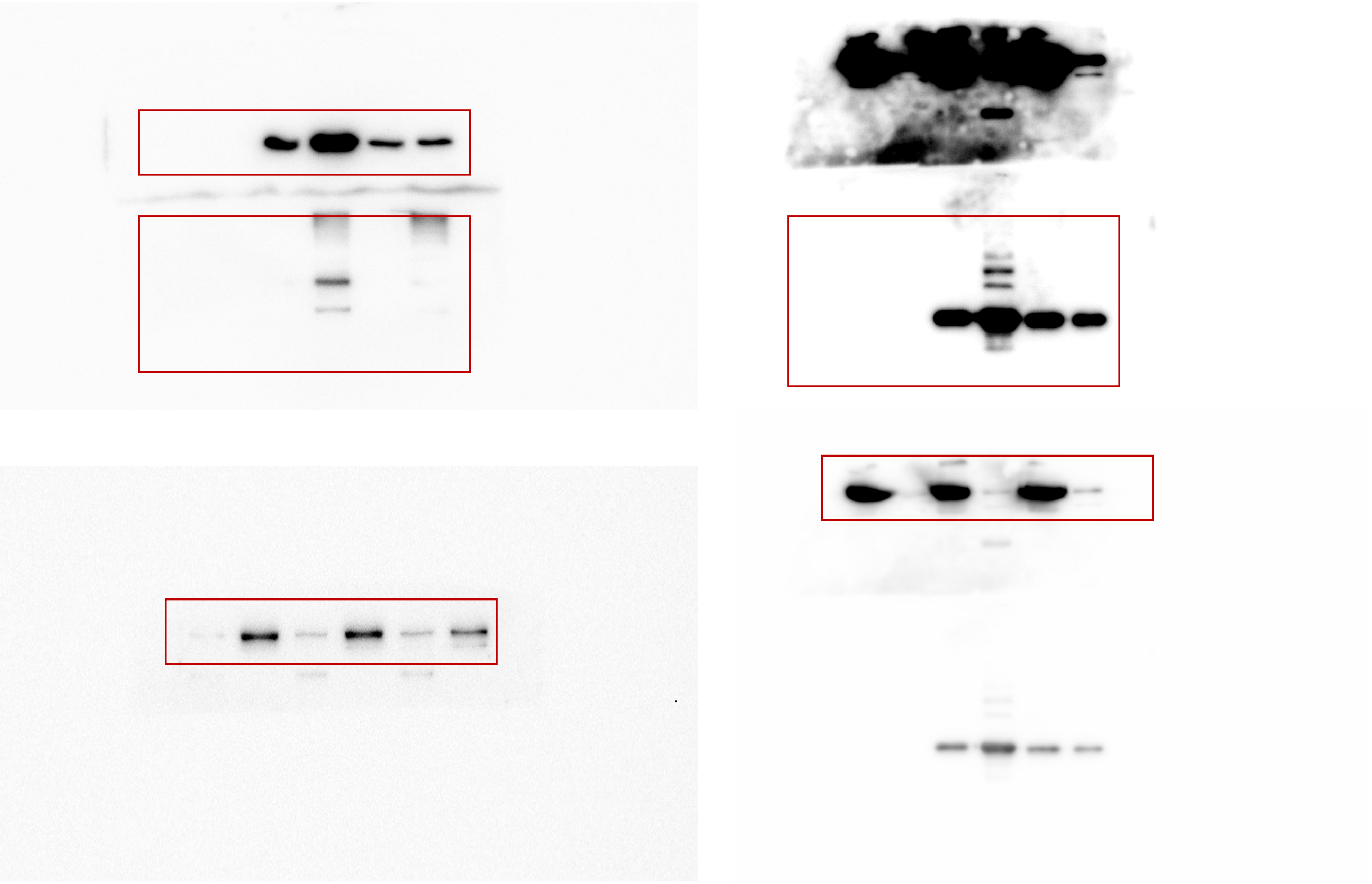

Figure 5A

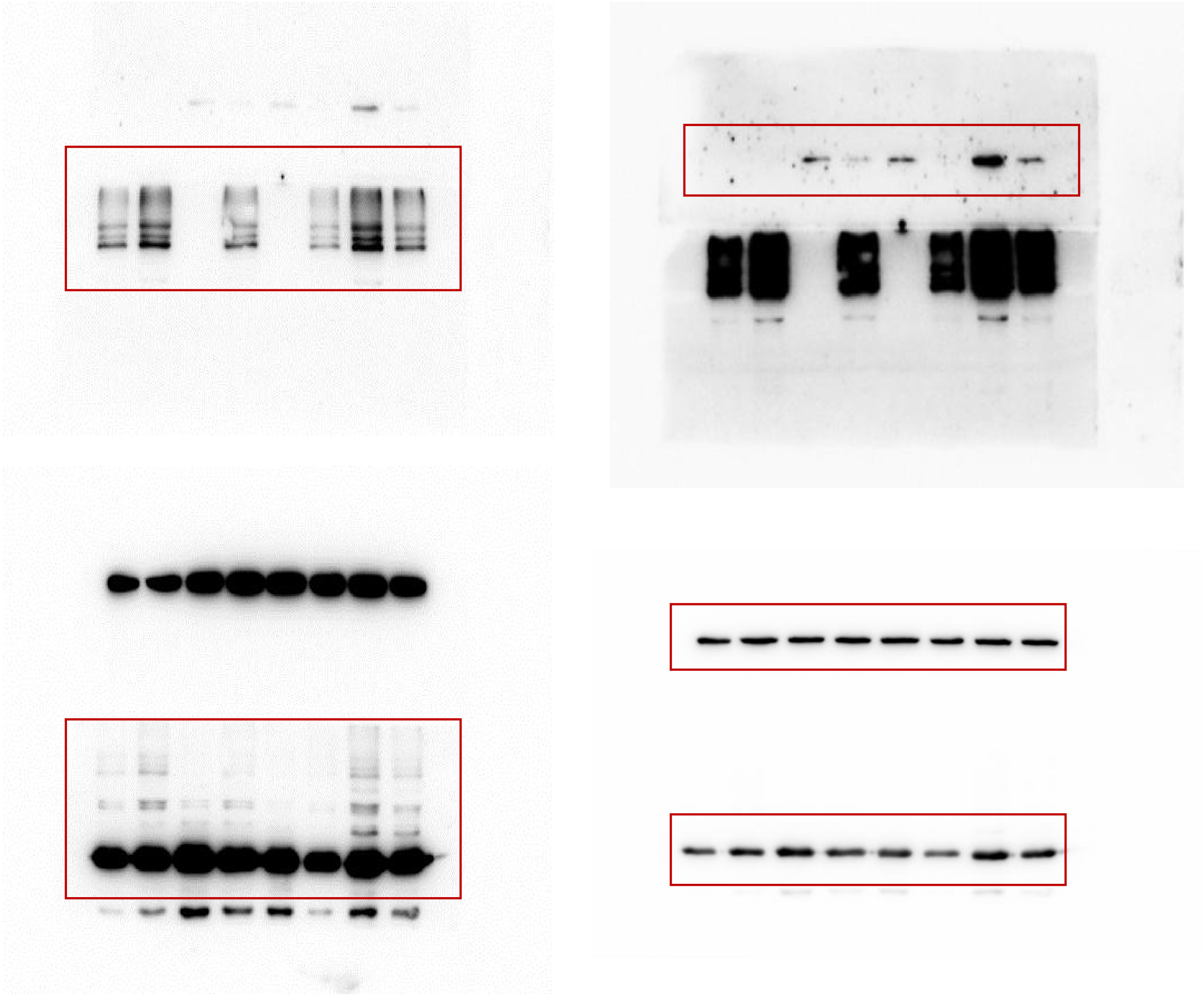

Figure 5B

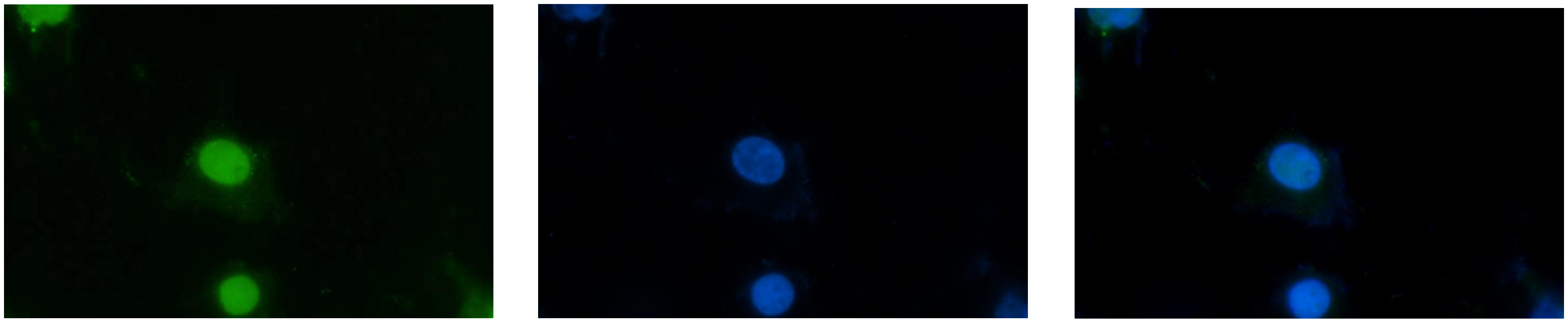

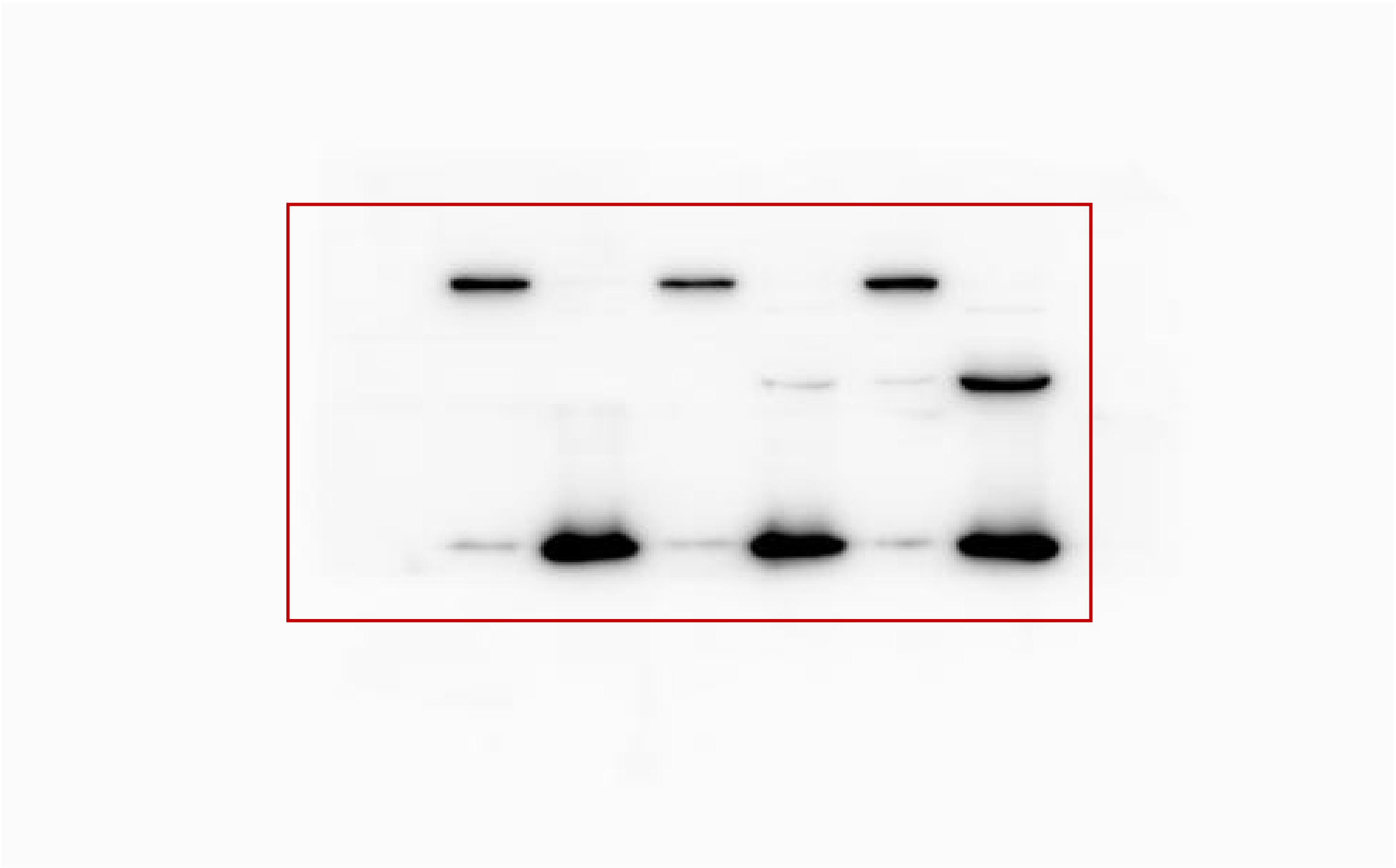

Figure 5C

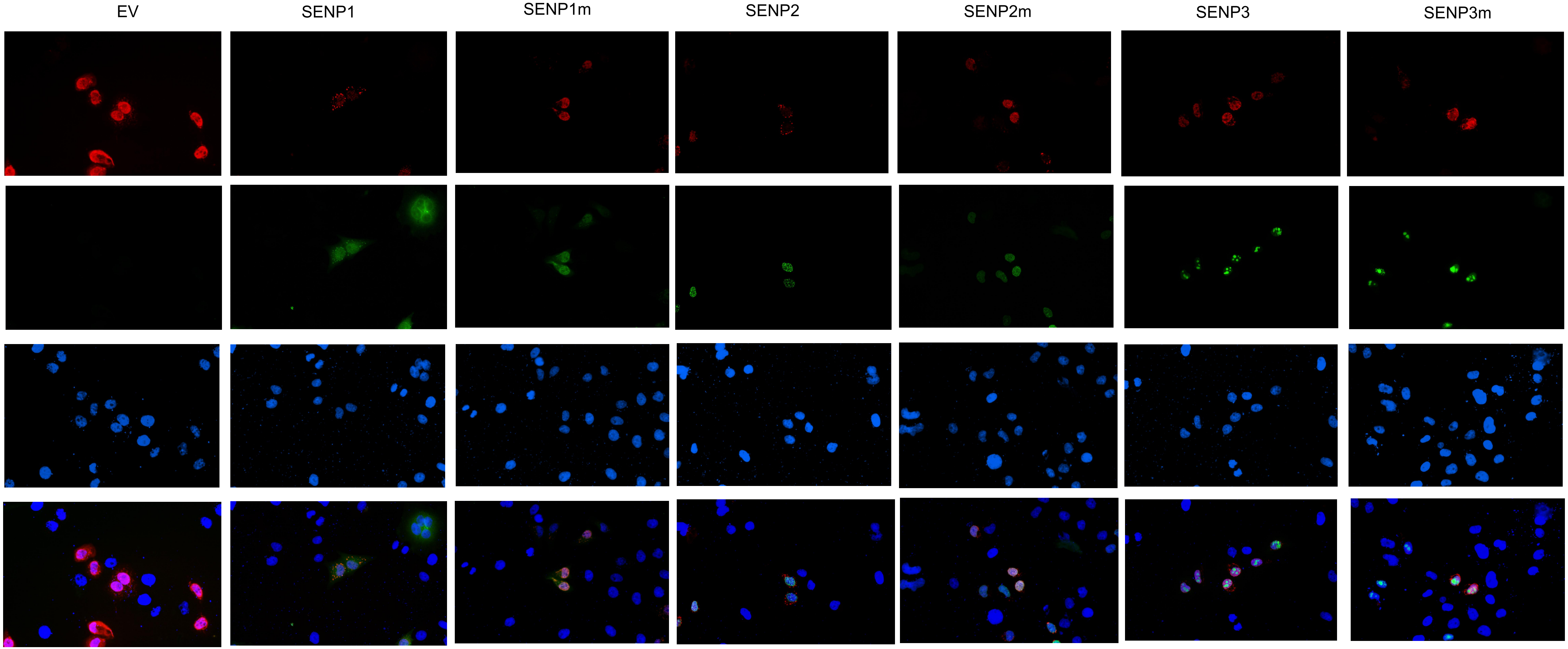

Figure 6B

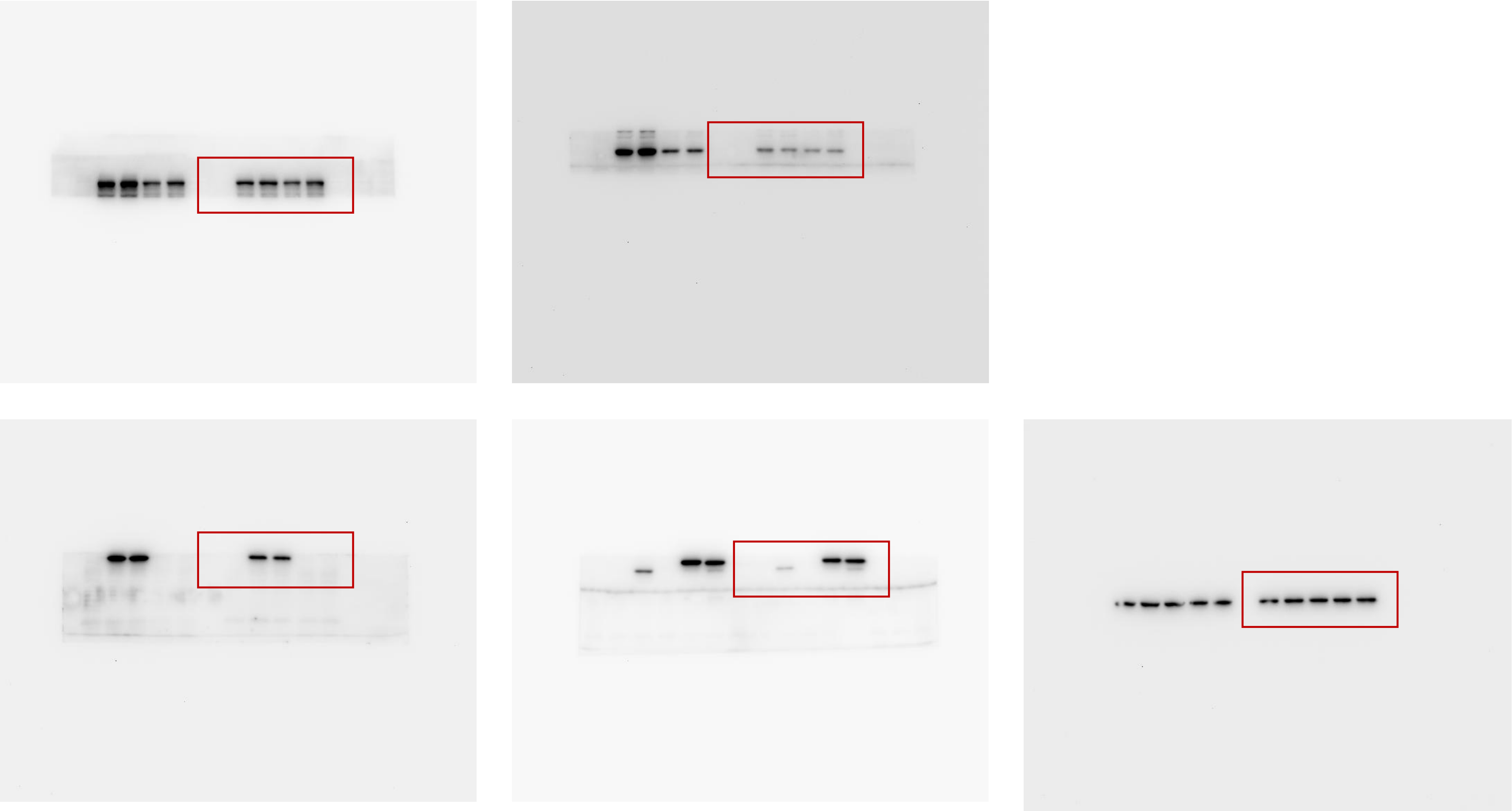

Figure 6C

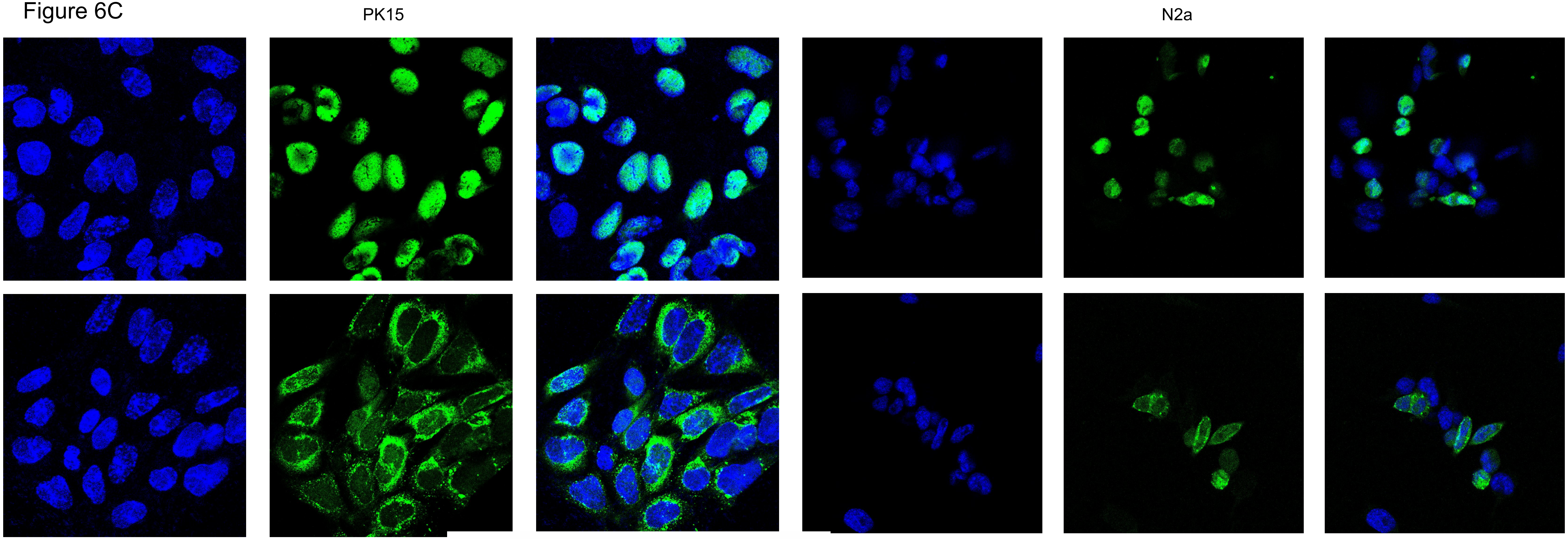

Figure 6D

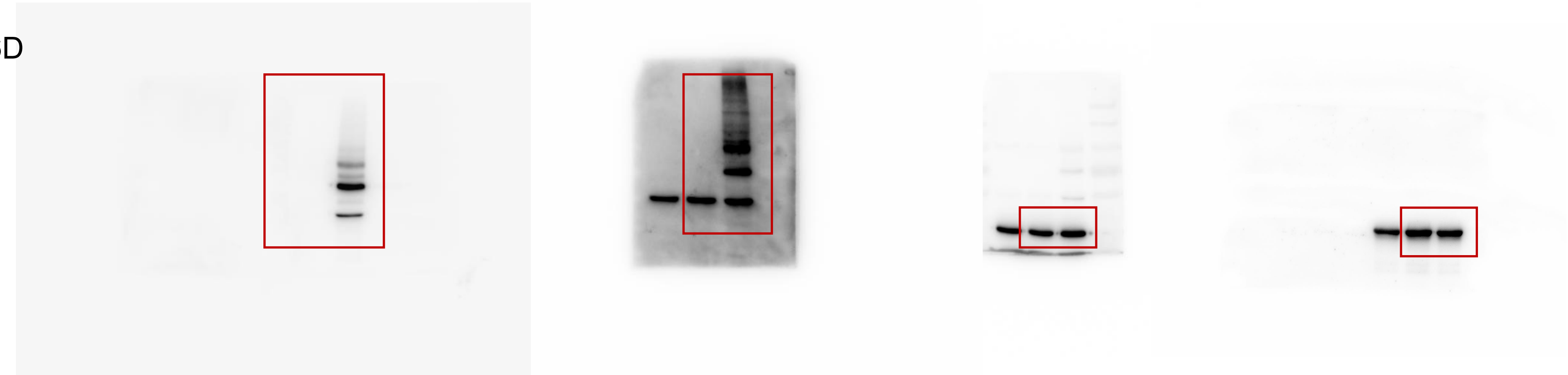

Figure 6E

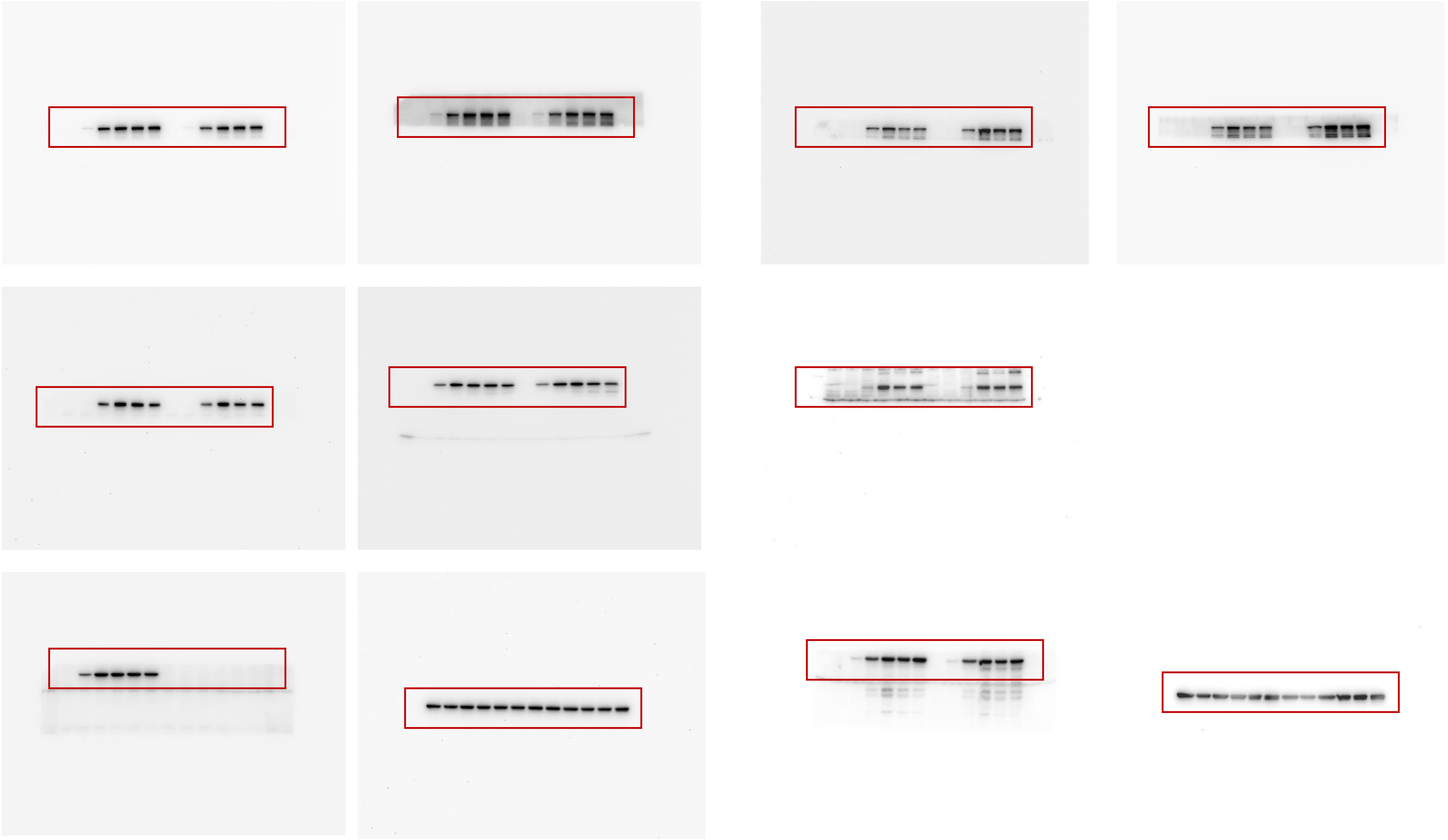

Figure 6F

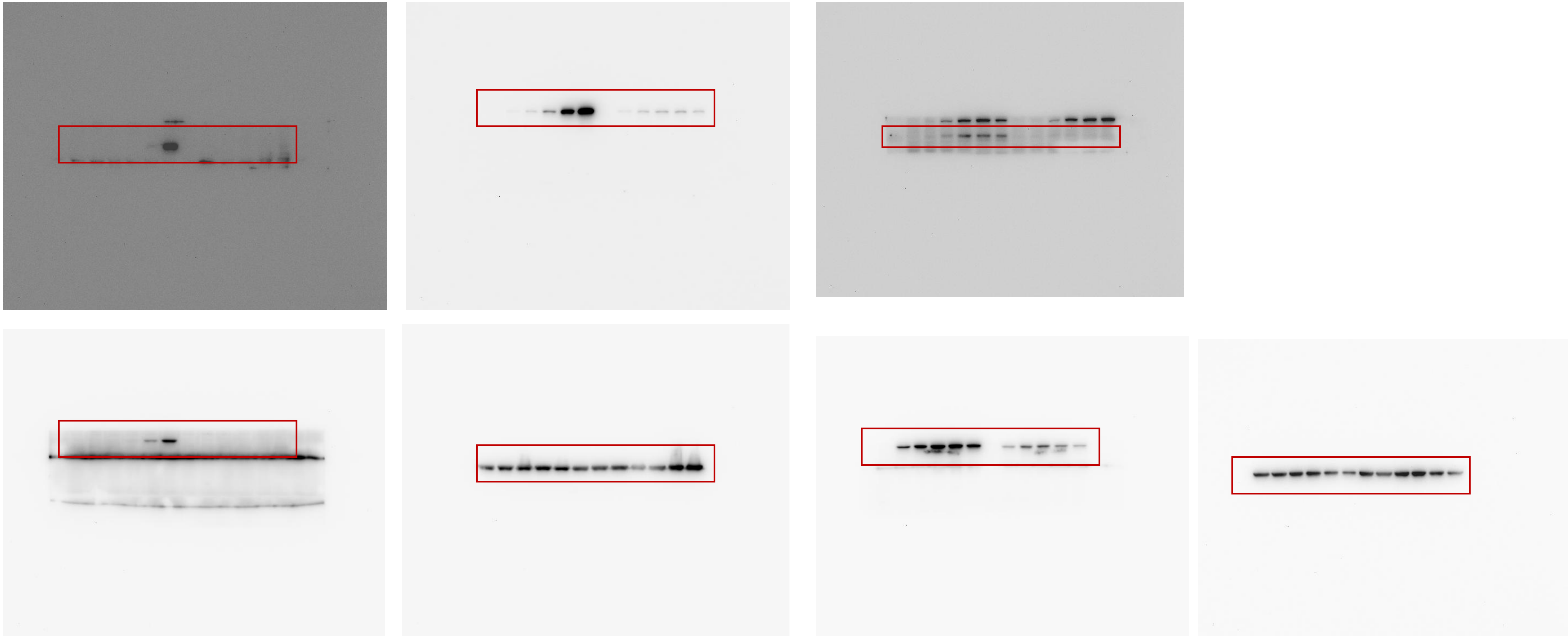

Figure 7D

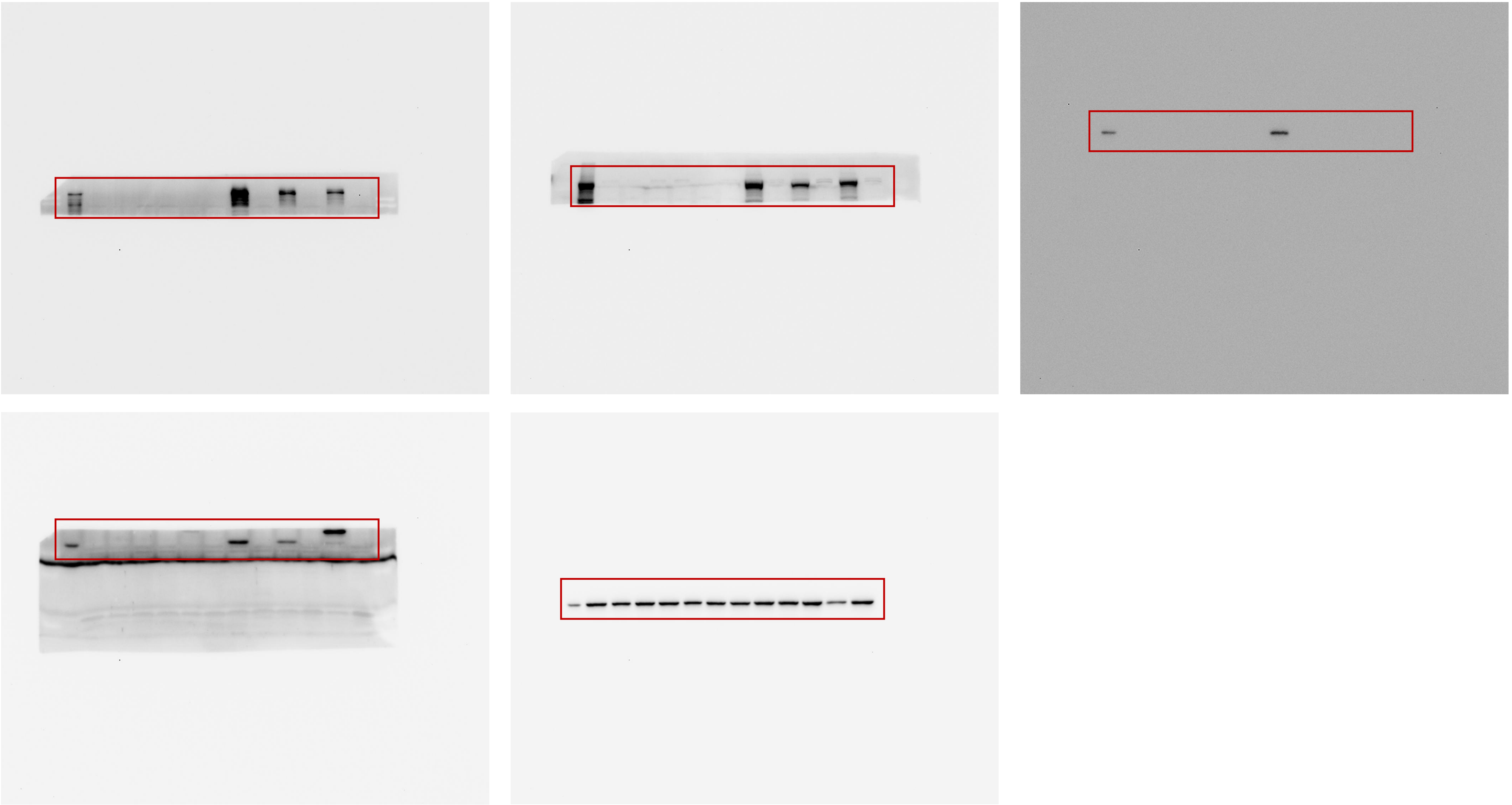

Figure S2F

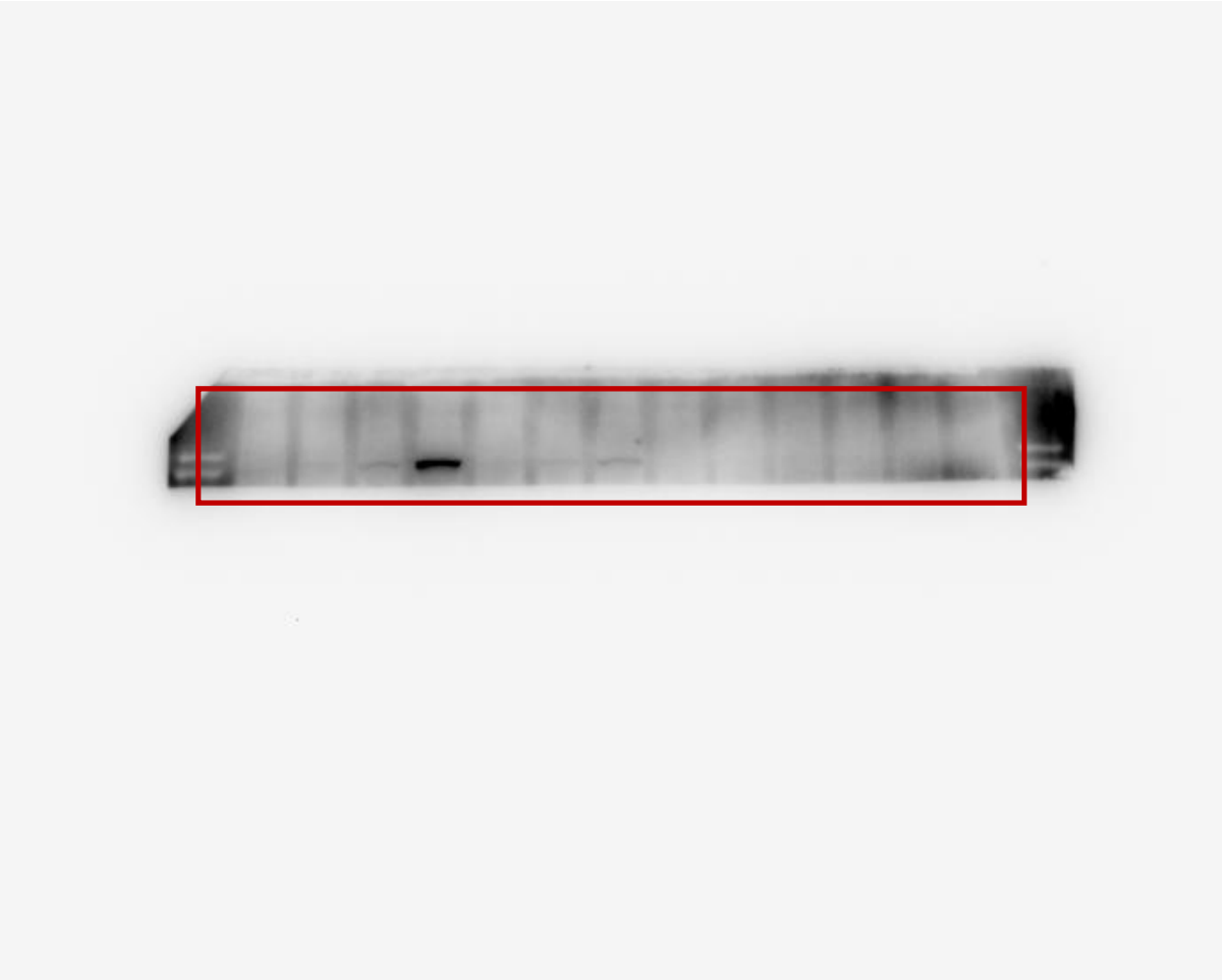

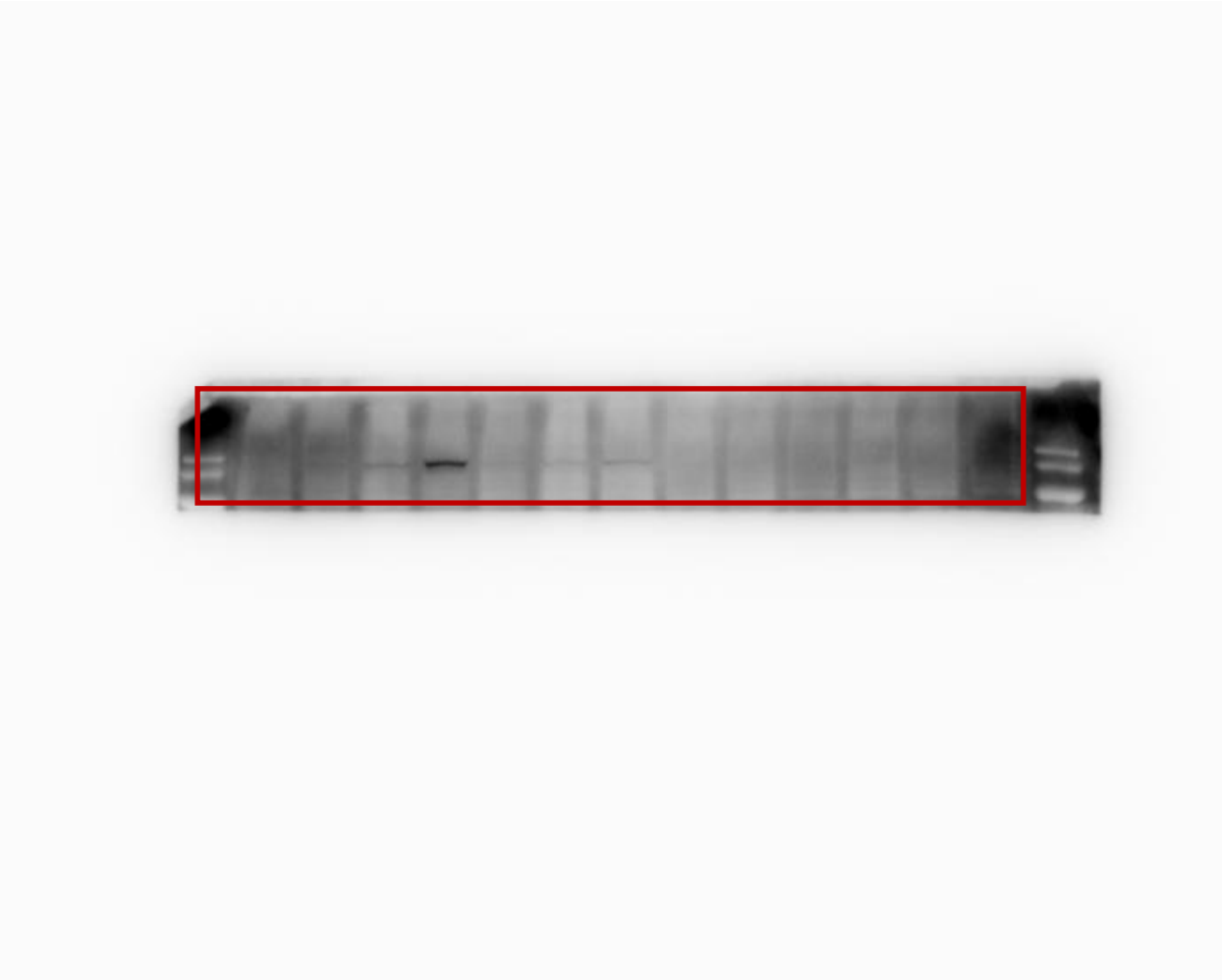

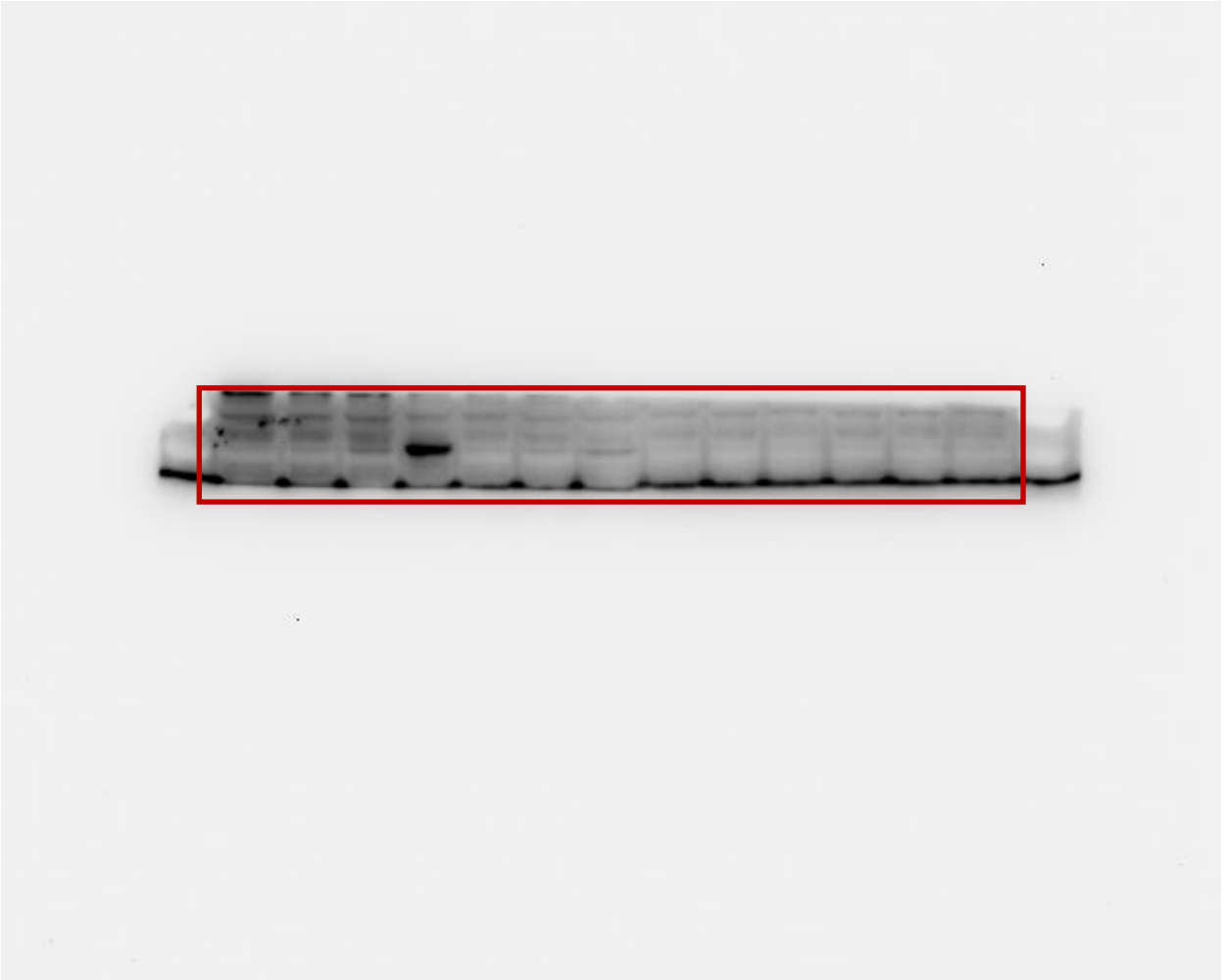

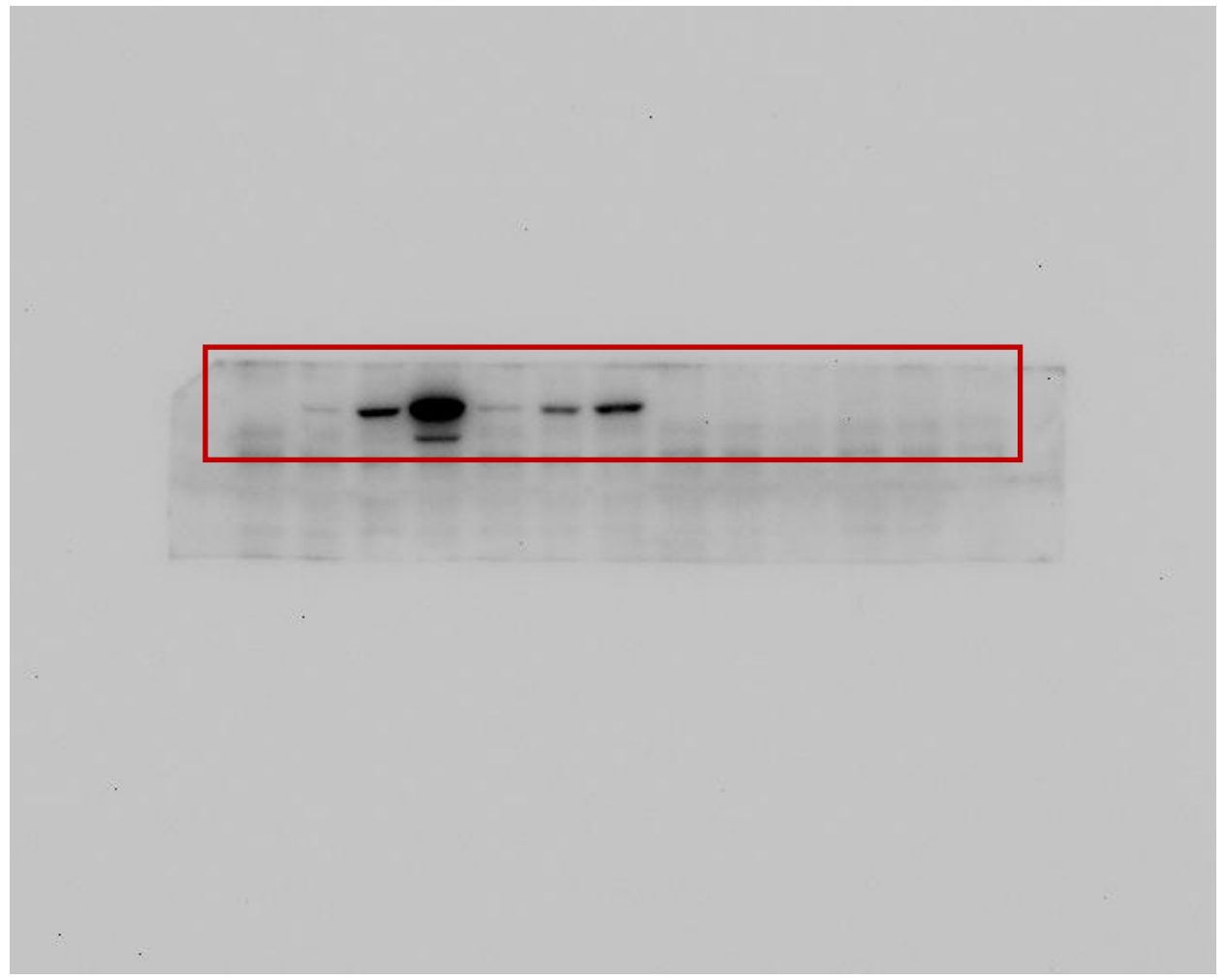

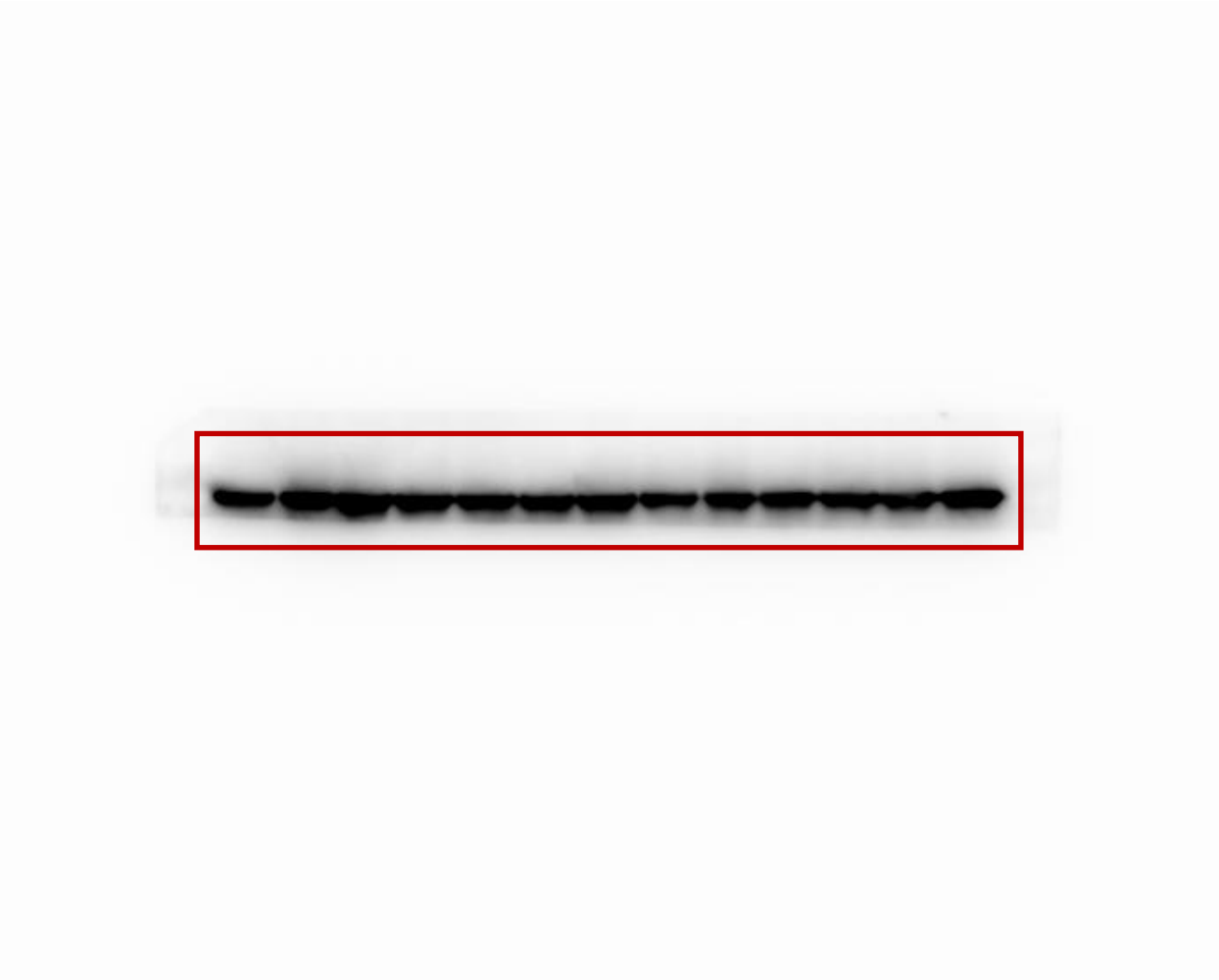

Figure S3F

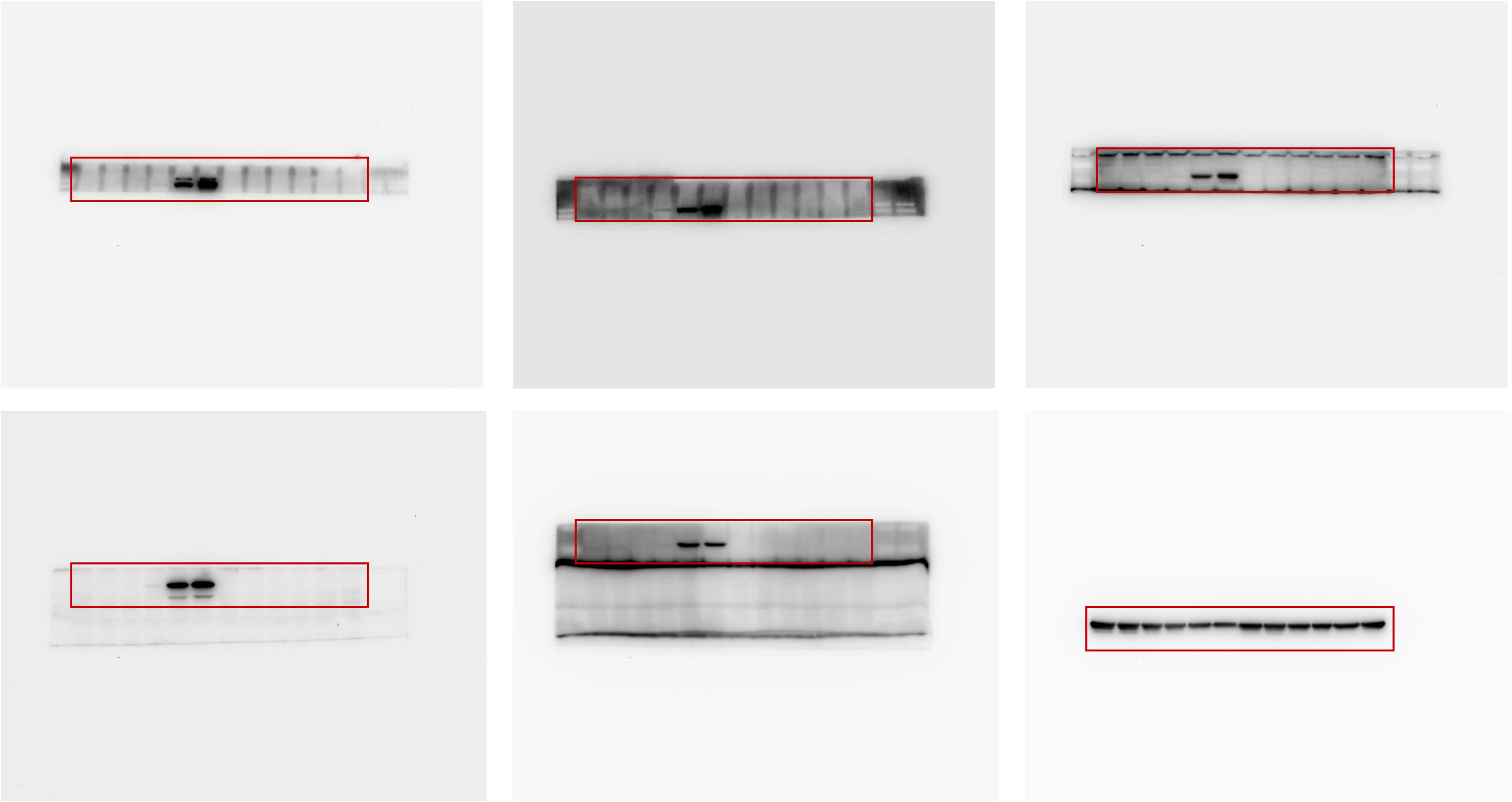

Figure S4B

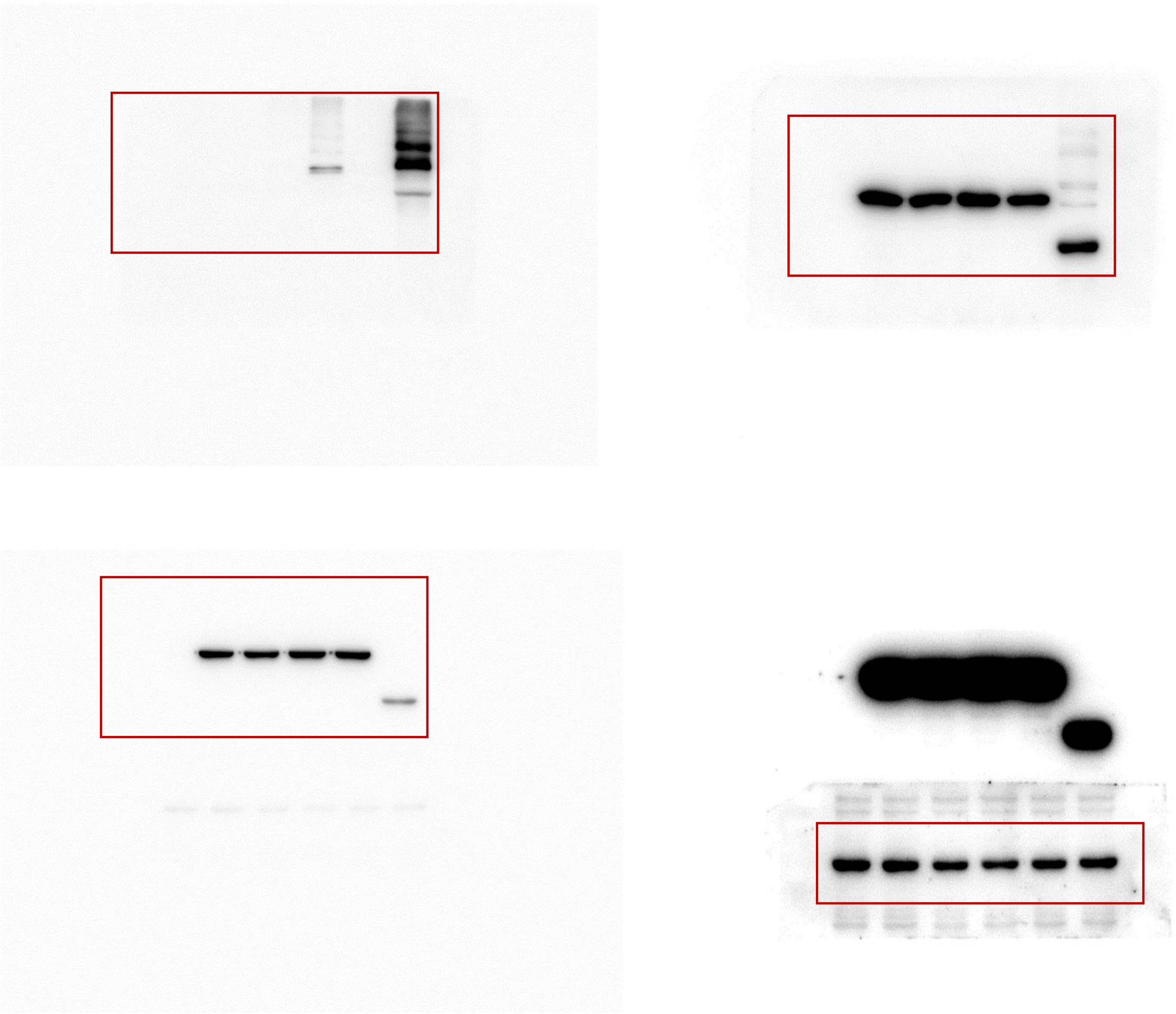

Supplement: Supplementary file 1 [file ppat.1014653.s005.pdf]
