# Supplementary material for: SUMOylation of pseudorabies virus thymidine kinase regulates its nuclear localization and facilitates neuronal replication and reactivation
Source: PLoS Pathog. 2026 Oct 5;22(10):e1014653. doi: 10.1371/journal.ppat.1014653 (PMC13649132; doi:10.1371/journal.ppat.1014653)

Response to specific comments:

Reviewer #1:

Chen and coauthors identified the critical biological function of viral TK SUMOylation in PRV infection. SUMOylation is essential for TK trafficking to nuclear and subsequent viral replication and reactivation. The modification site(s) on TK were determined and validated. SUMOylation-resistant mutant viruses lost the ability of nuclear localization and efficient viral replication in cells and in vivo. The finding is innovative and provides a valuable reference for designing antiviral defense. The study is within the scope of this journal. However, it could be strengthened by addressing the following concerns.

Major Issues: Key Experiments Required for Acceptance:

1. TK is the substrate of all SUMO1,2,3, and SUMO3 shows a much stronger TK SUMOylation in Figure 1D. So, why choose SUMO1, but not SUMO3 for the following studies, and antiviral target?

We agree that TK can be modified by multiple SUMO paralogs, and our data in indeed showed that SUMO3 produced a relatively stronger SUMO-associated signal compared with SUMO1 under overexpression conditions. However, we chose to focus primarily on SUMO1 in the subsequent mechanistic studies for several reasons.

First, SUMO1-mediated conjugation has been more extensively characterized in the context of viral protein regulation and typically produces more stable and experimentally tractable SUMOylation patterns, whereas SUMO2/3 modifications are often more dynamic and associated with poly-SUMO chain formation. Second, our primary goal in this study was to investigate whether SUMOylation itself regulates TK localization and neuronal replication phenotypes, rather than to define SUMO paralog specificity in detail. Therefore, while systematic analysis of individual SUMO paralogs during PRV infection would be valuable for future investigation, the current study was designed primarily to establish the biological significance of TK SUMOylation itself. we have added the above explanation to the Discussion section of the revised manuscript（lines 433-441）.

2. Would knocking down SUMO1, 2, and 3 abolish TK SUMOylation? And prevent viral replication or reactivation? And the following question is what's the status of TK and mutations SUMOylation by endogenous SUMO1, 2, and 3?

Regarding the reviewer's suggestion on SUMO1/2/3 knockdown experiments, we agree that this is an important question. However, global depletion of SUMO paralogs may broadly affect multiple host cellular pathways and viral replication processes beyond TK SUMOylation itself, making interpretation of the resulting phenotypes more complicated. In addition, functional redundancy among SUMO paralogs may limit the interpretability of single-paralog depletion experiments.

To address the reviewer’s concern about “the status of TK and mutations SUMOylation by endogenous SUMO”, we have now included additional experiments in the TK and mutations SUMOylation by endogenous SUMO1 (revised Figure 1C and 2D) as well as the endogenous SUMOylation of TK occurs during PRV infection (revised Figure 1D and 6D) in the revised manuscript.

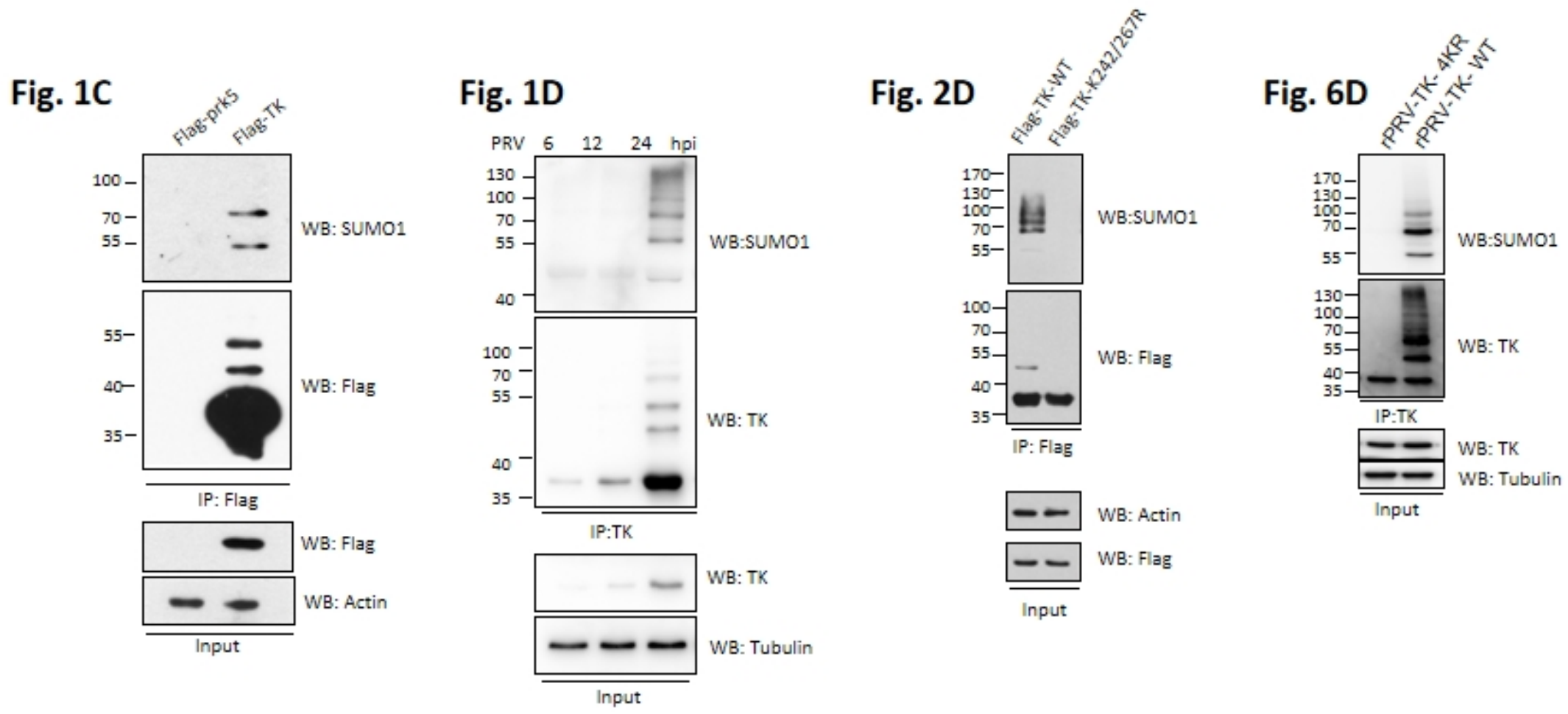

3. SUMO-conjugating enzyme Ubc9 was co-transfected with SUMO1 in the assay, but SUMOylation is still occurring without external Ubc9 expression (Fig.1D), so why using the articulated conditions by introducing overexpressed Ubc9? Please include conditions without overexpressing Ubc9.

We agree that endogenous Ubc9 is sufficient to mediate detectable TK SUMOylation, as SUMO-associated signals were still observed in the absence of exogenous Ubc9 expression in original Figure 1D. The primary purpose of co-transfecting Ubc9 in our assays was to enhance the efficiency and detectability of SUMO conjugation, which is commonly employed in SUMOylation studies because SUMO-modified species are often low-abundance and dynamically regulated.

To address the reviewer’s concern, we have now included additional experimental conditions without Ubc9 overexpression in the TK and mutations SUMOylation by endogenous SUMO1(revised Figure 1C and 2D) as well as the endogenous SUMOylation of TK occurs during PRV infection (revised Figure 1D and 6D) in the revised manuscript (as shown above) and clarified this rationale in the Results and Methods sections.

4. In the PK15 cell, SUMOylation has no effect on viral replication (Fig. 7D, 7E), while N2a has a much stronger phenotype. So, would it be valuable to show results in N2a cells rather than PK15 cells? Since there is no phenotype (viral growth curve) in PK15, how to interpret or explain the results in Figure 6? Would that be the same result when using N2a? Please include the data using N2a cells in Figure 6.

We thank the reviewer for this insightful comment. We agree that the biological phenotype associated with TK SUMOylation is much more pronounced in neuronal N2a cells than in

epithelial PK15 cells. In the present study, PK15 cells were initially selected for the localization experiments because of their high transfection efficiency and suitability for imaging-based analyses.

In the original manuscript, Figure 6 was designed to investigate the effect of SUMOylation on TK subcellular localization, whereas Figure 7 evaluated the functional consequences of this modification on viral replication. Therefore, we do not believe that the observations in Figures 6 and 7 are contradictory.Specifically, our data indicate that SUMOylation promotes nuclear localization of TK in PK15 cells, yet disruption of TK SUMOylation does not significantly affect viral replication in these cells. A possible explanation is that TK primarily facilitates viral DNA synthesis by providing phosphorylated thymidine nucleotides. In rapidly dividing epithelial cells such as PK15 cells, abundant endogenous dTTP pools may compensate for the reduced nuclear accumulation of SUMOylation-deficient TK, thereby masking its impact on viral replication. In contrast, neuronal cells such as N2a possess limited endogenous nucleotide resources and rely more heavily on viral TK activity. Under these conditions, impaired nuclear localization of SUMOylation-deficient TK is more likely to result in defective viral DNA replication, leading to the pronounced replication and reactivation phenotypes observed in N2a cells.To clarify this point, we have added the above explanation to the Discussion section of the revised manuscript（lines 503-512）.

We agree that validating the localization phenotype in N2a cells would further strengthen the mechanistic link between TK SUMOylation and neuronal replication. However, because N2a cells exhibit relatively low transfection efficiency, it is technically difficult to reproduce the SENPs plasmid-based localization experiments shown in original Figure 6. To address the reviewer’s concern, we instead examined TK localization in the context of viral infection. In the revised Figure 6C, we performed immunofluorescence analysis of N2a cells infected with the recombinant viruses rPRV-TK-WT and rPRV-TK-4KR. Consistent with the results obtained in PK15 cells, mutation of the TK SUMOylation sites markedly reduced nuclear accumulation of TK in N2a cells, providing direct evidence that TK SUMOylation also contributes to its nuclear localization in neuronal cells.

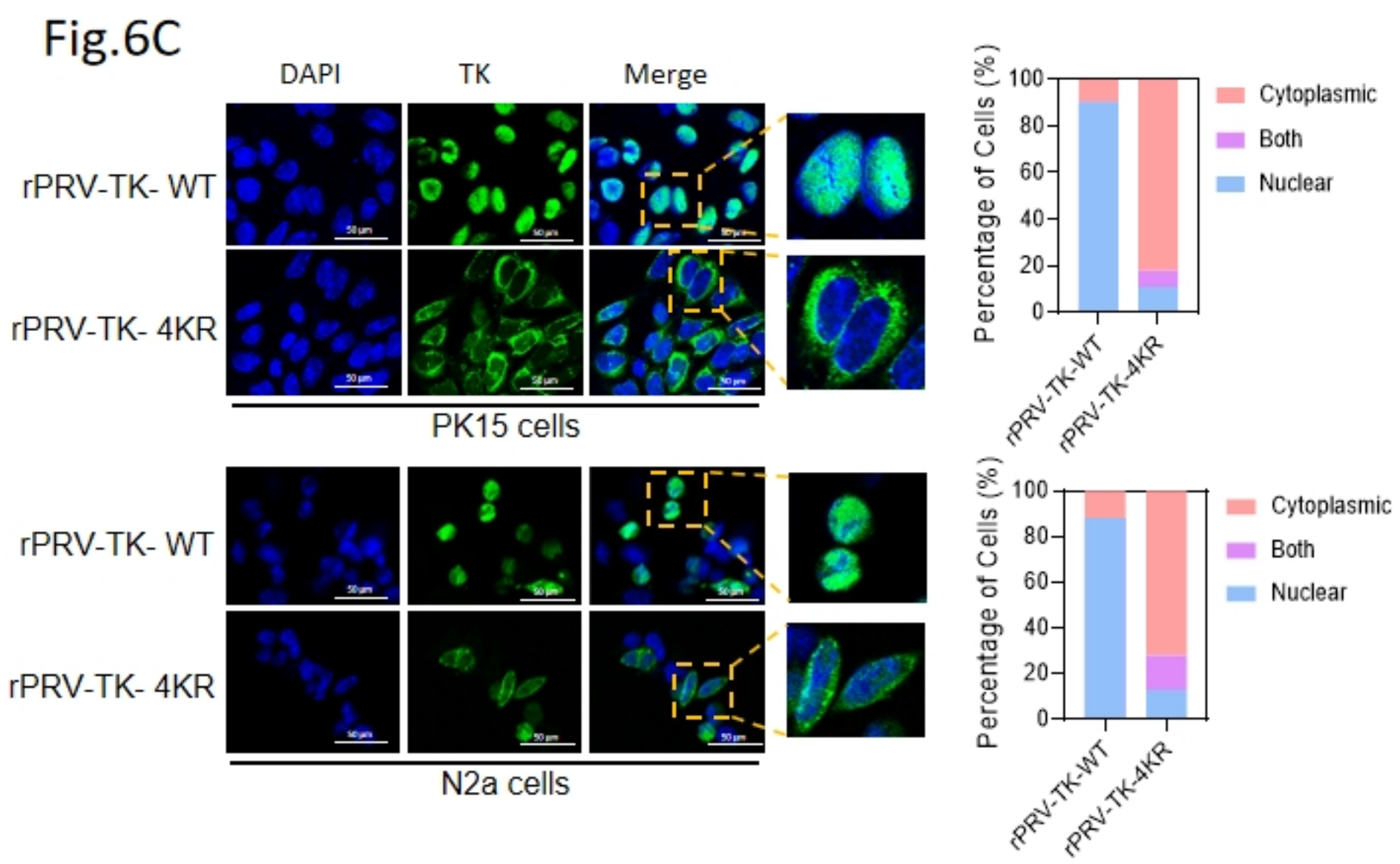

5. The section on HSV-1 is a bit out of context in this manuscript, as the main focus is PRV-related studies. It is interesting that HSV-1 TK is the substrate of SUMO1, not SUMO2. However, it is better to show this section at the end with more evidence of TK SUMOylation in various alpha-herpes. Alternatively, include more results of recombinant HSV-1 in cells and in vivo studies in the following section.

We agree that the HSV-1 data are not central to the main conclusions of this study. As the current work does not include recombinant HSV-1 mutants or corresponding *in vitro* and *in vivo* functional analyses, the available data are insufficient to support an extensive discussion of the biological significance of HSV-1 TK SUMOylation.

Following the reviewer's suggestion, we have removed the HSV-1 TK SUMOylation data from the main figures and transferred them to the Supplementary Materials (Figure S4). In additon, we also performed a multiple sequence alignment of TK proteins from several representative alphaherpesviruses, including PRV, HSV-1, bovine herpesvirus 1 (BHV-1), equine herpesvirus 1 (EHV-1), and Marek's disease virus (MDV). These new data have been included as Supplementary Fig. S4 and are discussed in the revised Discussion (lines 453－468).

Revised Fig. S4

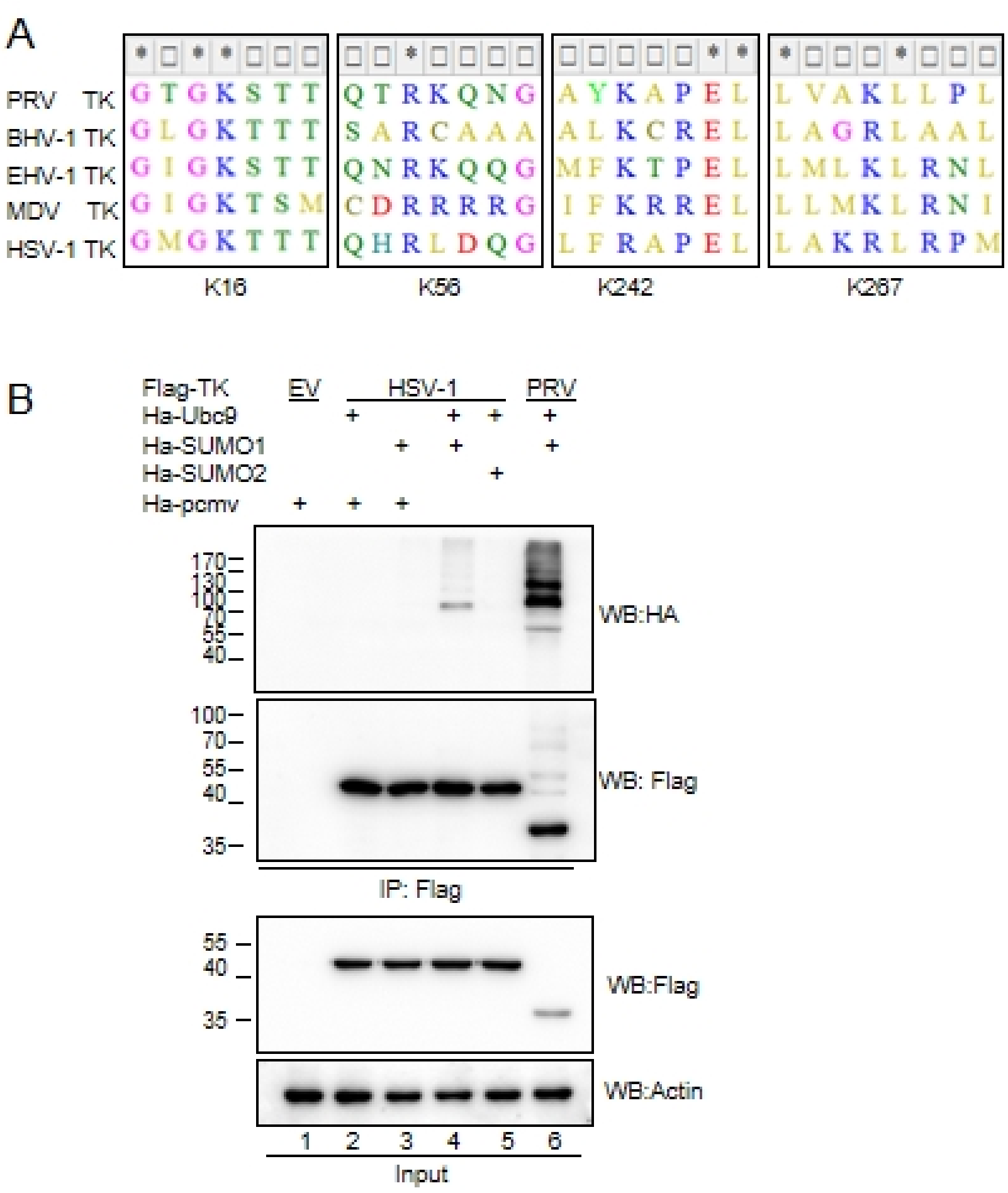

The sequence comparison showed that the predicted SUMOylation-associated lysine residues are partially conserved among alphaherpesviruses. Specifically, K16 is completely conserved, whereas K242 is replaced by arginine only in HSV-1, and K267 is replaced only in BHV-1. In contrast, K56 is considerably less conserved and is retained only in PRV and EHV-1. Among the viruses analyzed, PRV and EHV-1 exhibit the highest conservation of all four lysine residues.

These findings suggest that the regulatory mechanism mediated by TK SUMOylation may be at least partially conserved during alphaherpesvirus evolution, while species-specific divergence may also exist. Notably, the reduced SUMOylation observed for HSV-1 TK (Fig. S4B) may be related to the replacement of K56 and K242 by arginine, although this possibility requires further experimental validation.

Minor Issues: Editorial and Data Presentation Modifications

1. When performing the SUMOylation assay, what's the rationale for only screening Us1, Us3, UL13, TK, or UL48? What's the selection criteria? By Mass spectrometry using infected cells? Structure analysis? Please include more details and clarification.

In this study, we did not intend to perform a systematic screen of all PRV-encoded proteins for SUMOylation. Instead, we adopted a candidate-based strategy, focusing on several PRV proteins with well-characterized enzymatic or regulatory activities that are known to play essential roles during viral replication. Specifically, Us3 and UL13 are viral serine/threonine protein kinases, TK is the viral thymidine kinase, UL48 functions as a major tegument transactivator, and Us1 (ICP22 homolog) is an important viral regulatory protein involved in viral gene expression. Because post-translational modifications frequently regulate the activity, localization, or substrate specificity of enzymes and regulatory proteins, these candidates were selected as an initial group for SUMOylation analysis.

We agree that PRV encodes many additional proteins that may also undergo SUMOylation. Our study was not designed as a comprehensive SUMO-proteome analysis, and therefore we cannot exclude the possibility that other PRV proteins are SUMO substrates. We have now clarified the selection rationale in the Results (lines 133-136) and Discussion (lines 552-556) sections and acknowledge this as a limitation of the present study.

2. Figure 1C, 1D, please indicate the size of the protein band through out the figures. Some WB blots show, but some do not.

As suggested, the sizes of the protein bands through out the figures have been labeled.

3. Please align the symbols + and – correctly in each WB panel, e.g. Fig1D.

We have carefully checked the Figures and correced this issue.

4. Figure 2D, please provide the structure model ID used for PyMOL modeling.

The ribbon structure of TK was predicted by AlphaFold (model ID: AF-G3G8S8-F1), and the model ID has been shown in Figure legends.

5. To examine the biological role of TK SUMOylation, it flows better with the protein stability, localization and deSUMOylating affecting localization.

We thank the reviewer for this insightful suggestion, and we have rearranged the narrative order of the manuscript as suggestion.

6. Figure 7C, how many images were used for quantification? Please show the standard deviation.

We performed the quantitative analysis of the immunofluorescence images (revised Fig. 4A and 6C). The subcellular localisation of TK was quantified by counting at least 50 cells from three independent experiments. The graphs present the percentage of cells exhibiting nuclear, cytoplasmic, or both nuclear and cytoplasmic localisation.

7. The Figure was not correctly called out in the manuscript. Please correct the Figure numbers. Figure 9 is missing.

Thank you for pointing this out. We have corrected the Figure numbering and ensured that all figures are appropriately cited in the revised manuscript.

8. The H&E staining of infected tissue was not sufficient to show the viral pathogenesis. Please provide staining evidence of viral proteins (TK is preferred), cell markers and SUMO1.

During revision, we repeated the mouse infection experiments using an increased number of animals (n = 6 per group) and attempted to detect viral antigen in brain and lung tissues by immunohistochemistry. Although we attempted to detect TK antigen in brain sections, the anti-TK antibody that performs well in Western blotting and immunofluorescence did not generate sufficiently robust and reproducible staining in paraffin-embedded tissues (see the following figures), preventing reliable evaluation of viral distribution. Therefore, we did not include these data in the revised manuscript.

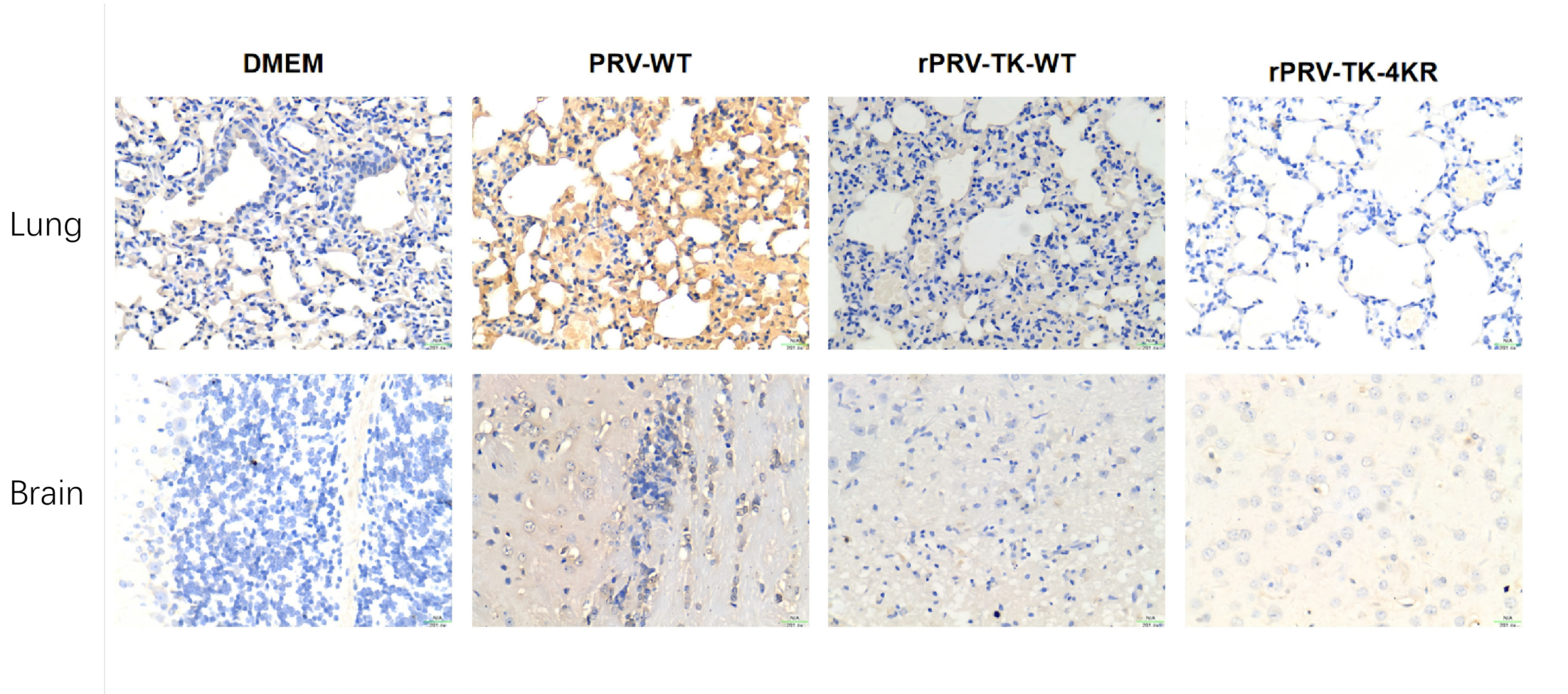

Nevertheless, the repeated animal experiments consistently demonstrated that the rPRV-TK-4KR mutant exhibited markedly reduced mortality, no weight loss, significantly lower viral DNA copy numbers in brain tissues, and substantially attenuated histopathological lesions compared with rPRV-TK-WT. Together, these data support the conclusion that loss of TK SUMOylation markedly impairs viral infection within the CNS.

We agree that distinguishing whether this attenuation primarily reflects impaired neuroinvasion or reduced replication after CNS entry is an important mechanistic question. We have therefore added this point to the Discussion (lines 567-573) and consider it an important direction for future investigation.

9. Lack of experimental details. Details of CRISPR/Cas9 system, concentrations of primary and secondary antibody in the methods, should be provided for data reproducibility. What's the qPCR condition? Performed on which instrument?
We have added the experimental details in the revised Materials and Methods section. Specifically, we have provided additional information regarding the CRISPR/Cas9 system, including the vector system and procedures used for viral genome editing. The concentrations and incubation conditions of primary and secondary antibodies used for immunoblotting and immunofluorescence assays have also been added. In addition, we have included the details of the quantitative PCR assays, including the qPCR reaction conditions, cycling parameters, and the instrument used for detection. These revisions have been incorporated into the revised manuscript.

10. Animal experimental procedures, for example, how tissue was processed for viral titration and other analysis?

We have revised the Materials and Methods section to provide additional details regarding animal sample processing. Specifically, we have added information on tissue collection, homogenization, clarification, and subsequent processing for viral load determination and histopathological analysis.

11. Please indicate the number of mice used in each group in the figure legends and in the methods. How is the number of animals determined?

We have revised the Materials and Methods section and corresponding figure legends to indicate the number of animals used in each experimental group. The number of mice per group (n=6) was determined based on preliminary experiments and previous PRV infection studies, while considering statistical reliability and minimizing the number of animals used in accordance with animal welfare principles.

12. Line 515, add a space before Briefly.

Corrected.

Reviewer #2:

This manuscript identifies SUMOylation of PRV thymidine kinase (TK) at K242/K267 and claims that this modification promotes TK nuclear localization, neuronal replication, reactivation from latency, and in vivo virulence. While the study is extensive and addresses an interesting topic, several critical issues undermine the current conclusions.

Major Issues: Key Experiments Required for Acceptance:

1. All SUMOylation experiments were performed using overexpression systems. The authors should provide evidence that endogenous SUMOylation of TK and its mutants occurs in the context of genuine viral infection. It remains unclear whether the observed SUMOylation is a physiologically relevant event during PRV replication rather than an artifact of transient transfection.

We agree that demonstrating endogenous SUMOylation of TK during authentic viral infection is essential for establishing the physiological relevance of our findings. In fact, our original manuscript already provided evidence that Flag-TK interacts with endogenous SUMO1 (Fig. 1C), indicating that TK can be modified by endogenous SUMO machinery without exogenous SUMO1 overexpression.

To further address the reviewer's concern, we have included additional experiments in the revised manuscript. Specifically, we examined the association of Flag-TK-WT and the Flag-TK-K242/267R mutant with endogenous SUMO1 (revised Fig. 2D), confirming that mutation of the major SUMOylation sites markedly reduces endogenous SUMO1 modification. Furthermore, we have added data demonstrating endogenous SUMO1 modification of TK in the context of PRV and recombinant virus infection (revised Figs. 1D and 6D). These results demonstrate that TK undergoes endogenous SUMOylation during authentic viral infection and that the identified SUMOylation sites contribute substantially to this modification.

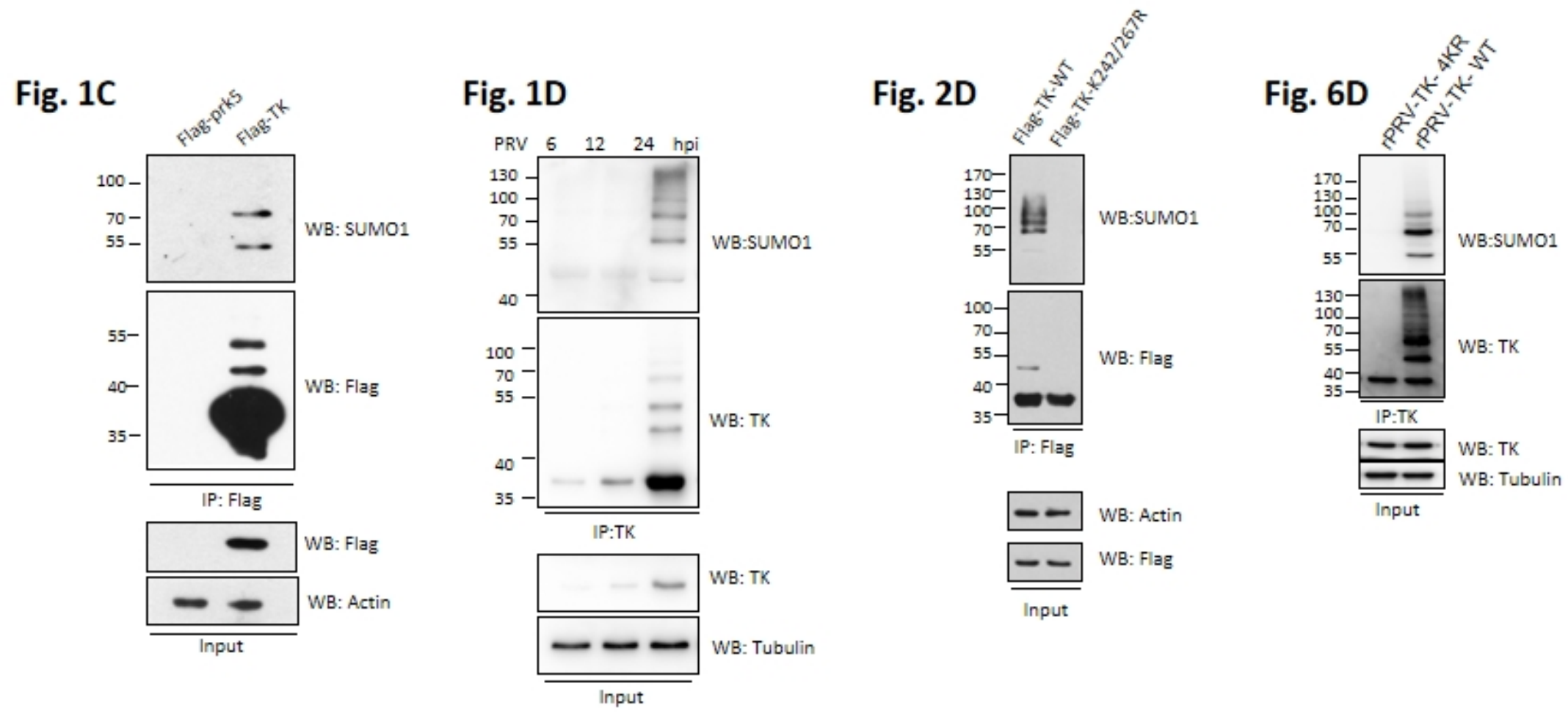

2.The in vivo virulence conclusion is confounded by disruption of the Us3 Locus. The recombinant viruses (rPRV-TK-WT and rPRV-TK-4KR) were constructed by inserting the TK expression cassette into the Us3 gene, thereby inactivating Us3, a well established neurovirulence factor. Because rPRV-TK-WT (Us3 null) already exhibits marked attenuation compared to parental PRV WT, the additional loss of TK SUMOylation in rPRV-TK-4KR cannot be ascribed to reduced pathogenicity. The mouse experiment lacks the essential control – direct comparison between rPRV-TK-WT and rPRV-TK-4KR to support the claim that TK SUMOylation per se contributes to virulence.

We thank the reviewer for this insightful comment. Actually, we initially attempted to introduce TK-WT or TK-4KR directly into the endogenous TK locus using our CRISPR/Cas9-based strategy. However, despite multiple independent attempts, we were unable to recover the desired recombinant viruses. Although the exact reason remains unclear, this may reflect the relatively low efficiency of homologous recombination at the endogenous TK locus or technical limitations associated with repeated editing of this genomic region.

Therefore, we adopted the Us3 locus as the insertion site, which has been routinely used in our previous studies because disruption of Us3 does not significantly affect viral replication in cultured cells. We fully acknowledge, however, that Us3 is an established neurovirulence factor and the contribution of Us3 disruption should be experimentally addressed.

To directly address this concern, we repeated the in vivo infection experiments by increasing the number of mice to six animals per group and including two additional control viruses, PRV-US3-KO and PRV-TK/US3-KO, in the revised study (revised Figure 8). The results show that Mice infected with PRV-WT developed severe pruritus and scratching behavior by 4 days post-infection (dpi), and all animals succumbed to infection within 6 dpi (Fig. 8A). Consistent with the established role of Us3 in PRV neurovirulence, PRV-US3-KO-infected mice exhibited similar clinical signs but showed delayed disease progression, with 100% mortality occurring by 7 dpi.

Importantly, despite sharing the same Us3-null background, rPRV-TK-WT and rPRV-TK-4KR displayed markedly different phenotypes in vivo. While both PRV-WT and PRV-US3-KO caused 100% mortality, rPRV-TK-WT showed a reduced mortality rate (80%) and delayed disease progression. More importantly, rPRV-TK-4KR exhibited complete survival (0% mortality), representing a significant attenuation compared with rPRV-TK-WT. This difference was further supported by body weight changes (revised Fig. 8B) , viral genome loads in tissues (revised Fig. 8C) , and histopathological analyses (revised Fig. 8D) , all of which consistently demonstrated reduced pathogenicity of the TK SUMOylation-deficient virus. Therefore, although Us3 deletion contributes to the overall attenuation of the recombinant virus backbone, the direct comparison between rPRV-TK-WT and rPRV-TK-4KR indicates that loss of TK SUMOylation confers an additional and significant reduction in viral virulence.

We also noted that rPRV-TK-WT and PRV-US3-KO were not completely identical in terms of mortality and body weight loss. One possible explanation is that insertion of the TK expression cassette into the Us3 locus may alter the timing and/or expression level of TK during the viral replication cycle compared with the native genomic context. We have included this point in the revised Results (lines 378-382) and Discussion (lines 536-551) as a potential limitation and interpretation of the recombinant virus phenotype.

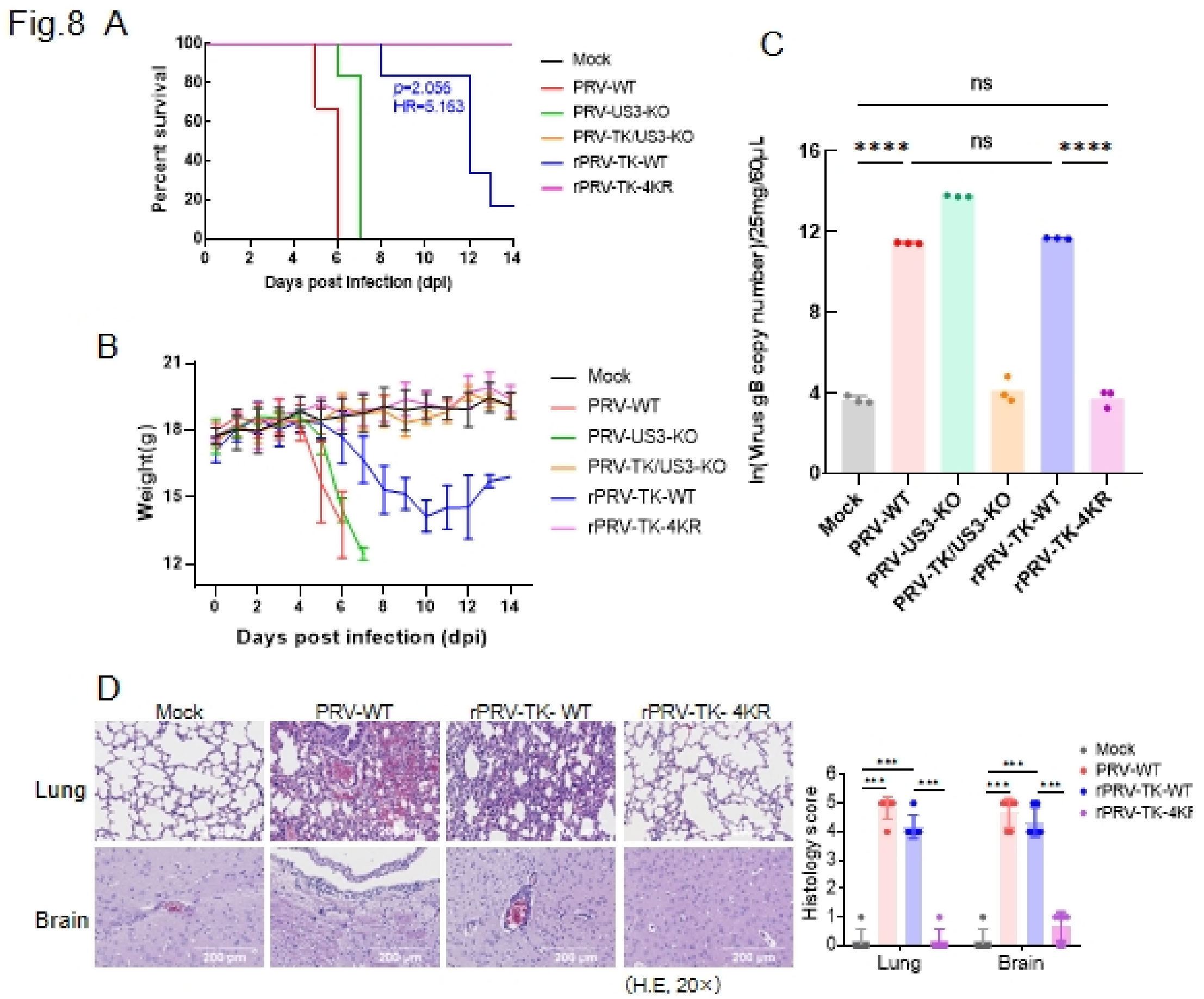

3. The proposed reactivation defect may simply reflect a general replication defect of the SUMOylation-deficient virus. The authors report that rPRV-TK-4KR fails to reactivate in

N2a cells, but they do not compare the latent viral genome loads established prior to reactivation between rPRV-TK-WT and rPRV-TK-4KR. Without evidence that equal amounts of latent genomes are present before the temperature shift, the observed failure to reactivate could be entirely explained by reduced replication capacity rather than a specific block in the latency-reactivation switch.

We thank the reviewer for this insightful comment. We fully agree that comparable latent viral genome loads prior to reactivation are essential for distinguishing a specific defect in viral reactivation from a general replication defect.

In fact, this control had already been included in our study but was not sufficiently emphasized in the original manuscript (original Fig.8). In the revised manuscript, we have repeated these experiments again and reorganized the Results section and explicitly present the data obtained at both the latency-induction stage (42 °C for 3 days) and the reactivation stage (37 °C for 5 days) in revised Figure 7.

Importantly, after 3 days of incubation at 42 °C, all viruses, including PRV-WT, PRV-TK-KO, PRV-US3-KO, PRV-TK/US3-KO, rPRV-TK-WT, and rPRV-TK-4KR, exhibited similarly low levels of extracellular viral genome copies (gB), IE180 and LAT transcripts, intracellular viral DNA, viral protein expression, and infectious virus production. None of these parameters differed significantly from the mock control, whereas all were markedly lower than those of the lytic infection control maintained at 37 °C (revised Fig. 7A–E, left or upper panels). These results demonstrate that all viruses established comparable latent-like infections under the latency-inducing conditions.

Following the temperature shift to 37 °C, rPRV-TK-WT efficiently resumed viral genome replication, viral gene expression, and infectious virus production, whereas rPRV-TK-4KR remained at baseline levels across all assays (revised Fig. 7A–E, right or down panels). Thus, the impaired phenotype of rPRV-TK-4KR is not attributable to defective establishment of latency or reduced viral genome abundance before reactivation. We also have clarified this point in the revised Discussion section (lines 521–535).

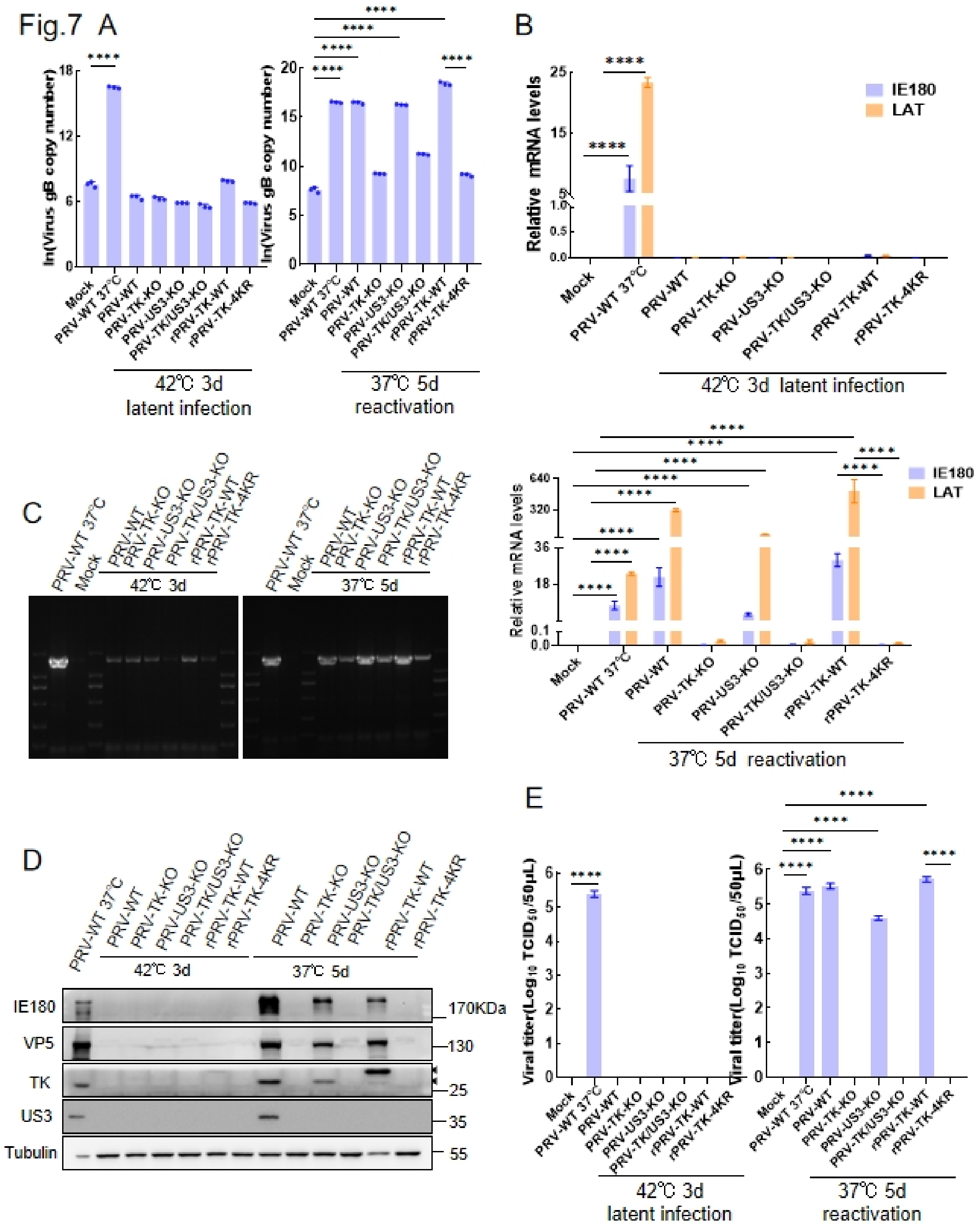

Minor Issues: Editorial and Data Presentation Modifications

1. PRV is a large DNA virus encoding 70–100 proteins. Why were only Us1, Us3, UL13, TK, and UL48 examined for SUMOylation? It is not clear whether other PRV-encoded proteins are also SUMOylated. The authors should at least discuss this limitation.

In this study, we did not perform a systematic screen of all PRV-encoded proteins for SUMOylation. Instead, we adopted a candidate-based strategy, focusing on several PRV proteins with well-characterized enzymatic or regulatory activities that are known to play essential roles during viral replication. Specifically, Us3 and UL13 are viral serine/threonine protein kinases, TK is the viral thymidine kinase, UL48 functions as a major tegument

transactivator, and Us1 (ICP22 homolog) is an important viral regulatory protein involved in viral gene expression. Because post-translational modifications frequently regulate the activity, localization, or substrate specificity of enzymes and regulatory proteins, these candidates were selected as an initial group for SUMOylation analysis.

We agree that PRV encodes many additional proteins that may also undergo SUMOylation. Our study was not designed as a comprehensive SUMO-proteome analysis, and therefore we cannot exclude the possibility that other PRV proteins are SUMO substrates. We have now clarified the selection rationale in the Results (lines 133-136) and Discussion (lines 552-556) sections and acknowledge this as a limitation of the present study.

2. In the co immunoprecipitation experiments, the input blots do not show HA-SUMO1 expression. Please include HA SUMO1 input controls to demonstrate equal expression of the SUMO construct across samples.

As suggested, the HA-SUMO1 expression has been shown as the input control.

3. All SUMOylation evidence is derived from overexpression of Flag-TK and HA-SUMO. The authors need to determine whether endogenous SUMOylation of TK occurs during actual PRV infection.

To address the reviewer's concern, we have now included additional experiments in the TK and mutations SUMOylation by endogenous SUMO1 (revised Figure 1C and 2D) as well as the endogenous SUMOylation of TK occurs during PRV infection (revised Figure 1D and 6D) in the revised manuscript.

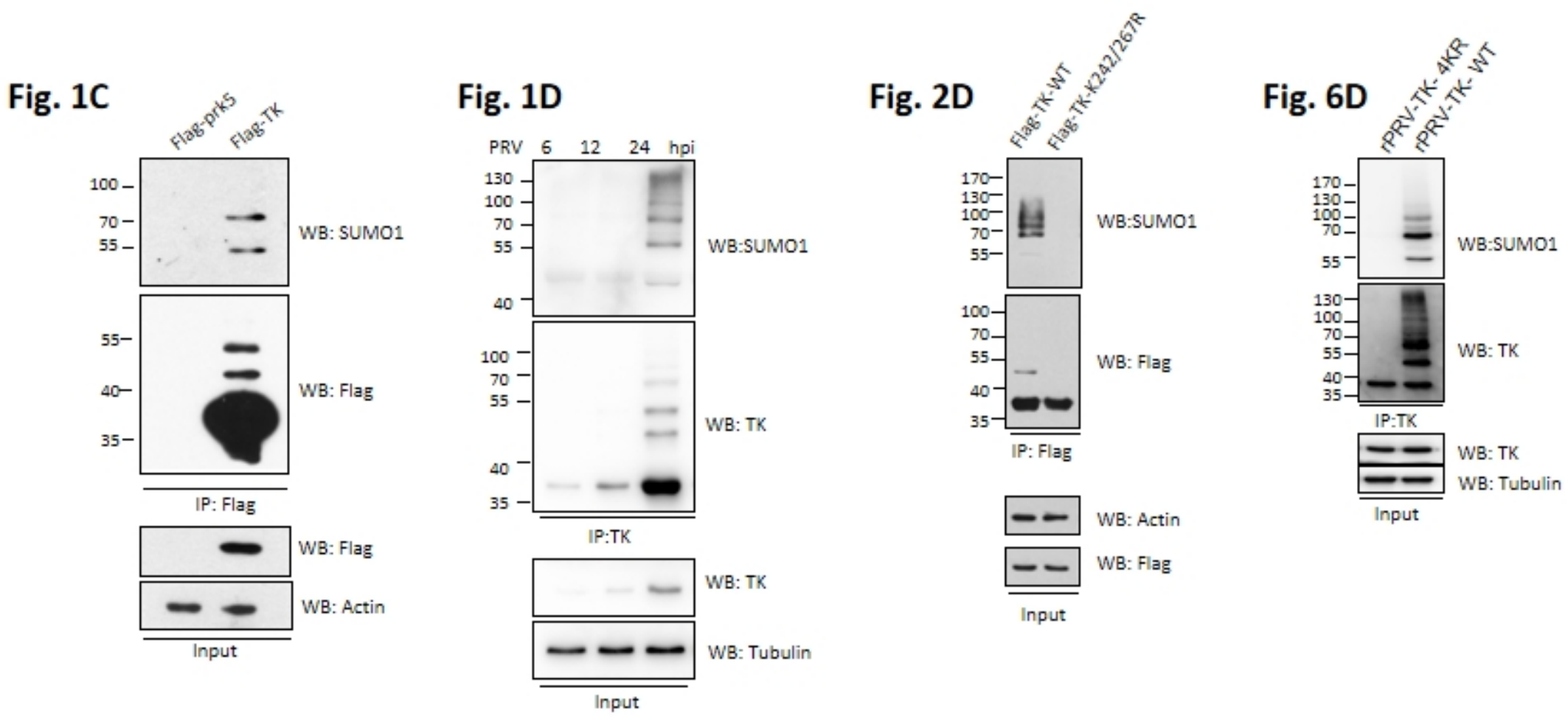

4. The conclusion that K242 and K267 are essential for SUMO-1 conjugation would be strengthened by constructing a recombinant PRV carrying the TK-K242/267R mutation and testing its SUMOylation in the context of infection. The authors should consider this or at least justify why it was not done.

We thank the reviewer for this insightful suggestion. We agree that generation of a recombinant PRV carrying the TK-K242/267R mutation would provide additional genetic evidence supporting the role of these residues in TK SUMOylation during authentic viral infection.

In this study, K242 and K267 were identified as the major SUMOylation sites of PRV TK based on systematic lysine mutagenesis and SUMOylation assays. Although structural prediction suggested that K16 and K56 might be less accessible or less favorable for SUMO conjugation, we could not completely exclude the possibility that these residues undergo weak or context-dependent SUMOylation, particularly during viral infection. Therefore, to ensure complete disruption of TK SUMOylation and avoid potential residual modification that could confound the interpretation of infection-based phenotypes, we generated the TK-4KR mutant virus containing mutations in all four identified candidate lysine residues.

We have clarified this rationale in the revised Discussion section (lines 442–452). We have also added additional data demonstrating that rPRV-TK-4KR completely abolished TK SUMOylation during authentic PRV infection (Fig. 6D), further supporting the use of the 4KR mutant for functional analyses.

5. Figure 3 (HSV-1 TK SUMOylation) is peripheral to the main story. The data could be moved to supplementary material or merged into Figure 2 as an extension; it does not warrant a separate main figure.

As suggested, we have moved the HSV-1-related experiments from the main figures to Supplementary data to better maintain the focus of the manuscript. In addition, we also performed a multiple sequence alignment of TK proteins from several representative alphaherpesviruses, including PRV, HSV-1, bovine herpesvirus 1 (BHV-1), equine herpesvirus 1 (EHV-1), and Marek's disease virus (MDV). These new data have been included as Supplementary Fig. S4 and are discussed in the revised Discussion (lines 453 - 468).

6. In the nuclear cytoplasmic fractionation experiment (Fig. 4B), the input panels lack nuclear marker controls. Please add these markers to validate fraction purity. Moreover, as in point #4, a recombinant PRV-TK-K242/267R would provide stronger evidence that SUMOylation drives nuclear localization during infection.

We thank the reviewer for pointing out this oversight. The Lamin B1 immunoblot was inadvertently omitted from the original figure during figure preparation. In the revised manuscript, we have restored the Lamin B1 panel in revised Fig. 4B as a nuclear marker, together with Tubulin as the cytoplasmic marker, to validate the quality of the fractionation.

Fig. 4B

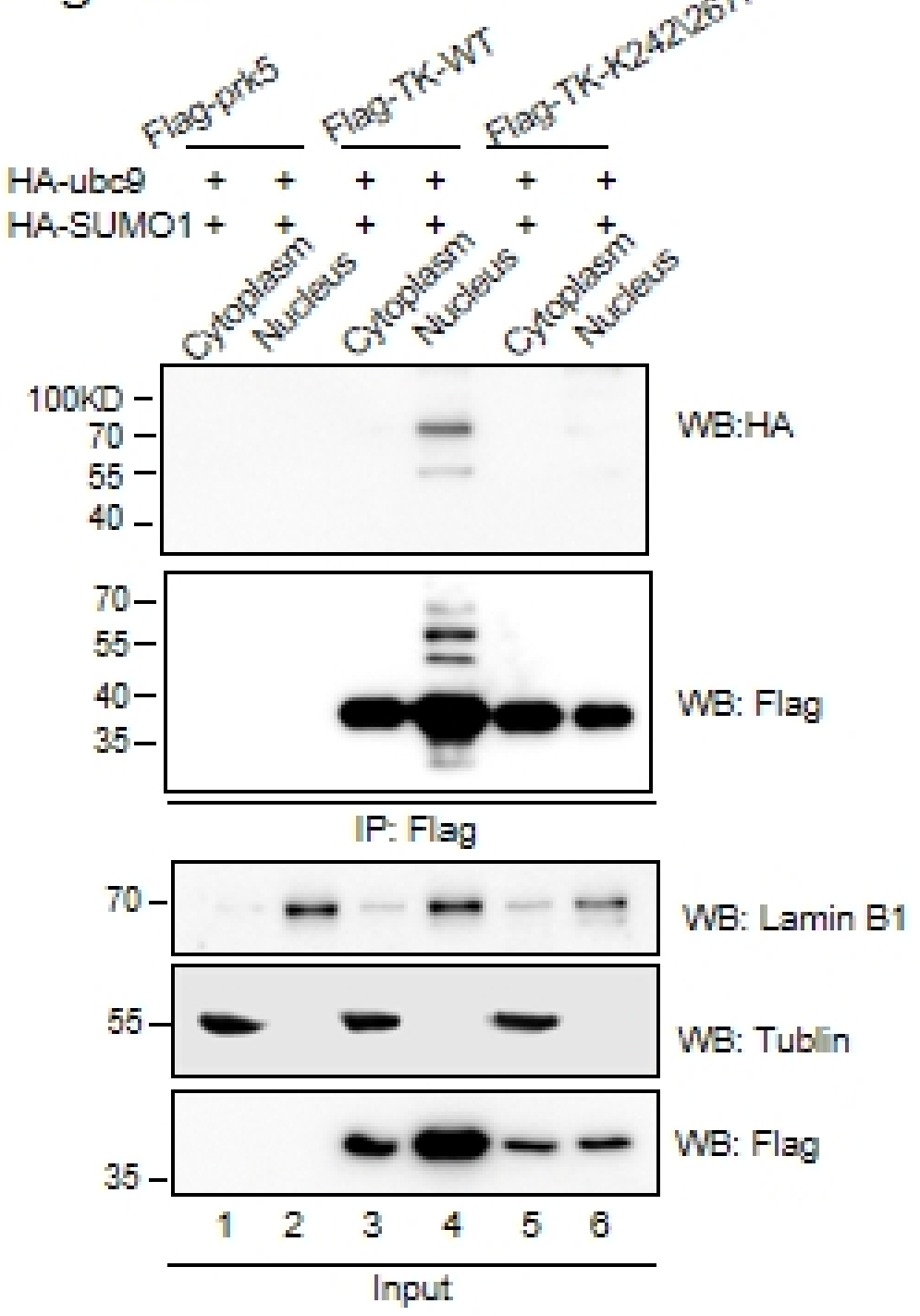

7. Figure 5A shows that TK-K242/267R appears to degrade faster than TK-WT upon CHX chase. Additionally, in Figure 5B input, Flag-TK-K242/267R and Flag-TK-4KR levels are visibly lower than Flag-TK-WT. These observations contradict the authors' statement that SUMOylation does not affect TK stability. Please provide an explanation – e.g., quantification with

We repeated these experiments several times and did not found the significant difference between TK-mutants and TK-WT. To address this concern, we performed quantitative analysis of TK bands from three independent experiments.

Revised Fig3A

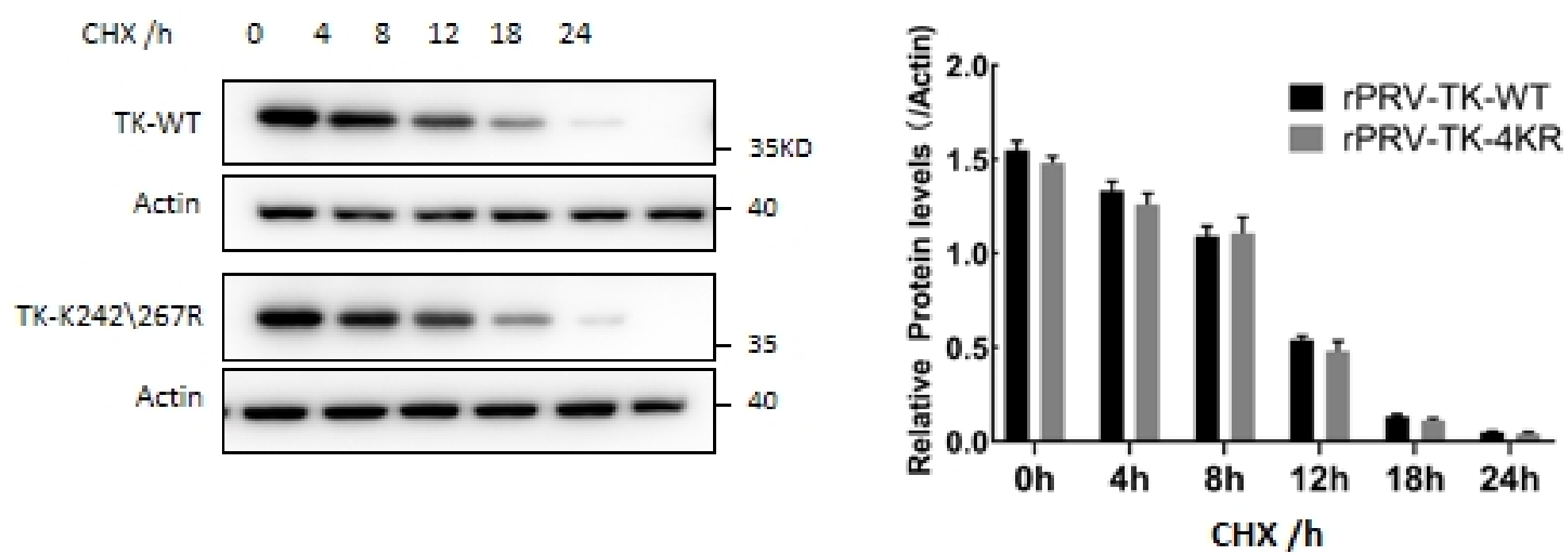

8. Figure 3B: The sequence alignment between PRV and HSV-1 TK is used to explain lower SUMOylation efficiency of HSV-1 TK. However, the two proteins share low overall similarity, and the lack of corresponding lysines is only one possible explanation. This

speculation would be more appropriate in the Discussion rather than presented as a definitive result.

We thank the reviewer for this valuable comment. We agree that differences in individual lysine residues represent only one possible factor affecting SUMOylation efficiency and that the contribution of other sequence or structural features cannot be excluded. We have therefore revised the presentation of this analysis and removed the HSV-1 TK SUMOylation comparison from the main figures, while moving these data to the Supplementary Materials (Figure S4). We have also modified the corresponding text in the Discussion (lines 453 – 468) to avoid presenting the relationship between lysine variation and SUMOylation efficiency as a definitive conclusion.

It is worth noting that PRV TK and HSV-1 TK are highly conserved homologous proteins within the alphaherpesvirus family, sharing substantial sequence similarity and conserved functional domains. Therefore, comparison of their SUMOylation properties may provide insights into the evolutionary conservation and divergence of TK regulation among alphaherpesviruses. To further investigate this possibility, we performed multiple sequence alignment of TK proteins from representative alphaherpesviruses, including PRV, HSV-1, bovine herpesvirus 1 (BHV-1), equine herpesvirus 1 (EHV-1), and Marek's disease virus (MDV). These data have been included as Supplementary Figure S4.

The alignment revealed that the identified SUMOylation-associated lysine residues show differential conservation among alphaherpesviruses. Specifically, K16 is completely conserved, whereas K242 is replaced by arginine only in HSV-1, and K267 is replaced by arginine only in BHV-1. In contrast, K56 shows lower conservation and is retained in only a subset of viruses, including PRV and EHV-1. Among the analyzed viruses, PRV and EHV-1 retain the highest conservation of the four lysine residues identified in this study.

These observations suggest that TK SUMOylation may represent a conserved regulatory mechanism among alphaherpesviruses, while virus-specific sequence divergence may influence SUMOylation efficiency. Although the reduced SUMOylation of HSV-1 TK observed in our supplementary experiments may be associated with the absence of certain conserved lysine residues (such as K56 and K242), we acknowledge that additional sequence or structural determinants may also contribute to this difference. We have revised the Discussion accordingly.

Revised Fig. S4

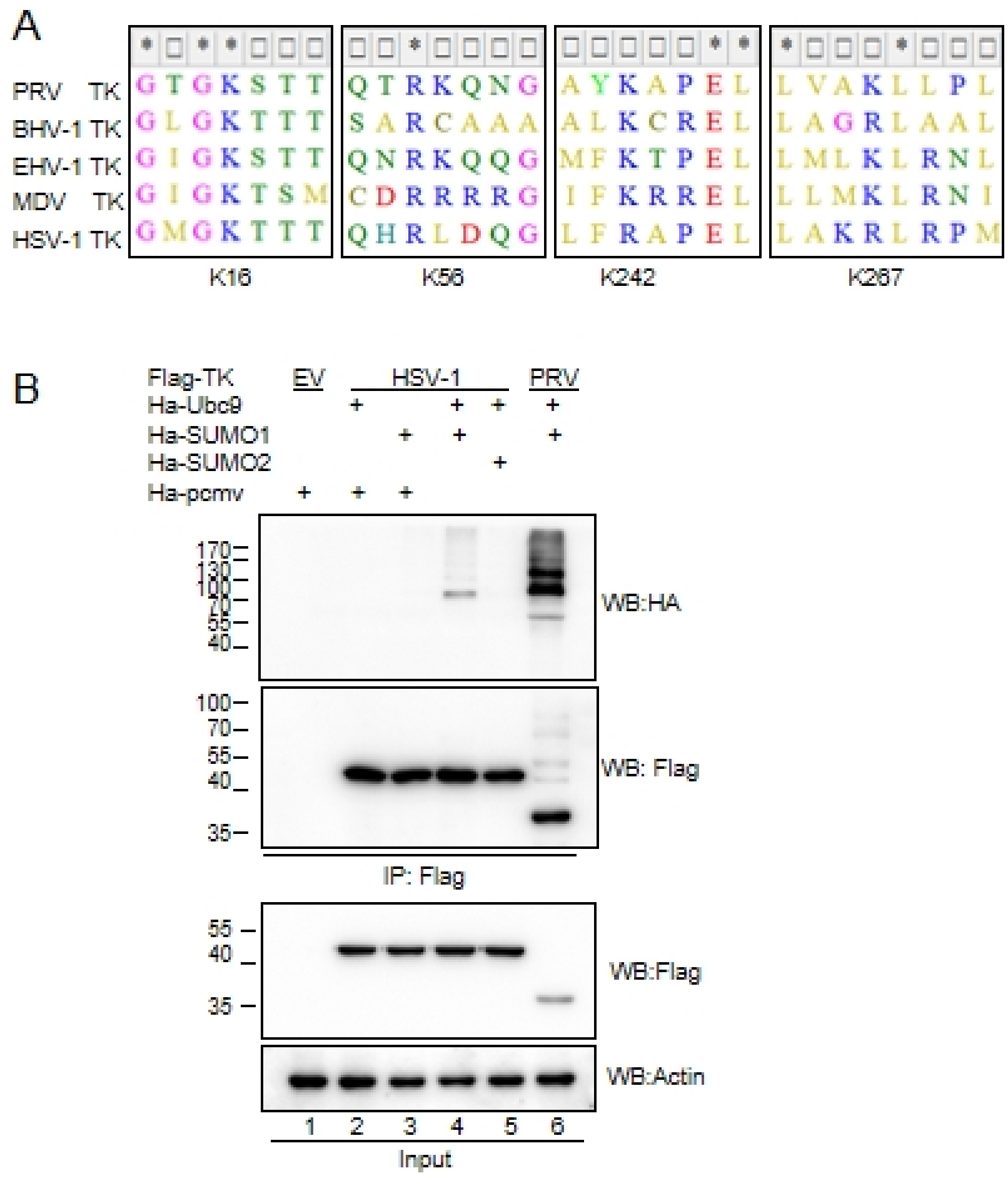

9. The manuscript would benefit from professional language editing to improve readability, correct minor grammatical errors, and remove over statements (e.g., “unprecedented”, “first report”).

The manuscript has been carefully revised throughout to improve clarity and readability. We have corrected grammatical and stylistic issues, refined sentence structure, and moderated statements that could be interpreted as overstated.

Reviewer #3:

This manuscript, “SUMOylation of Pseudorabies Virus Thymidine Kinase Controls Its Nuclear Localization and Promotes Neuronal Replication and Reactivation,” by Chen et al, investigates the post-translational regulation of PRV thymidine kinase (TK) by SUMOylation and proposes that SUMO modification controls TK nuclear localization, neuronal replication, reactivation from latency, and viral pathogenicity. The study addresses an interesting and important question, particularly given the known importance of TK in alphaherpesvirus neurotropism and latency/reactivation biology. The authors present biochemical, cell biological, recombinant virus, neuronal replication, and in vivo infection data suggesting that TK is SUMO-modified and that mutation of SUMOylation

sites attenuates neuronal infection and virulence. While the manuscript contains several interesting observations, the mechanistic conclusions are currently overstated relative to the evidence presented. The central claim, that direct SUMOylation of TK at K242 and K267 regulates nuclear localization and controls neuronal replication/reactivation, is not conclusively demonstrated. The manuscript lacks critical mechanistic experiments demonstrating endogenous TK SUMOylation during infection, preservation of TK enzymatic activity in the 4KR mutant, and definitive evidence linking the neuronal and in vivo phenotypes specifically to SUMOylation rather than broader functional disruption of TK.

Major Issues: Key Experiments Required for Acceptance:

1. The SUMOylation assays rely on native immunoprecipitation conditions using overexpressed HA-SUMO and Ubc9. Under these conditions, the HA-reactive high-molecular-weight band could be from SUMOylated interacting proteins.

We thank the reviewer for this insightful comment. We agree that under native immunoprecipitation conditions, SUMO-associated high-molecular-weight bands could theoretically arise either from direct SUMO conjugation of TK or from SUMOylated interacting proteins associated with TK-containing complexes.

In our original study, native immunoprecipitation was used as an initial approach to examine TK SUMOylation. Nevertheless, several observations already supported the conclusion that TK itself is SUMO modified. First, mutation of the predicted SUMO acceptor lysine residues, particularly K242 and K267, markedly abolished the SUMO-associated high-molecular-weight signals (Fig. 2). Such site-specific loss would be unlikely if the detected signals originated primarily from SUMOylated interacting proteins. Second, the reduction in SUMOylation was consistently accompanied by impaired nuclear localization (Fig. 4) as well as defects in neuronal replication and reactivation (Fig. 6 and 7), supporting the functional relevance of TK SUMOylation.

To directly address the reviewer's concern, we have now performed additional endogenous SUMOylation assays under denaturing SUMO immunoprecipitation (denaturing SUMO-IP) conditions and included these data in the revised manuscript (Figs. 1D, 2D, and 6D). Under the denaturing SUMO-IP conditions, endogenous SUMO-modified TK remained readily detectable in both ectopic expression and viral infection settings, providing evidence that TK is directly conjugated by endogenous SUMO rather than indirectly associated with SUMOylated interacting proteins.

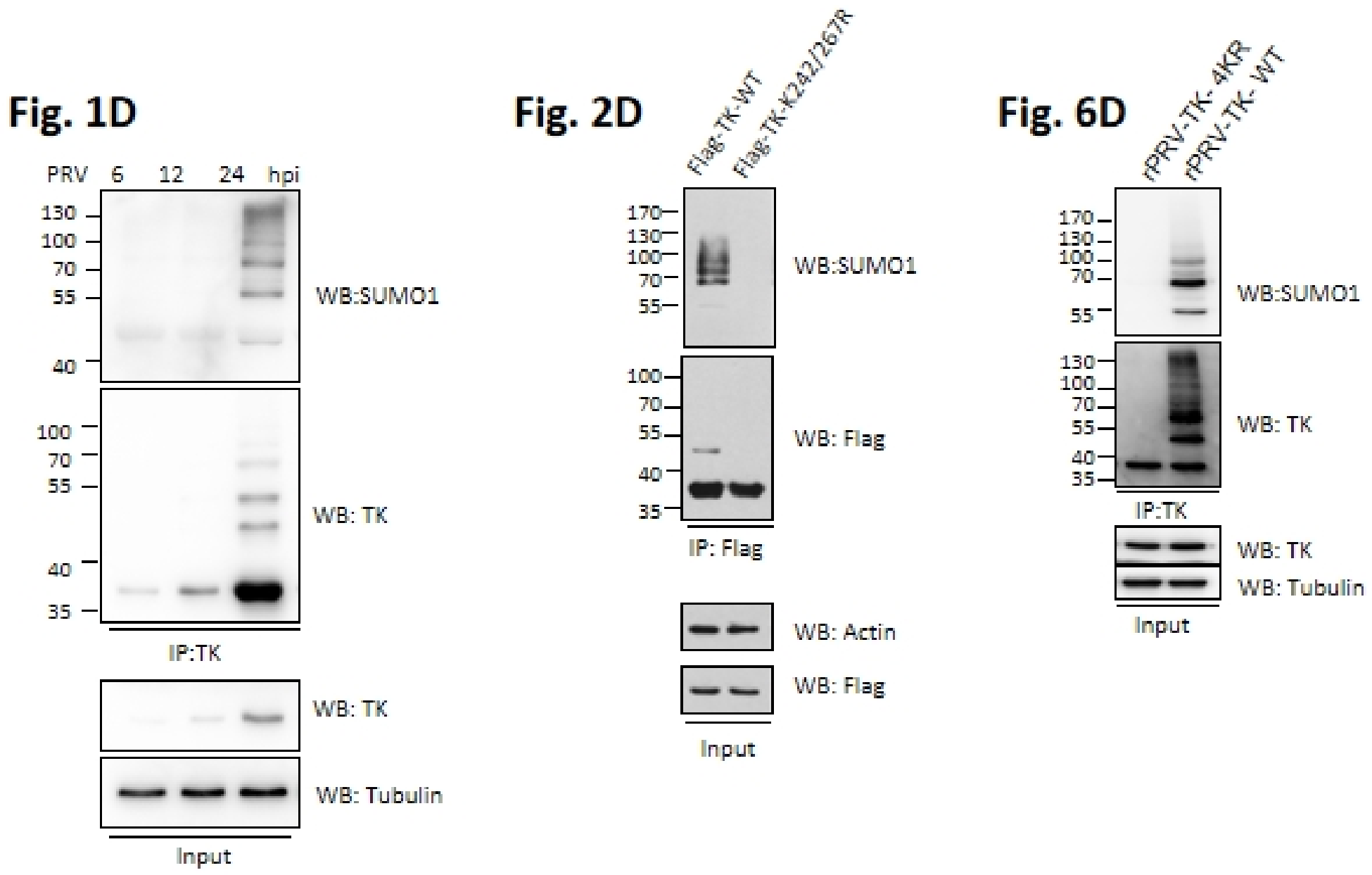

2. The mutagenesis data suggest that K242 and K267 contribute to SUMO-associated high-molecular-weight species, but the data do not conclusively establish these residues as direct SUMO acceptor sites, as neither single nor combined mutations completely abolish HA-reactive bands, and the 4KR mutant still retains residual SUMO-associated signals.

In our original Figures 2C, the K242/267R double mutant and the 4KR mutant reduced the SUMO-associated high-molecular-weight signals to nearly background levels, supporting the conclusion that K242 and K267 are the major residues contributing to TK SUMOylation.

To further strengthen this conclusion, we have now additionally performed endogenous SUMOylation assays in both overexpression (Figure 2D) and infection conditions(Figure 6D), and the results show that the K242/267R double mutant and the 4KR mutant reduced the endogenous SUMO-associated high-molecular-weight signals to nearly background levels.

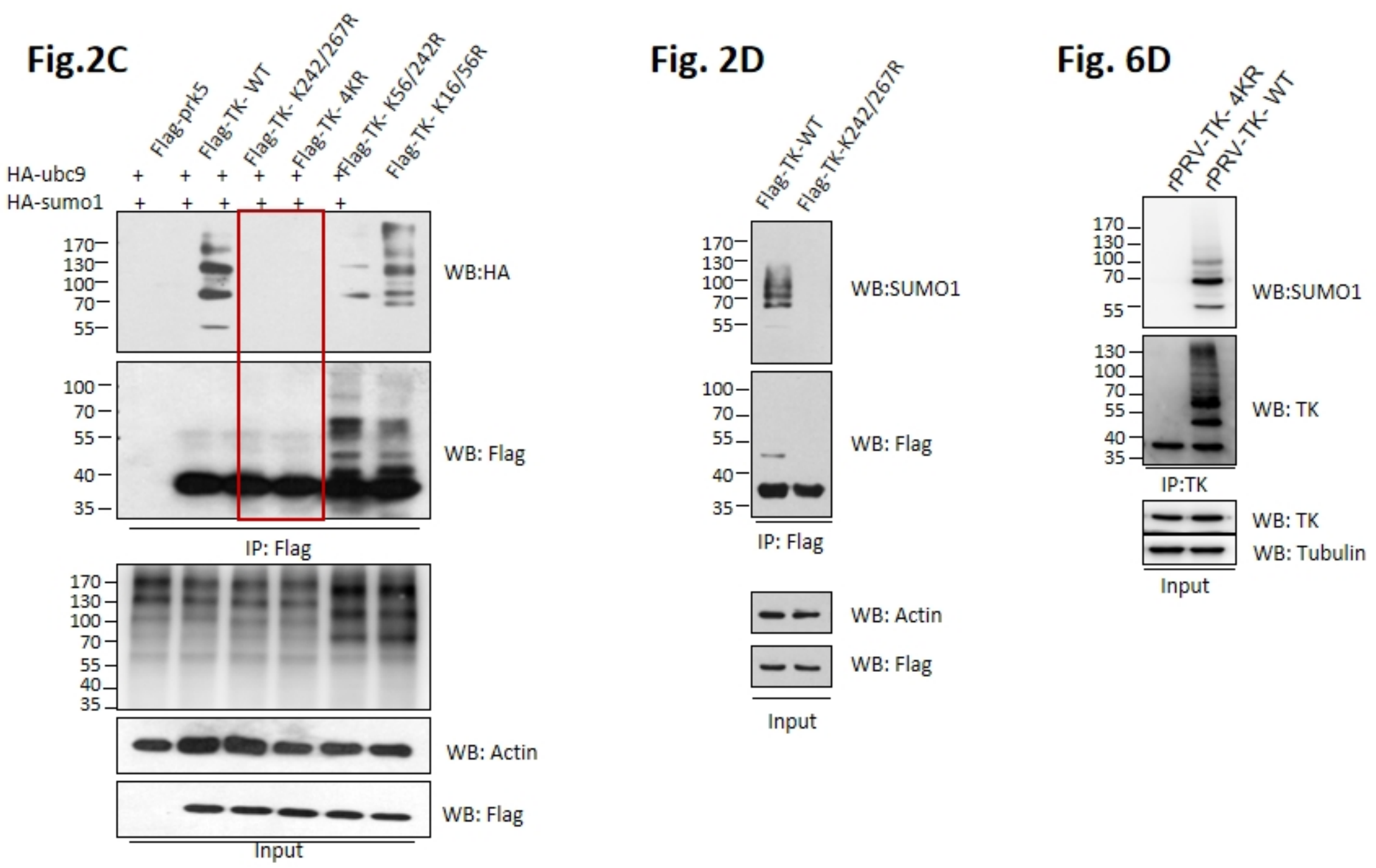

3. TK is a critical viral kinase required for nucleotide metabolism and efficient replication in neurons and non-dividing cells. The observed defects in neuronal replication, reactivation, and virulence could therefore result from impaired TK catalytic function rather than altered SUMOylation. Without demonstrating that the mutant retains normal TK function aside from SUMOylation deficiency, the mechanistic interpretation remains unclear.

We thank the reviewer for this important comment and agree that it is essential to distinguish the effects of impaired TK catalytic activity from those resulting from loss of SUMOylation.

To directly address this concern, we performed an ADP-Glo™ kinase assay using purified Flag-TK-WT, Flag-TK-K242/267R, and Flag-TK-4KR proteins, and have included these data in the revised Fig. 4C. The ADP-Glo™ assay is a luminescence-based kinase activity assay that quantitatively measures ADP generated during ATP-dependent phosphorylation reactions, thereby providing a direct assessment of kinase catalytic activity.

As shown in the revised Fig. 4C, no significant differences in kinase activity were detected among Flag-TK-WT, Flag-TK-K242/267R, and Flag-TK-4KR. These results indicate that mutation of the SUMOylation sites does not impair the intrinsic catalytic activity of TK.

Fig. 4C

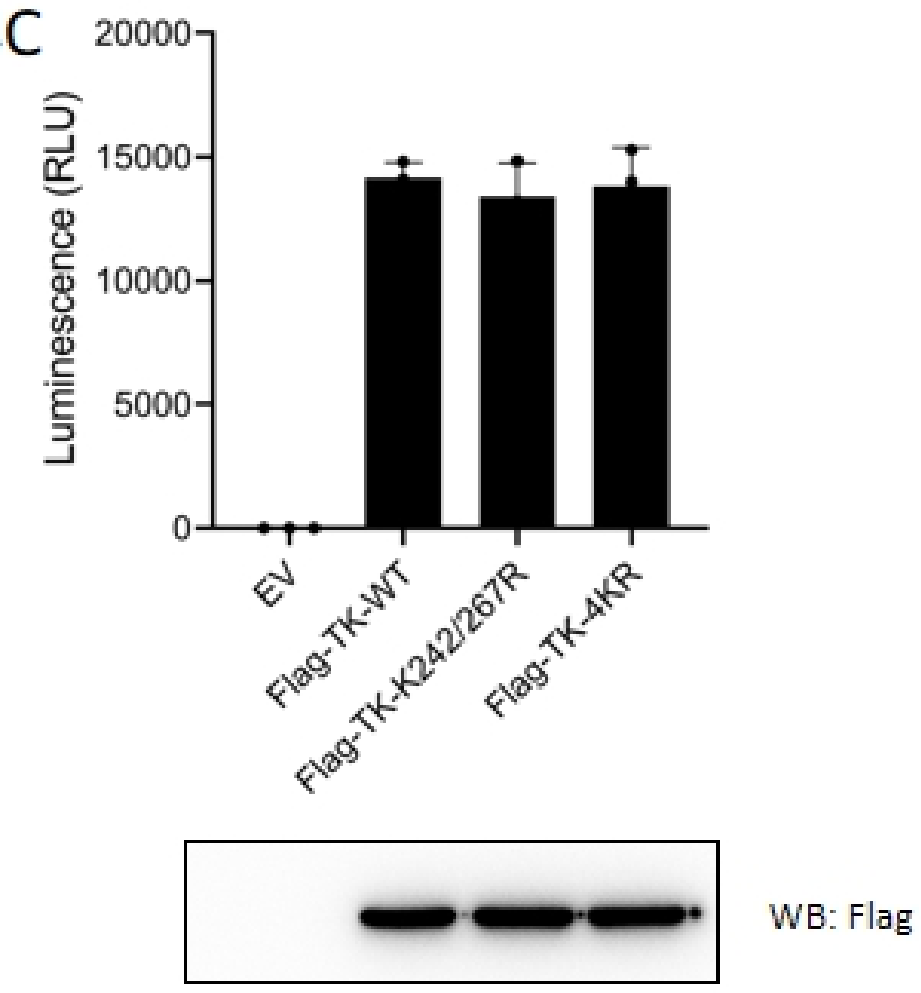

4. The SUMO-deficient mutants exhibit puncta-like localization patterns, which could represent aggregation or the formation of inclusion bodies, making TK sequestered away from the replication foci, thus leading to the phenotype.

We agree that punctate localization patterns can sometimes reflect protein aggregation or inclusion body formation, which could potentially contribute to altered intracellular distribution and functional defects. However, several observations from our study suggest that the observed phenotype is unlikely to result primarily from severe protein misfolding or aggregation.

First, the SUMOylation-deficient mutants retained kinase activity comparable to WT TK (revised Fig. 4C), indicating that the overall structural and catalytic integrity of the protein was largely preserved. Second, the mutants did not exhibit dramatic degradation or loss of expression (revised Fig. 3A) that would typically accompany severe aggregation-prone misfolding. Third, the altered localization pattern consistently correlated with reduced nuclear accumulation and impaired neuronal replication/reactivation phenotypes (revised Fig. 4-8).

Nevertheless, we agree that the current data do not fully exclude this possibility. Therefore, we have moderated the corresponding statements throughout the manuscript and revised the text to indicate that SUMOylation contributes to efficient nuclear localization of TK, rather than definitively controlling this process.

5. All SUMOylation experiments are performed using transient overexpression systems and does not demonstrate that endogenous TK becomes SUMOylated during authentic PRV infection, which is a major concern.

We agree that demonstrating endogenous SUMOylation of TK during authentic viral infection is essential for establishing the physiological relevance of our findings. To address the reviewer's concern, we have now included additional experiments in the TK and

mutations SUMOylation by endogenous SUMO1 (revised Figure 2D) as well as the endogenous SUMOylation of TK occurs during PRV infection (revised Figure 1D and 6D) in the revised manuscript.

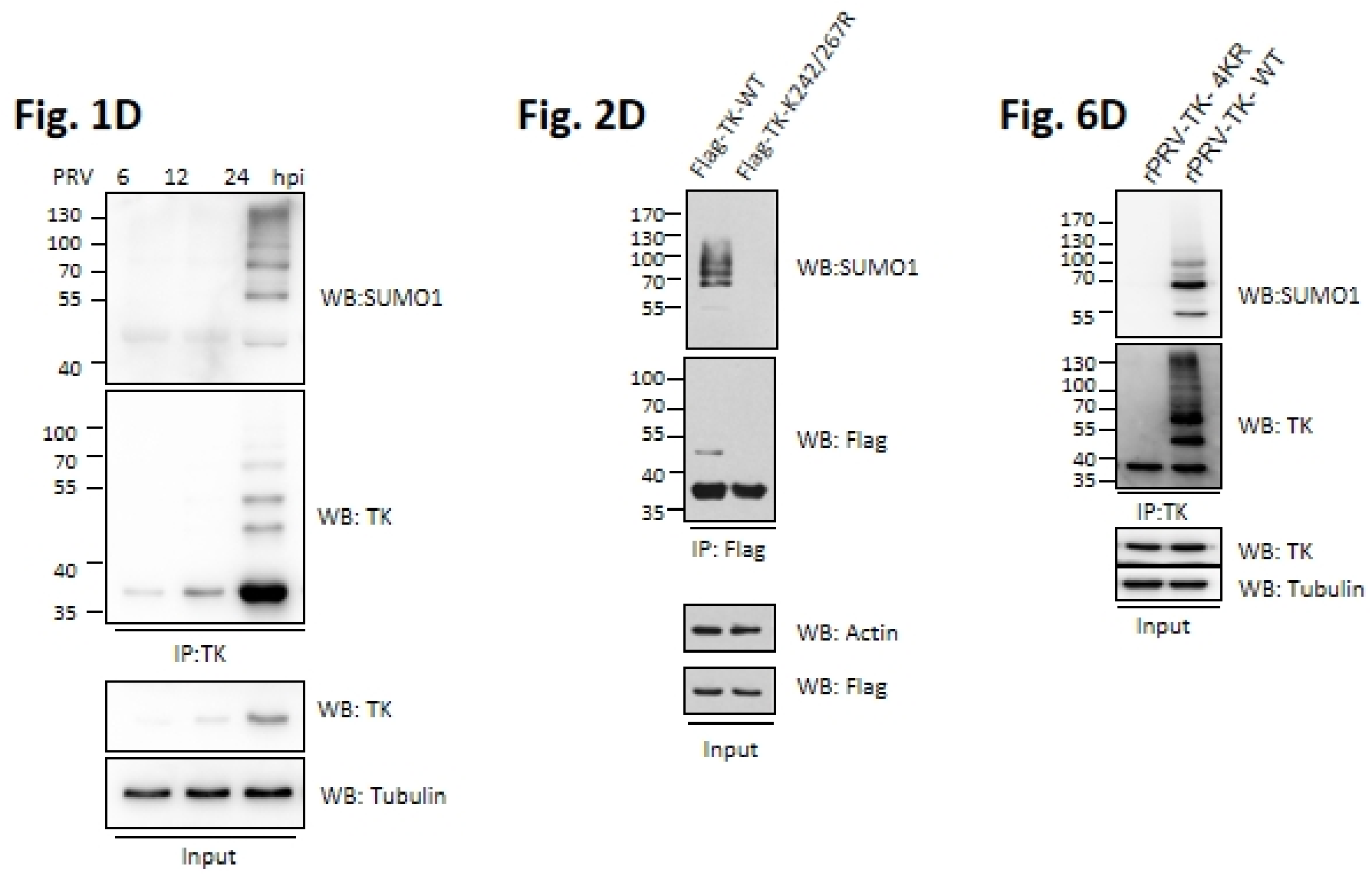

6. The 42°C "latency/reactivation" model in N2a cells is not rigorously established as a bona fide latency system. Elevated temperature may induce restricted replication. The manuscript does not show latency via episomal persistence or chromatinized viral genomes, which could impact "reactivation defects" observed in recombinant virus.

We thank the reviewer for this thoughtful comment. We agree that our temperature-shift model does not fully recapitulate all molecular features of authentic alphaherpesvirus latency *in vivo*, such as episomal persistence or chromatinization of viral genomes. We have therefore revised the manuscript to clarify that this system represents an *in vitro* latency-like/reactivation model rather than a bona fide latency model.

Importantly, in the present study, all viruses—including PRV-WT, PRV-TK-KO, PRV-US3-KO, PRV-TK/US3-KO, rPRV-TK-WT, and rPRV-TK-4KR—displayed indistinguishable viral genome levels, IE180/LAT transcription, viral protein expression, and infectious virus production after 3 days at 42 °C (revised Fig. 7A–E, left or upper panels), indicating that they entered comparable latency-like states before reactivation. Therefore, although this model does not fully reproduce every molecular hallmark of authentic latency, it provides a well-controlled experimental platform for comparing the relative reactivation capacities of different recombinant viruses under identical conditions.

We have revised the Results (lines 310-362) and Discussion (lines 521-535, 561-567) accordingly to avoid overstating the nature of this model and now consistently refer to it as an *in vitro* latency-like/reactivation model.

7. The recombinant viruses are insufficiently characterized, such as full-genome sequencing, which is particularly important because the recombinant WT virus itself appears partially attenuated relative to parental PRV-WT.

We thank the reviewer for this valuable suggestion. We agree that comprehensive genetic characterization of the recombinant viruses is important for excluding unintended genetic alterations.

To verify the integrity of the engineered viruses, we sequenced the entire modified genomic region, including the TK expression cassette and both insertion junctions, in rPRV-TK-WT and rPRV-TK-4KR. The sequencing results confirmed correct insertion into the intended Us3 locus and verified the presence of the designed 4KR mutations, with no unintended sequence changes detected within the engineered region. These data have now been included as Supplementary Fig. S1. Although whole-genome sequencing would provide an even more comprehensive assessment, the sequencing of all genetically modified regions confirms the integrity of the recombinant viruses at the sites directly relevant to this study.

Fig. S1

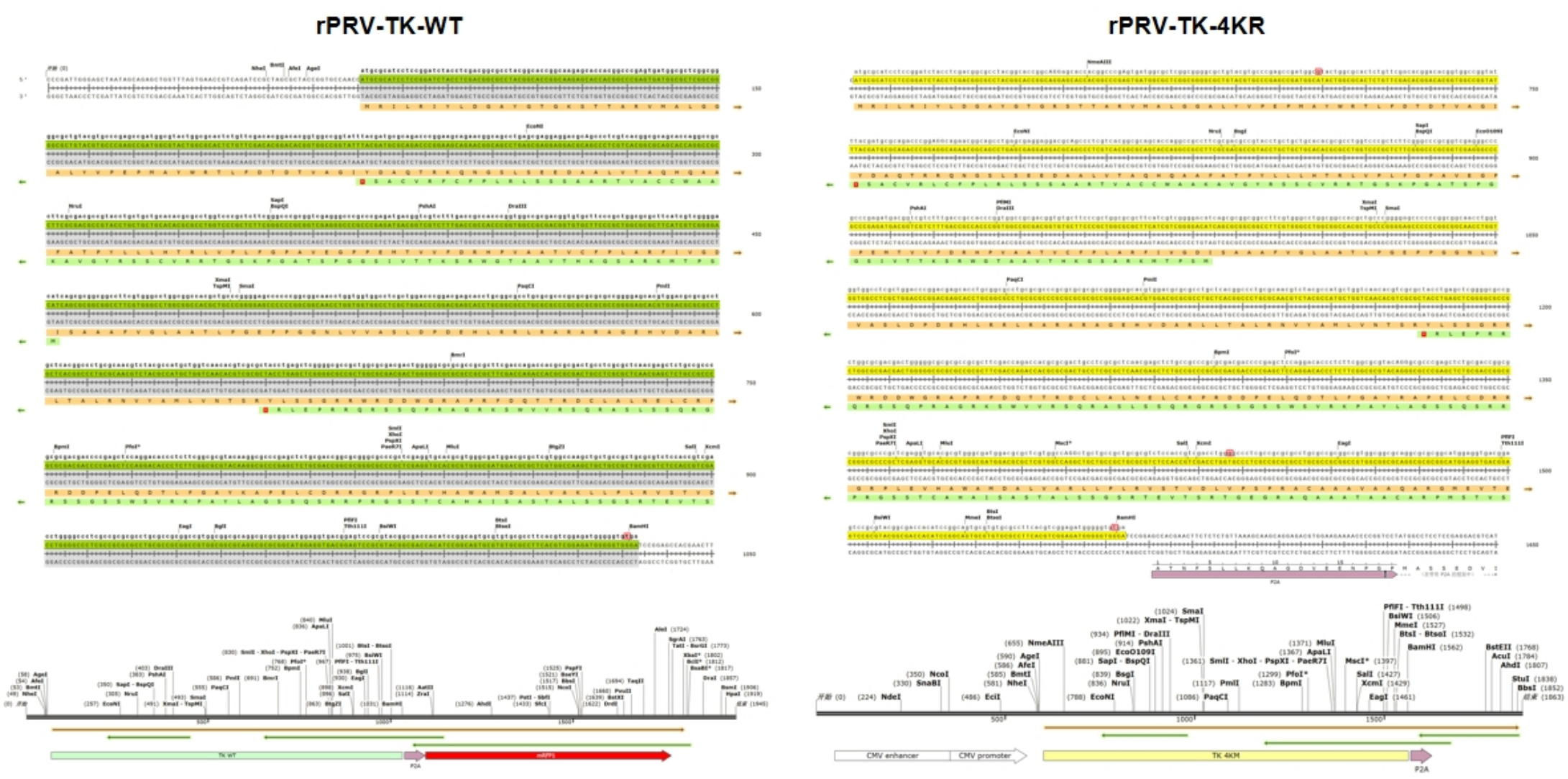

To further address the reviewer's concern regarding the attenuated phenotype of rPRV-TK-WT, we repeated the mouse infection experiments using an increased sample size (n = 6 mice per group) and included an additional PRV-US3-KO control virus. The repeated experiments yielded results consistent with those in the original manuscript. As expected, PRV-US3-KO exhibited delayed mortality compared with PRV-WT, consistent with the established role of Us3 in neurovirulence. Importantly, despite this attenuation, rPRV-TK-WT remained significantly more virulent than rPRV-TK-4KR, exhibiting an 80% mortality rate, greater body-weight loss, higher viral genome loads in tissues, and more severe histopathological lesions. These findings demonstrate that the phenotype associated with loss of TK SUMOylation cannot be explained solely by disruption of the Us3 locus.

We further speculate that the additional attenuation observed in rPRV-TK-WT relative to PRV-US3-KO is unlikely to result from unintended mutations, but more likely reflects altered TK expression kinetics and/or expression levels caused by ectopic insertion of the TK expression cassette into the Us3 locus, which differs from the endogenous regulation of the native TK gene during infection. We have added this interpretation to the Results

(lines 378-382) and Discussion (lines 536-551) and acknowledge it as a potential limitation of the current recombinant virus strategy.

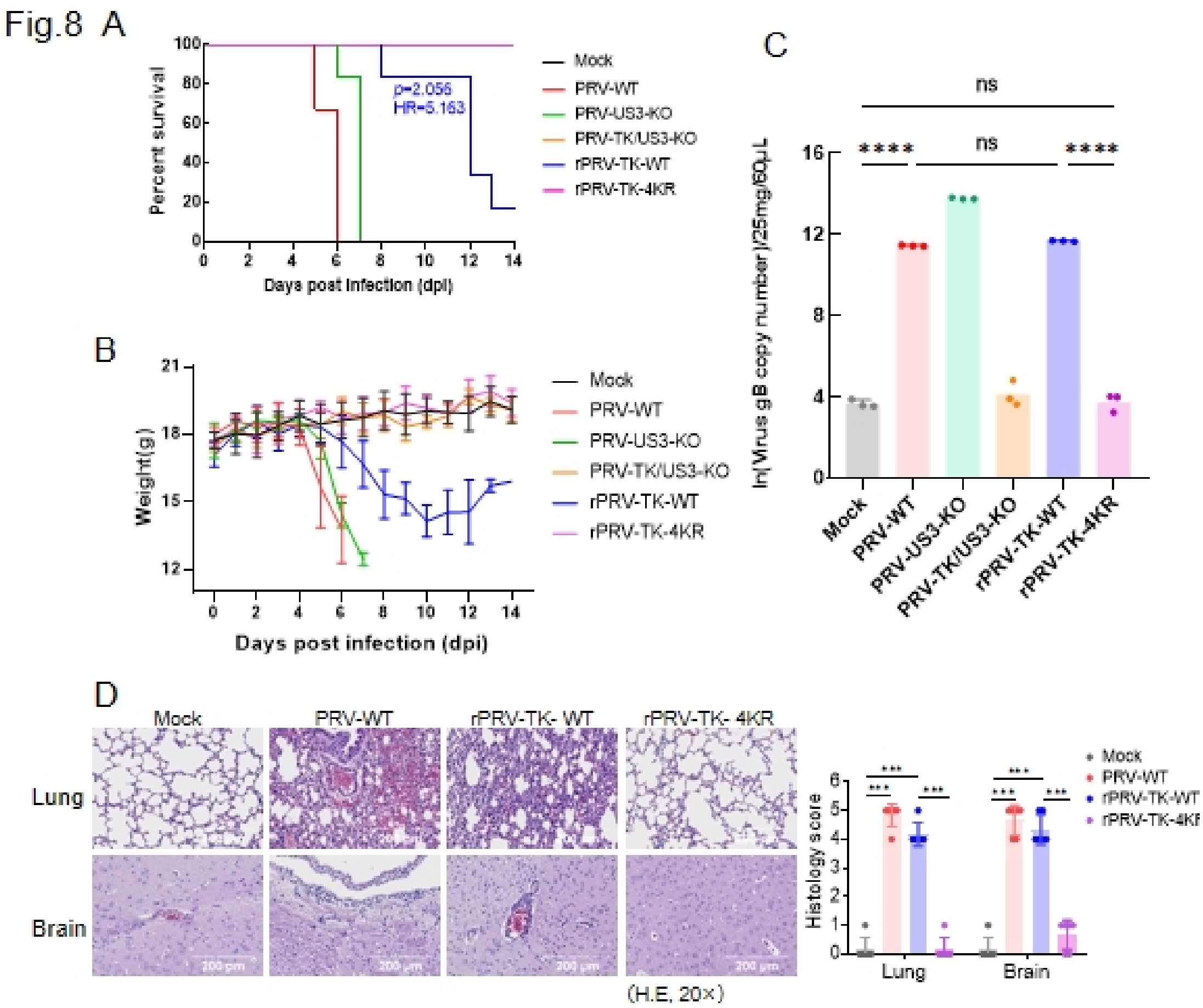

8. The mouse infection experiments use only n = 3 animals per group, which is insufficient for robust conclusions regarding virulence and pathogenesis. Additionally, the histopathology is descriptive, and the survival statistics are absent.

In the revised manuscript, we repeated the animal infection experiments and increased the number of mice to six animals per group (n = 6). The repeated experiments yielded results consistent with those reported originally, further confirming the reproducibility of our findings.

We have also included Kaplan–Meier survival curves together with statistical analyses in the revised Fig. 8A. In addition to the log-rank (Mantel–Cox) test, we further analyzed the median survival time and hazard ratio (HR). The survival trend, together with the hazard ratio, consistently supports the conclusion that loss of TK SUMOylation markedly attenuates PRV virulence.

In addition, we expanded the pathological evaluation by combining histopathological examination scoring (revised Fig.8D) with quantitative viral genome analysis (revised Fig.8C) in infected tissues. These data showed that rPRV-TK-4KR exhibited markedly reduced viral loads and milder pathological lesions than rPRV-TK-WT.

9. Throughout the manuscript, the language often implies definitive mechanistic causality that does not seem to be fully supported. For example, SUMOylation controls TK nuclear localization, and SUMOylation promotes reactivation. SUMOylation regulates virulence" should be moderated unless stronger mechanistic evidence is provided.

As suggested, the manuscript has been carefully revised throughout to moderate statements that could be interpreted as overstated.

Minor Issues: Editorial and Data Presentation Modifications

1. Many immunoblots are overexposed and contain diffuse smears that are difficult to interpret quantitatively.

We agree that several immunoblots in the original manuscript were not presented optimally. Some of the western blot images were acquired using X-ray film, which in certain cases resulted in overexposure and diffuse high-molecular-weight signals, particularly in the SUMOylation assays.

In the revised manuscript, we have carefully re-examined the original western blot data and, wherever possible, replaced the images with scans acquired using a digital imaging system or with shorter exposure times to ensure that the signals fall within the linear detection range and are more clearly presented.

2. Figures lack densitometric quantification and statistical analysis.

In the revised manuscript, we have added densitometric quantification and statistical analyses for the key immunoblot experiments in which quantitative comparison was essential to support our conclusions, such as the revised Fig. 3A. In addition, the immunofluorescence experiments have now been quantitatively analyzed by scoring the percentages of cells exhibiting nuclear, cytoplasmic, or mixed localization patterns, included in revised Figs. 4A and 6C.

For western blot experiments showing clear qualitative differences (e.g., presence or absence of protein expression or viral proteins), we present representative blots from multiple independent experiments. The corresponding conclusions are supported by repeated biological replicates and, where appropriate, complementary assays (e.g., qPCR, $TCID_{50}$ assays, or immunofluorescence).

3. NEM or SUMO protease inhibitors during lysis are not described and should be clarified.

We have now clarified the use of SUMOylation-preserving conditions during sample preparation in the revised Materials and Methods section (lines 646–672).Specifically, N-ethylmaleimide (NEM) was added to the lysis buffer to inhibit SUMO-specific protease activity and prevent deSUMOylation during protein extraction.

4. Scale bars are missing from several microscopy panels.

Sorry for the mistake and we have add the scale bars in the revised microscopy panels.

5. Confocal imaging would improve the interpretation of localization phenotypes.

In the revised manuscript, all immunofluorescence images have been acquired using a Leica confocal laser scanning microscope, which provide improved resolution for evaluating the subcellular localization of TK.

6. The manuscript should include quantitative nuclear/cytoplasmic localization analysis with replicate statistics.

In the revised manuscript, we performed quantitative analysis of TK subcellular localization by scoring the percentages of cells exhibiting nuclear, cytoplasmic, or both nuclear and cytoplasmic localization. At least 50 cells from three independent experiments were analyzed. The percentage of cells in each localization category is now presented in the revised Fig. 4A and Fig. 6C.

7. Fractionation experiments require additional nuclear and cytoplasmic purity markers.

We thank the reviewer for pointing out this oversight. The Lamin B1 immunoblot was inadvertently omitted from the original figure during figure preparation. In the revised manuscript, we have restored the Lamin B1 panel in Fig. 4B as a nuclear marker, together with Tubulin as the cytoplasmic marker, to validate the quality of the fractionation.

Fig. 4B

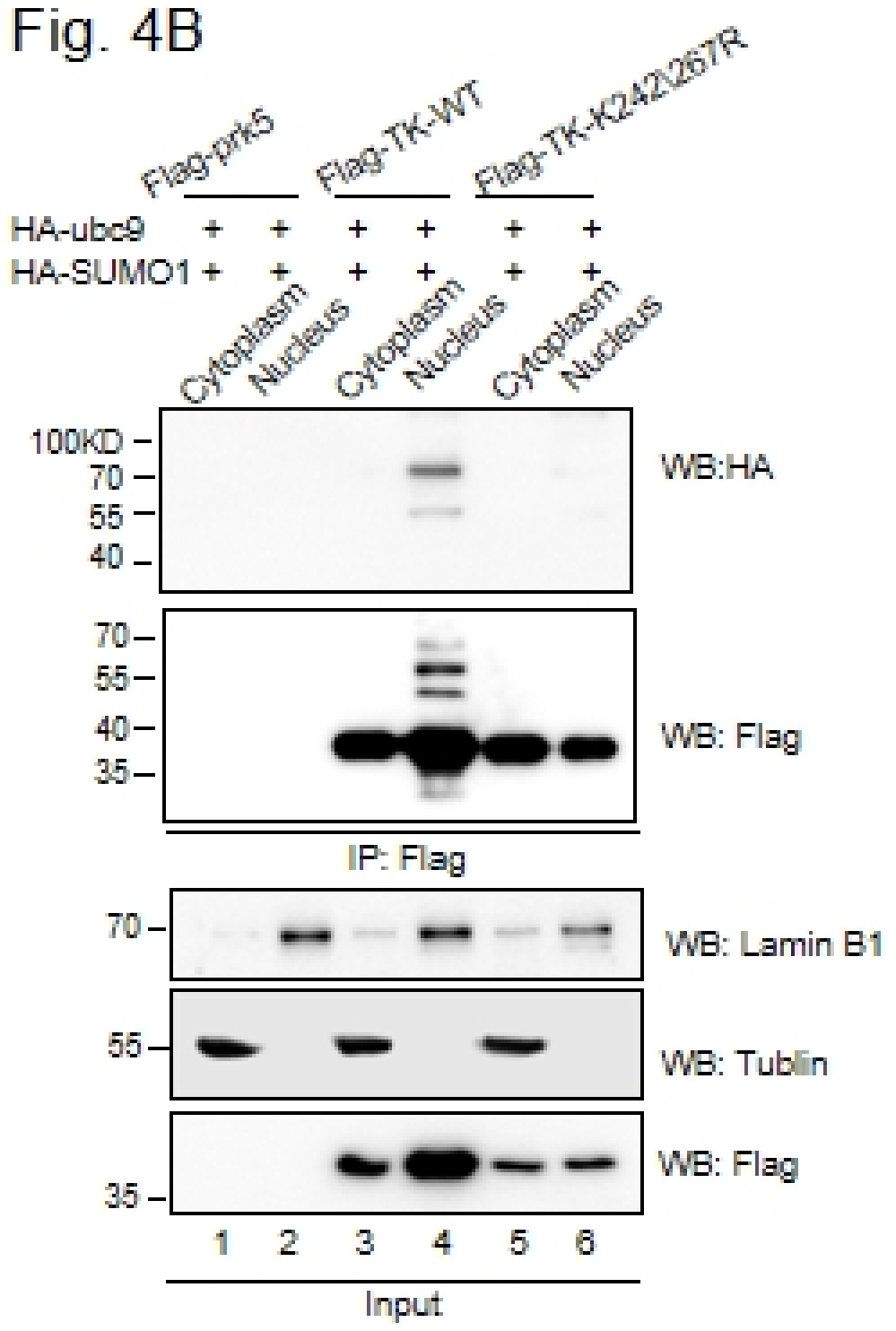

8. Figure legends contain occasional formatting inconsistencies and duplicated text.

We have carefully reviewed all figure legends throughout the revised manuscript and corrected formatting inconsistencies, removed duplicated text, and standardized the presentation to improve clarity and consistency.

9. Statistical methods require a more detailed description, including biological vs technical replicates and n values.

We have expanded the Statistical Analysis section to provide a more detailed description of the statistical methods used in this study (lines 800-812). Specifically, we have clarified the definitions of biological and technical replicates, indicated that all *in vitro* experiments were performed with three independent biological replicates unless otherwise stated, and specified that each qPCR measurement was conducted in technical triplicate. In addition, the number of animals used in each *in vivo* experiment has been provided in both the Materials and Methods section and the corresponding figure legends. The statistical tests used for each analysis have also been clarified in the revised manuscript.

10. The nomenclature of mutants and recombinant viruses should be standardized throughout the manuscript.

We have carefully reviewed the entire manuscript and standardized the nomenclature of all TK mutants and recombinant viruses.

11. Additional neuronal models or primary neurons would substantially strengthen the biological relevance.

We agree that validation in additional neuronal models, particularly primary neurons, would further strengthen the physiological relevance of our findings. In the present study, we selected N2a cells because they are a well-established neuronal model that has been widely used to investigate PRV replication, latency, and reactivation. Using this model, we consistently observed that disruption of TK SUMOylation impaired TK nuclear localization, viral replication, and reactivation.

Although validation in primary neurons or additional neuronal cell types is beyond the scope of the current study, we have acknowledged this limitation and highlighted it as an important direction for future investigation in the revised Discussion (lines 561–567).

12. The manuscript would benefit from a discussion of whether SUMOylation affects TK interaction with known viral or cellular partners.

We agree that, in addition to regulating subcellular localization, SUMOylation may also influence the interaction of TK with viral and/or host proteins, thereby contributing to its biological functions.

In the revised Discussion, we have expanded this section to address this possibility. Specifically, we discuss that SUMO modification frequently regulates protein–protein interactions by creating binding surfaces for proteins containing SUMO-interacting motifs (SIMs) or by inducing conformational changes that alter binding affinity. We therefore propose that SUMOylation of PRV TK may not only facilitate its nuclear accumulation but also modulate its interaction with host nuclear transport factors and/or viral replication proteins, thereby promoting efficient viral replication and reactivation. At present, the interacting partners of PRV TK that are regulated by SUMOylation remain unknown and will be investigated in future studies. This discussion has been incorporated into the revised manuscript (lines 473–502).

Reviewer #4:

This study identifies Pseudorabies virus (PRV) Thymidine Kinase (TK) as a novel substrate for SUMOylation at residues K242 and K267. The authors demonstrate that this post-translational modification is essential for the nuclear localization of TK. While dispensable in epithelial cells (PK15), TK SUMOylation is shown to be critical for viral replication in neuronal cells (N2a), reactivation from latency, and overall neurovirulence in a mouse model. The discovery of a host-mediated PTM regulating a key viral metabolic enzyme's trafficking provides significant insights into alphaherpesvirus neurotropism. However, several mechanistic details and experimental controls require strengthening to meet the high standards of PLOS Pathogens.

Major Issues: Key Experiments Required for Acceptance:

1. The Figure 4A shows only a single representative cell per condition. This is insufficient to demonstrate the consistency of the phenotype across the cell population; the authors should provide images with a larger field of view (FOV) showing multiple cells. A formal statistical analysis is required to quantify the percentage of cells exhibiting "Nuclear," "Cytoplasmic," or "Both" localization patterns.

We thank the reviewer for this valuable suggestion. In the revised manuscript, we have replaced the images in Fig. 4A with larger fields of view, with each image displaying 2–4 representative cells for each experimental condition, thereby providing a more comprehensive view of the localization pattern.

In addition, we performed quantitative analysis of TK subcellular localization by scoring the percentages of cells exhibiting nuclear, cytoplasmic, or both nuclear and cytoplasmic localization. At least 50 cells from three independent experiments were analyzed. The percentage of cells in each localization category is now presented in the revised Fig. 4A.

Fig.4 A

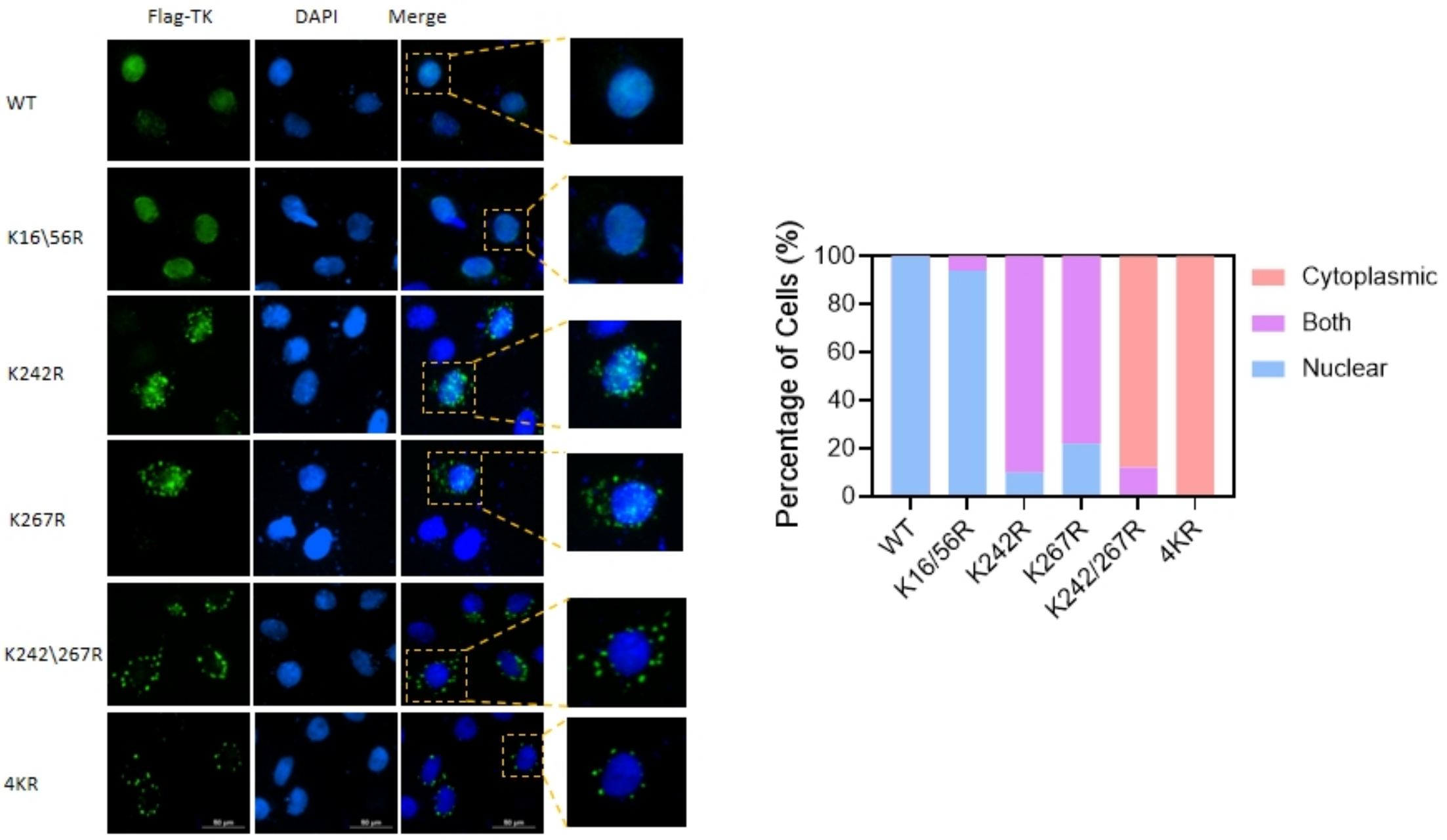

2. In figure 4B, the authors use Tubulin as a cytoplasmic marker to verify fractionation, however, a specific nuclear marker (e.g., Lamin B1 or Histone H3) is mandatory to demonstrate the purity of the nuclear fraction and the absence of cross-contamination . Furthermore, to objectively confirm that SUMOylation deficiency increases cytoplasmic distribution.

We thank the reviewer for pointing out this oversight. The Lamin B1 immunoblot was inadvertently omitted from the original figure during figure preparation. In the revised manuscript, we have restored the Lamin B1 panel in Fig. 4B as a nuclear marker, together with Tubulin as the cytoplasmic marker, to validate the quality of the fractionation.

Fig. 4B

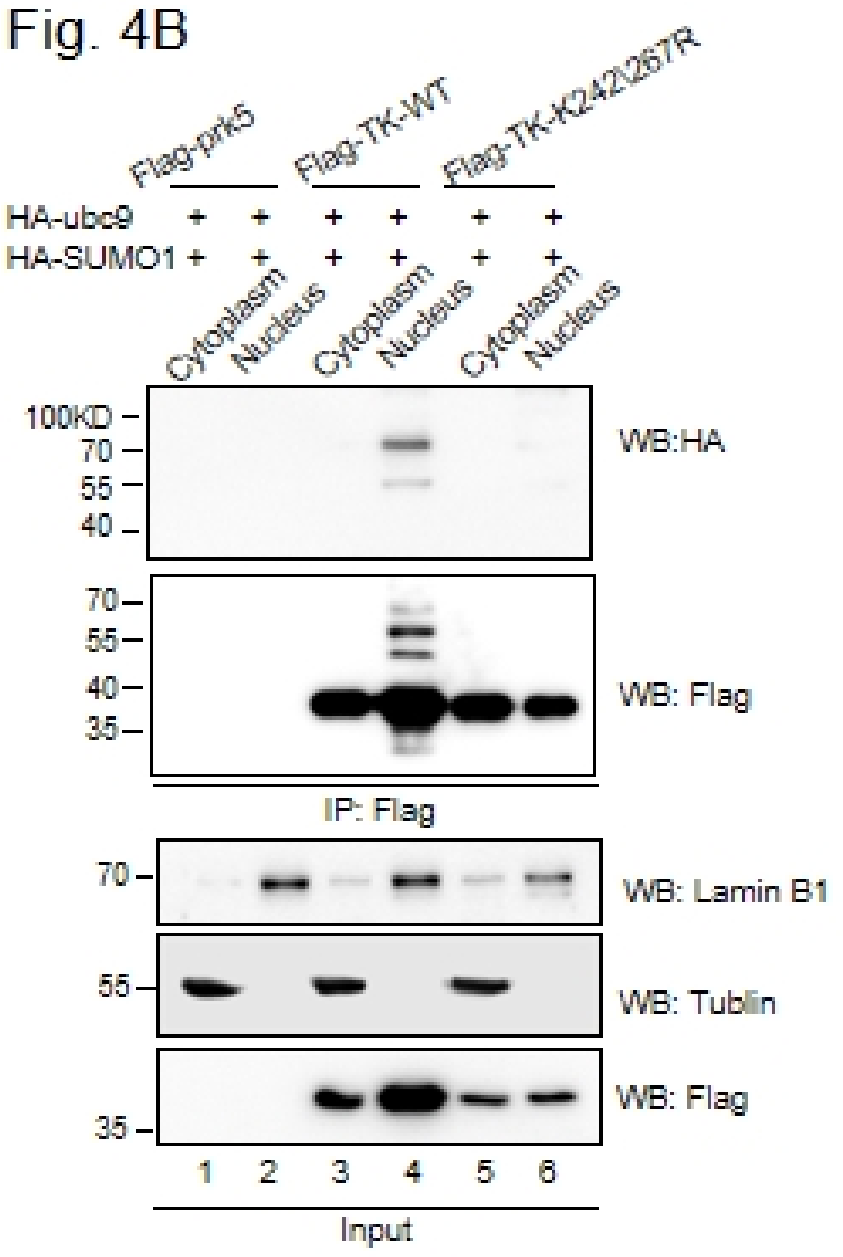

3. While Figure 7B uses PCR and Western Blot to validate viral rescue, it does not confirm the precise genetic sequence of the engineered viruses. The authors must provide Sanger sequencing chromatograms or Next-Generation Sequencing (NGS) alignments to verify the integrity of the TK expression cassette and the specific 4KR mutations within the viral genome.

To verify the integrity of the engineered viruses, we sequenced the entire modified genomic region, including the TK expression cassette and both insertion junctions, in rPRV-TK-WT and rPRV-TK-4KR. The sequencing results confirmed correct insertion into the intended Us3 locus and verified the presence of the designed 4KR mutations, with no unintended sequence changes detected within the engineered region. These data have now been included as Supplementary Fig. S1.

Fig. S1

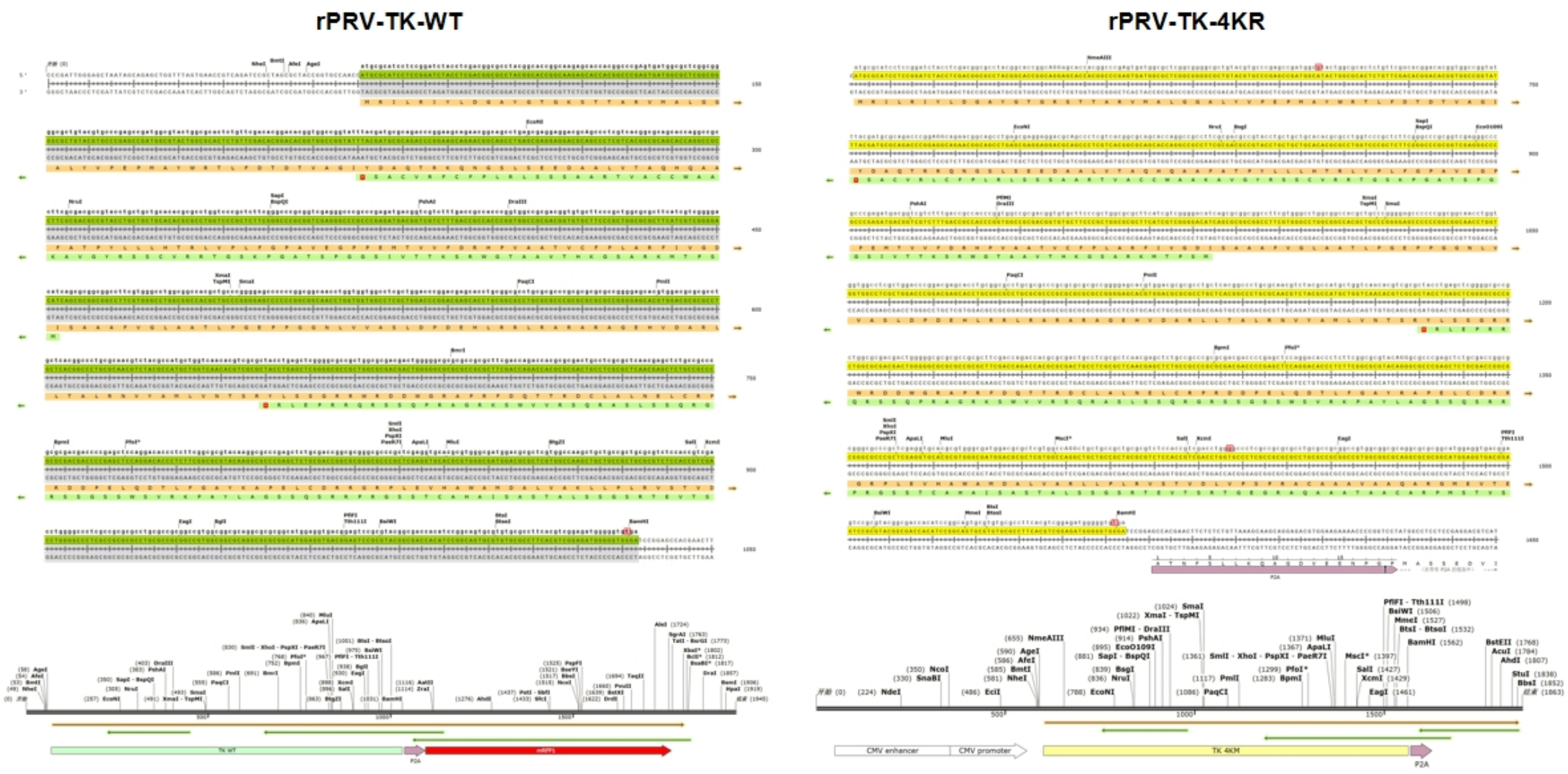

4. The authors chose the Us3 locus for TK insertion, which resulted in a significantly attenuated backbone compared to the parental PRV-WT. The authors must provide a clearer scientific defense for this choice, addressing how Us3 disruption impacts the baseline neurovirulence observed in the rPRV-TK-WT group. It is crucial to prove that the observed neuronal replication defects are solely due to TK SUMOylation and not an artifact of disrupting Us3 functions.

We thank the reviewer for this insightful comment. Actually, we initially attempted to introduce TK-WT or TK-4KR directly into the endogenous TK locus using our CRISPR/Cas9-based strategy. However, despite multiple independent attempts, we were unable to recover the desired recombinant viruses. Although the exact reason remains unclear, this may reflect the relatively low efficiency of homologous recombination at the endogenous TK locus or technical limitations associated with repeated editing of this genomic region.

Therefore, we adopted the Us3 locus as the insertion site, which has been routinely used in our previous studies because disruption of Us3 does not significantly affect viral replication in cultured cells. We fully acknowledge, however, that Us3 is an established neurovirulence factor and the contribution of Us3 disruption should be experimentally addressed.

To directly address this concern, we repeated the *in vivo* infection experiments by increasing the number of mice to six animals per group and including two additional control viruses, PRV-US3-KO and PRV-TK/US3-KO, in the revised study (revised Figure 8). The results show that Mice infected with PRV-WT developed severe pruritus and scratching behavior by 4 days post-infection (dpi), and all animals succumbed to infection within 6 dpi (revised Fig. 8A). Consistent with the established role of Us3 in PRV neurovirulence, PRV-US3-KO-infected mice exhibited similar clinical signs but showed delayed disease progression, with 100% mortality occurring by 7 dpi.

Importantly, despite sharing the same Us3-null background, rPRV-TK-WT and rPRV-TK-4KR displayed markedly different phenotypes *in vivo*. While both PRV-WT and PRV-US3-KO caused 100% mortality, rPRV-TK-WT showed a reduced mortality rate (80%) and delayed disease progression. More importantly, rPRV-TK-4KR exhibited complete survival (0% mortality), representing a significant attenuation compared with rPRV-TK-WT. This difference was further supported by body weight changes (revised Fig. 8B) , viral genome loads in tissues (revised Fig. 8C) , and histopathological analyses (revised Fig. 8D) , all of which consistently demonstrated reduced pathogenicity of the TK SUMOylation-deficient virus. Therefore, although Us3 deletion contributes to the overall attenuation of the recombinant virus backbone, the direct comparison between rPRV-TK-WT and rPRV-TK-4KR indicates that loss of TK SUMOylation confers an additional and significant reduction in viral virulence.

We also noted that rPRV-TK-WT and PRV-US3-KO were not completely identical in terms of mortality and body weight loss. One possible explanation is that insertion of the TK expression cassette into the Us3 locus may alter the timing and/or expression level of TK during the viral replication cycle compared with the native genomic context. We have included this point in the revised Results (lines 378-382) and Discussion (lines 536-551) as a potential limitation and interpretation of the recombinant virus phenotype.

Fig.8

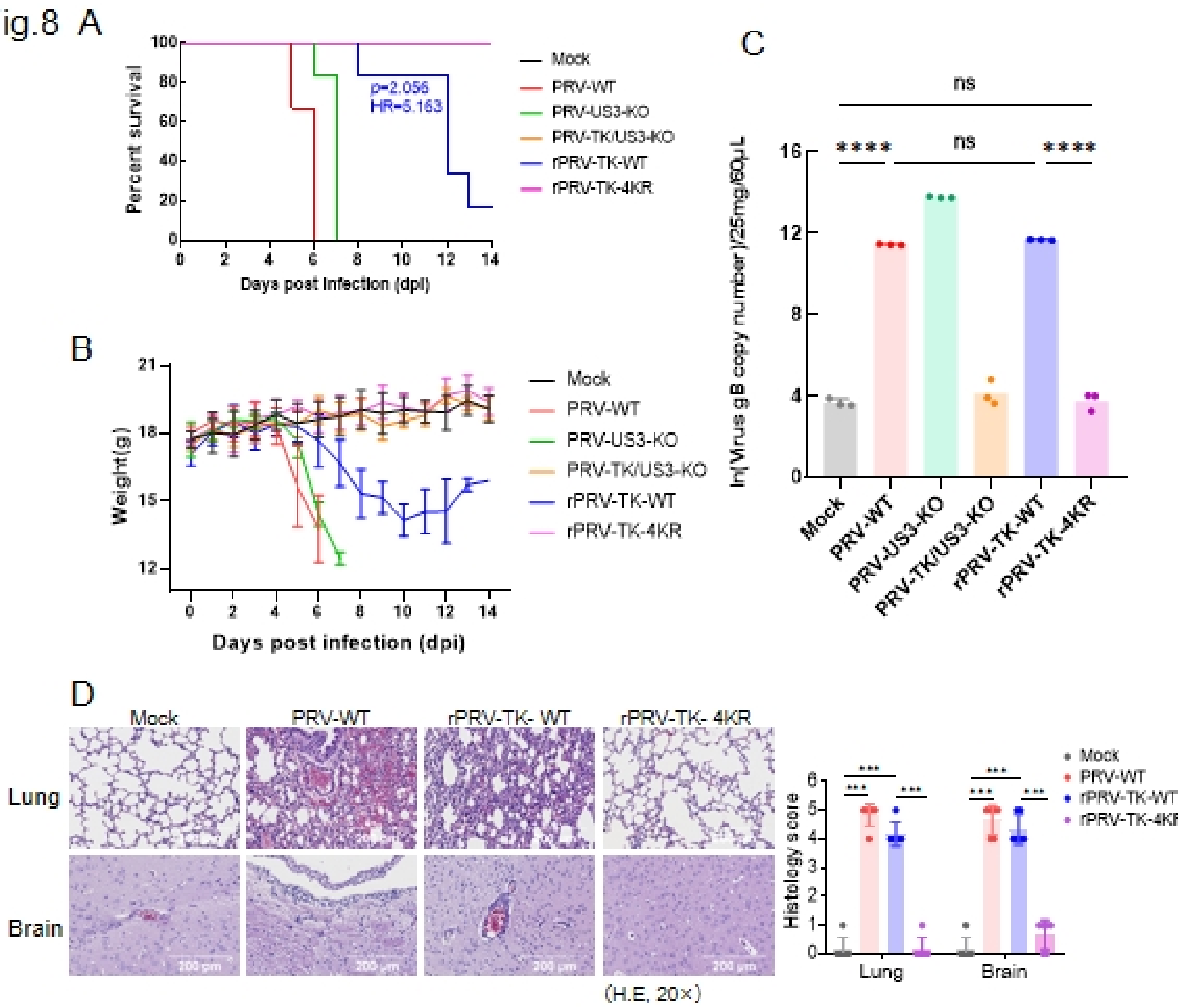

5. The authors describe a temperature-shift model in N2a cells. The authors should clarify if this specific protocol was developed in-house or adapted from existing literature, adding proper citations to the Materials and Methods section. Further explanation is needed on why 42℃ is sufficient to induce a latent-like state without compromising cell viability.

The N2a cell latency-like/reactivation model used in this study was adapted from previously published protocols (Refs. 35). We have now cited these studies in the Materials and Methods section and clarified that our experimental procedure was established based on these published methods.

As reported in these previous studies, incubation at 42 °C reversibly suppresses productive PRV replication in N2a cells without causing substantial cytotoxicity, thereby allowing establishment of a latency-like state that can be reactivated by returning the cultures to 37 °C. In the present study, we further validated this model by demonstrating that, after 3 days at 42 °C, viral genome replication, viral gene transcription, viral protein expression, and infectious virus production were all markedly suppressed, whereas these parameters recovered following temperature shift in reactivation-competent viruses (revised Fig. 7 and Fig. S2–S3).

To avoid overstating the characteristics of this experimental system, we have also revised the Results (lines 310-362) and Discussion (lines 521-535, 561-567) accordingly to avoid

overstating the nature of this model and now consistently refer to it as an *in vitro* latency-like/reactivation model.

6. The study suggests that SUMOylation facilitates nuclear accumulation via recruitment of host factors with SUMO-interacting motifs (SIMs). To strengthen the mechanistic chain, the authors are encouraged to identify or discuss potential SIM-containing host partners (e.g., specific importins or nuclear pore components).

As suggested, we have expanded the Discussion (lines 473-502) to include several plausible candidates that may mediate SUMO-dependent nuclear trafficking of TK. Specifically, we now discuss the possible involvement of members of the importin (karyopherin) family, such as importin-α/β, as well as components of the nuclear pore complex (nucleoporins), which have been implicated in SUMO-regulated nucleocytoplasmic transport. We emphasize that these proteins are proposed as candidate host factors based on previous studies of SUMO-dependent nuclear transport rather than direct experimental evidence in the present study.

We have also clarified that identification of the specific SIM-containing host proteins responsible for TK nuclear accumulation remains an important subject for future investigation.

7. In the mouse model, the authors should use immunohistochemistry (IHC) to track the viral spread within the CNS. This would clarify if the attenuation of the 4KR mutant is due to a failure to reach the brain or an inability to replicate efficiently once it arrives.

We agree that immunohistochemical analysis of viral antigen distribution in the CNS would provide additional information regarding viral dissemination during infection.

During revision, we repeated the mouse infection experiments using an increased number of animals (n = 6 per group) and attempted to detect viral antigen in brain and lung tissues by immunohistochemistry. Although we attempted to detect TK antigen in brain sections, the anti-TK antibody that performs well in Western blotting and immunofluorescence did not generate sufficiently robust and reproducible staining in paraffin-embedded tissues (see the following figures), preventing reliable evaluation of viral distribution. Therefore, we did not include these data in the revised manuscript.

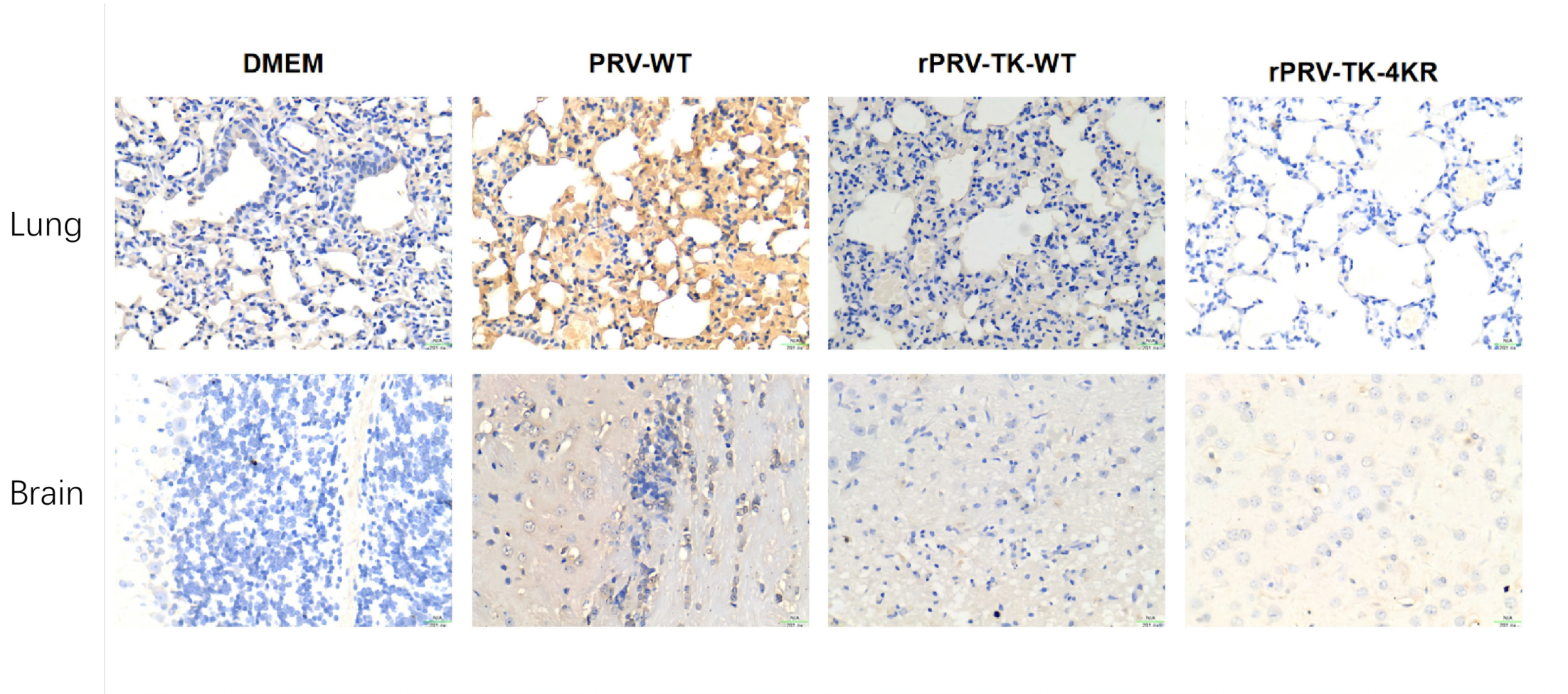

Nevertheless, the repeated animal experiments consistently demonstrated that the rPRV-TK-4KR mutant exhibited markedly reduced mortality, delayed weight loss, significantly lower viral DNA copy numbers in brain tissues, and substantially attenuated histopathological lesions compared with rPRV-TK-WT. Together, these data support the conclusion that loss of TK SUMOylation markedly impairs viral infection within the CNS.

We agree that distinguishing whether this attenuation primarily reflects impaired neuroinvasion or reduced replication after CNS entry is an important mechanistic question. We have therefore added this point to the Discussion (lines 567-573) and consider it an important direction for future investigation.

8. The comparison with HSV-1 TK is useful. Expanding this analysis via multiple sequence alignment with other veterinary alphaherpesviruses (e.g., BHV-1, EHV-1, MDV) would demonstrate whether the K242/K267 regulatory axis is a conserved evolutionary strategy.

We thank the reviewer for this valuable suggestion. To address this point, we performed a multiple sequence alignment of TK proteins from several representative alphaherpesviruses, including PRV, HSV-1, bovine herpesvirus 1 (BHV-1), equine herpesvirus 1 (EHV-1), and Marek's disease virus (MDV). These new data have been included as Supplementary Fig. S4 and are discussed in the revised Discussion (lines 453 – 468).

The sequence comparison showed that the predicted SUMOylation-associated lysine residues are partially conserved among alphaherpesviruses. Specifically, K16 is completely conserved, whereas K242 is replaced by arginine only in HSV-1, and K267 is replaced only in BHV-1. In contrast, K56 is considerably less conserved and is retained only in PRV and EHV-1. Among the viruses analyzed, PRV and EHV-1 exhibit the highest conservation of all four lysine residues.

These findings suggest that the regulatory mechanism mediated by TK SUMOylation may be at least partially conserved during alphaherpesvirus evolution, while species-specific divergence may also exist. Notably, the reduced SUMOylation observed for HSV-1 TK in

our supplementary experiments (revised Fig. S4B) may be related to the replacement of K56 and K242 by arginine, although this possibility requires further experimental validation.

Because HSV-1 is not the primary focus of the present study, and we did not generate recombinant HSV-1 mutants or perform corresponding functional analyses, we have moved the HSV-1-related experiments from the main figures to Supplementary Fig. S4 to better maintain the focus of the manuscript while preserving these comparative evolutionary data.

Fig. S4

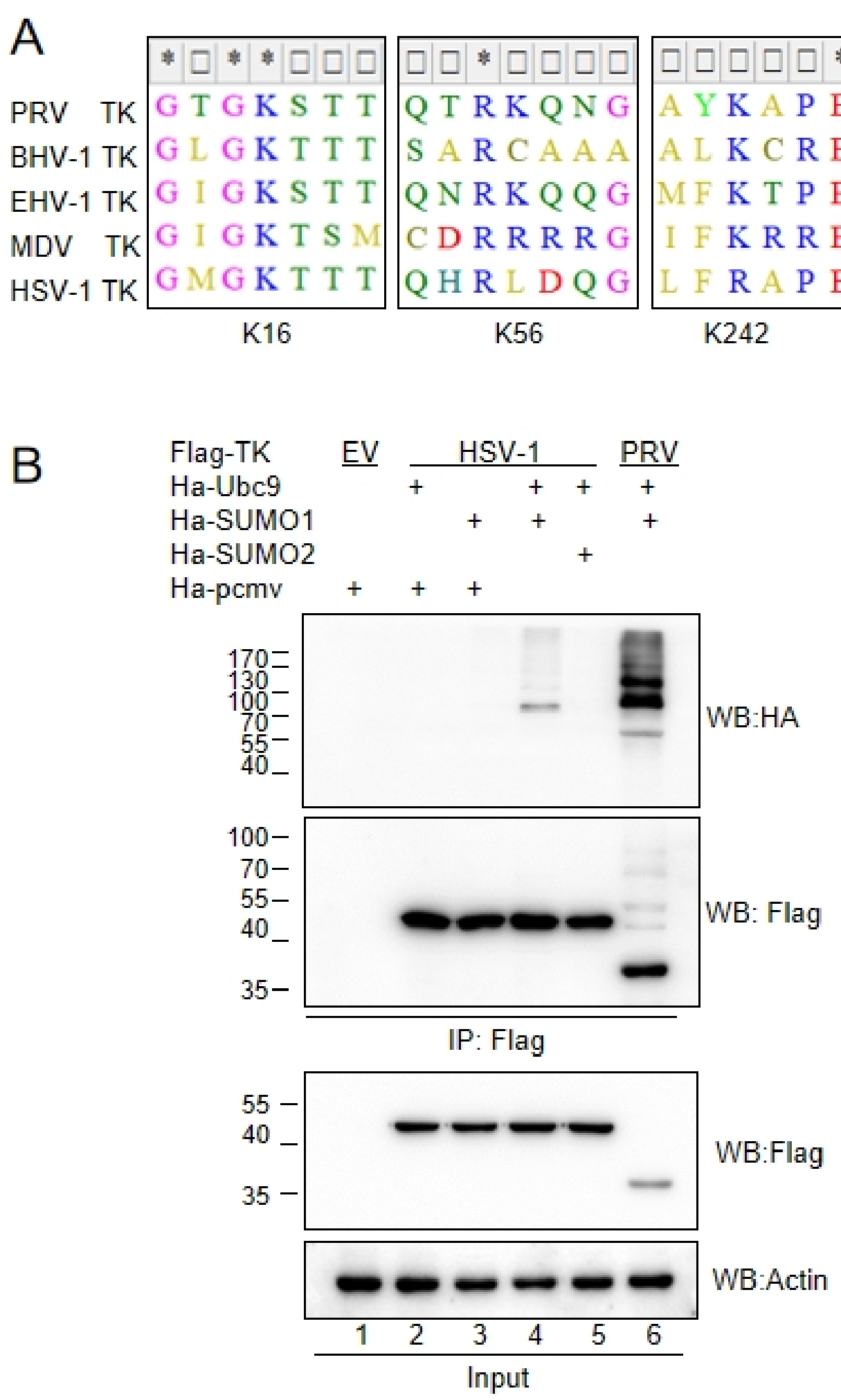

Supplement: Supplementary file 2 [file ppat.1014653.s008.pdf]
