# Supplementary material for: SUMOylation of pseudorabies virus thymidine kinase regulates its nuclear localization and facilitates neuronal replication and reactivation
Source: PLoS Pathog. 2026 Oct 5;22(10):e1014653. doi: 10.1371/journal.ppat.1014653 (PMC13649132; doi:10.1371/journal.ppat.1014653)
Supplement: Peer review history

# Peer review: Decision Letter 0

**DOI:** 10.1371/journal.ppat.1014653.r001

**Authors:** Pinghui Feng, Alison McBride

12 May 2026

PPATHOGENS-D-26-00941

SUMOylation of Pseudorabies Virus Thymidine Kinase Controls Its Nuclear Localization and Promotes Neuronal Replication and Reactivation

PLOS Pathogens

Dear Dr. Zhang,

Thank you for submitting your manuscript to PLOS Pathogens. After careful consideration, we feel that it has merit but does not fully meet PLOS Pathogens's publication criteria as it currently stands. Therefore, we invite you to submit a revised version of the manuscript that addresses the points raised during the review process.

Please submit your revised manuscript by July 12. If you will need more time than this to complete your revisions, please reply to this message or contact the journal office at plospathogens@plos.org. When you're ready to submit your revision, log on to https://www.editorialmanager.com/ppathogens/ and select the 'Submissions Needing Revision' folder to locate your manuscript file.

Please include the following items when submitting your revised manuscript:

* A letter that responds to each point raised by the editor and reviewer(s). You should upload this letter as a separate file labeled 'Response to Reviewers'. This file does not need to include responses to any formatting updates and technical items listed in the 'Journal Requirements' section below.

* A marked-up copy of your manuscript that highlights changes made to the original version. You should upload this as a separate file labeled 'Revised Manuscript with Track Changes'.

* An unmarked version of your revised paper without tracked changes. You should upload this as a separate file labeled 'Manuscript'.

If you would like to make changes to your financial disclosure, competing interests statement, or data availability statement, please make these updates within the submission form at the time of resubmission. Guidelines for resubmitting your figure files are available below the reviewer comments at the end of this letter. As the corresponding author, your ORCID iD is verified in the submission system and will appear in the published article. PLOS supports the use of ORCID, and we encourage all coauthors to register for an ORCID iD and use it as well. Please encourage your coauthors to verify their ORCID iD within the submission system before final acceptance, as unverified ORCID iDs will not appear in the published article. Only the individual author can complete the verification step; PLOS staff cannot verify ORCID iDs on behalf of authors.

We look forward to receiving your revised manuscript.

Kind regards,

Pinghui Feng

Academic Editor

PLOS Pathogens

Alison McBride

Section Editor

PLOS Pathogens Sumita Bhaduri-McIntosh

Editor-in-Chief

PLOS Pathogens

orcid.org/0000-0003-2946-9497 Michael Malim

Editor-in-Chief

PLOS Pathogens

orcid.org/0000-0002-7699-2064

**Journal Requirements:**

1) Please ensure that the CRediT author contributions listed for every co-author are completed accurately and in full.

At this stage, the following Authors/Authors require contributions: Xuan Chen, Chuang Li, Jingjing Song, Yun Liu, Dongyue Xing, Xian Li, Jinlei Zhang, Yuqi Zhang, Nianzhi Zhang, Rulan Bai, Changyong Cheng, Jun Tang, and Rui Zhang. Please ensure that the full contributions of each author are acknowledged in the "Add/Edit/Remove Authors" section of our submission form.

The list of CRediT author contributions may be found here: https://journals.plos.org/plospathogens/s/authorship#loc-author-contributions

2) We ask that a manuscript source file is provided at Revision. Please upload your manuscript file as a .doc, .docx, .rtf or .tex. If you are providing a .tex file, please upload it under the item type u2018LaTeX Source Fileu2019 and leave your .pdf version as the item type u2018Manuscriptu2019.

3) Please upload all main figures as separate Figure files in .tif or .eps format. For more information about how to convert and format your figure files please see our guidelines:

https://journals.plos.org/plospathogens/s/figures

4) We notice that your supplementary Figures are included in the manuscript file. Please remove them and upload them with the file type 'Supporting Information'. Please ensure that each Supporting Information file has a legend listed in the manuscript after the references list.

5) In the online submission form, you indicated that Additional raw datasets, plasmids, and recombinant PRV strains generated during this work are available from the corresponding author upon reasonable request.. All PLOS journals now require all data underlying the findings described in their manuscript to be freely available to other researchers, either

1. In a public repository

2. Within the manuscript itself

3. Uploaded as supplementary information.

This policy applies to all data except where public deposition would breach compliance with the protocol approved by your research ethics board. If your data cannot be made publicly available for ethical or legal reasons (e.g., public availability would compromise patient privacy), please explain your reasons by return email and your exemption request will be escalated to the editor for approval. Your exemption request will be handled independently and will not hold up the peer review process, but will need to be resolved should your manuscript be accepted for publication. One of the Editorial team will then be in touch if there are any issues.

6) Please amend your detailed Financial Disclosure statement. This is published with the article. It must therefore be completed in full sentences and contain the exact wording you wish to be published.

1) State the initials, alongside each funding source, of each author to receive each grant. For example: "This work was supported by the National Institutes of Health (####### to AM; ###### to CJ) and the National Science Foundation (###### to AM)."

2) State what role the funders took in the study. If the funders had no role in your study, please state: "The funders had no role in study design, data collection and analysis, decision to publish, or preparation of the manuscript."

7) Please ensure that the funders and grant numbers match between the Financial Disclosure field and the Funding Information tab in your submission form. Note that the funders must be provided in the same order in both places as well. Currently,  2115 Talent Development Program 623of China Agricultural University" is missing from the Funding Information tab.

Note:If the reviewer comments include a recommendation to cite specific previously published works, please review and evaluate these publications to determine whether they are relevant and should be cited. There is no requirement to cite these works unless the editor has indicated otherwise.

**Reviewers' Comments:**

Reviewer's Responses to Questions

**Part I - Summary**

Please use this section to discuss strengths/weaknesses of study, novelty/significance, general execution and scholarship.

Reviewer #1: Chen and coauthors identified the critical biological function of viral TK SUMOylation in PRV infection. SUMOylation is essential for TK trafficking to nuclear and subsequent viral replication and reactivation. The modification site(s) on TK were determined and validated. SUMOylation-resistant mutant viruses lost the ability of nuclear localization and efficient viral replication in cells and in vivo. The finding is innovative and provides a valuable reference for designing antiviral defense. The study is within the scope of this journal. However, it could be strengthened by addressing the following concerns.

Reviewer #2: Summary

This manuscript identifies SUMOylation of PRV thymidine kinase (TK) at K242/K267 and claims that this modification promotes TK nuclear localization, neuronal replication, reactivation from latency, and in vivo virulence. While the study is extensive and addresses an interesting topic, several critical issues undermine the current conclusions.

Reviewer #3: This manuscript, “SUMOylation of Pseudorabies Virus Thymidine Kinase Controls Its Nuclear Localization and Promotes Neuronal Replication and Reactivation,” by Chen et al, investigates the post-translational regulation of PRV thymidine kinase (TK) by SUMOylation and proposes that SUMO modification controls TK nuclear localization, neuronal replication, reactivation from latency, and viral pathogenicity. The study addresses an interesting and important question, particularly given the known importance of TK in alphaherpesvirus neurotropism and latency/reactivation biology. The authors present biochemical, cell biological, recombinant virus, neuronal replication, and in vivo infection data suggesting that TK is SUMO-modified and that mutation of SUMOylation sites attenuates neuronal infection and virulence. While the manuscript contains several interesting observations, the mechanistic conclusions are currently overstated relative to the evidence presented. The central claim, that direct SUMOylation of TK at K242 and K267 regulates nuclear localization and controls neuronal replication/reactivation, is not conclusively demonstrated. The manuscript lacks critical mechanistic experiments demonstrating endogenous TK SUMOylation during infection, preservation of TK enzymatic activity in the 4KR mutant, and definitive evidence linking the neuronal and in vivo phenotypes specifically to SUMOylation rather than broader functional disruption of TK.

Reviewer #4: This study identifies Pseudorabies virus (PRV) Thymidine Kinase (TK) as a novel substrate for SUMOylation at residues K242 and K267. The authors demonstrate that this post-translational modification is essential for the nuclear localization of TK. While dispensable in epithelial cells (PK15), TK SUMOylation is shown to be critical for viral replication in neuronal cells (N2a), reactivation from latency, and overall neurovirulence in a mouse model. The discovery of a host-mediated PTM regulating a key viral metabolic enzyme’s trafficking provides significant insights into alphaherpesvirus neurotropism. However, several mechanistic details and experimental controls require strengthening to meet the high standards of PLOS Pathogens.

**********

**Part II – Major Issues: Key Experiments Required for Acceptance**

Please use this section to detail the key new experiments or modifications of existing experiments that should be absolutely required to validate study conclusions.

Generally, there should be no more than 3 such required experiments or major modifications for a "Major Revision" recommendation. If more than 3 experiments are necessary to validate the study conclusions, then you are encouraged to recommend "Reject".

Reviewer #1: TK is the substrate of all SUMO1,2,3, and SUMO3 shows a much stronger TK SUMOylation in Figure 1D. So, why choose SUMO1, but not SUMO3 for the following studies, and antiviral target?

Would knocking down SUMO1, 2, and 3 abolish TK SUMOylation? And prevent viral replication or reactivation? And the following question is what’s the status of TK and mutations SUMOylation by endogenous SUMO1, 2, and 3?

SUMO-conjugating enzyme Ubc9 was co-transfected with SUMO1 in the assay, but SUMOylation is still occurring without external Ubc9 expression (Fig.1D), so why using the articulated conditions by introducing overexpressed Ubc9? Please include conditions without overexpressing Ubc9.

In the PK15 cell, SUMOylation has no effect on viral replication (Fig. 7D, 7E), while N2a has a much stronger phenotype. So, would it be valuable to show results in N2a cells rather than PK15 cells? Since there is no phenotype (viral growth curve) in PK15, how to interpret or explain the results in Figure 6? Would that be the same result when using N2a? Please include the data using N2a cells in Figure 6.

The section on HSV-1 is a bit out of context in this manuscript, as the main focus is PRV-related studies. It is interesting that HSV-1 TK is the substrate of SUMO1, not SUMO2. However, it is better to show this section at the end with more evidence of TK SUMOylation in various alpha-herpes. Alternatively, include more results of recombinant HSV-1 in cells and in vivo studies in the following section.

Reviewer #2: Major Issues: Key Experiments Required for Acceptance

1. All SUMOylation experiments were performed using overexpression systems. The authors should provide evidence that endogenous SUMOylation of TK and its mutants occurs in the context of genuine viral infection. It remains unclear whether the observed SUMOylation is a physiologically relevant event during PRV replication rather than an artifact of transient transfection.

2.The in vivo virulence conclusion is confounded by disruption of the Us3 Locus. The recombinant viruses (rPRV-TK-WT and rPRV-TK-4KR) were constructed by inserting the TK expression cassette into the Us3 gene, thereby inactivating Us3, a well established neurovirulence factor. Because rPRV-TK-WT (Us3 null) already exhibits marked attenuation compared to parental PRV WT, the additional loss of TK SUMOylation in rPRV-TK-4KR cannot be ascribed to reduced pathogenicity. The mouse experiment lacks the essential control – direct comparison between rPRV-TK-WT and rPRV-TK-4KR to support the claim that TK SUMOylation per se contributes to virulence.

3. The proposed reactivation defect may simply reflect a general replication defect of the SUMOylation‑deficient virus. The authors report that rPRV‑TK‑4KR fails to reactivate in N2a cells, but they do not compare the latent viral genome loads established prior to reactivation between rPRV‑TK‑WT and rPRV‑TK‑4KR. Without evidence that equal amounts of latent genomes are present before the temperature shift, the observed failure to reactivate could be entirely explained by reduced replication capacity rather than a specific block in the latency‑reactivation switch.

Reviewer #3: 1. The SUMOylation assays rely on native immunoprecipitation conditions using overexpressed HA-SUMO and Ubc9. Under these conditions, the HA-reactive high-molecular-weight band could be from SUMOylated interacting proteins.

2. The mutagenesis data suggest that K242 and K267 contribute to SUMO-associated high-molecular-weight species, but the data do not conclusively establish these residues as direct SUMO acceptor sites, as neither single nor combined mutations completely abolish HA-reactive bands, and the 4KR mutant still retains residual SUMO-associated signals.

3. TK is a critical viral kinase required for nucleotide metabolism and efficient replication in neurons and non-dividing cells. The observed defects in neuronal replication, reactivation, and virulence could therefore result from impaired TK catalytic function rather than altered SUMOylation. Without demonstrating that the mutant retains normal TK function aside from SUMOylation deficiency, the mechanistic interpretation remains unclear.

4. The SUMO-deficient mutants exhibit puncta-like localization patterns, which could represent aggregation or the formation of inclusion bodies, making TK sequestered away from the replication foci, thus leading to the phenotype.

5. All SUMOylation experiments are performed using transient overexpression systems and does not demonstrate that endogenous TK becomes SUMOylated during authentic PRV infection, which is a major concern.

6. The 42°C “latency/reactivation” model in N2a cells is not rigorously established as a bona fide latency system. Elevated temperature may induce restricted replication. The manuscript does not show latency via episomal persistence or chromatinized viral genomes, which could impact “reactivation defects” observed in recombinant virus.

7. The recombinant viruses are insufficiently characterized, such as full-genome sequencing, which is particularly important because the recombinant WT virus itself appears partially attenuated relative to parental PRV-WT.

8. The mouse infection experiments use only n = 3 animals per group, which is insufficient for robust conclusions regarding virulence and pathogenesis. Additionally, the histopathology is descriptive, and the survival statistics are absent.

9. Throughout the manuscript, the language often implies definitive mechanistic causality that does not seem to be fully supported. For example, SUMOylation controls TK nuclear localization, and SUMOylation promotes reactivation. SUMOylation regulates virulence” should be moderated unless stronger mechanistic evidence is provided.

Reviewer #4: 1. The Figure 4A shows only a single representative cell per condition. This is insufficient to demonstrate the consistency of the phenotype across the cell population; the authors should provide images with a larger field of view (FOV) showing multiple cells. A formal statistical analysis is required to quantify the percentage of cells exhibiting "Nuclear," "Cytoplasmic," or "Both" localization patterns.

2. In figure 4B, the authors use Tubulin as a cytoplasmic marker to verify fractionation, however, a specific nuclear marker (e.g., Lamin B1 or Histone H3) is mandatory to demonstrate the purity of the nuclear fraction and the absence of cross-contamination . Furthermore, to objectively confirm that SUMOylation deficiency increases cytoplasmic distribution.

3. While Figure 7B uses PCR and Western Blot to validate viral rescue, it does not confirm the precise genetic sequence of the engineered viruses. The authors must provide Sanger sequencing chromatograms or Next-Generation Sequencing (NGS) alignments to verify the integrity of the TK expression cassette and the specific 4KR mutations within the viral genome.

4. The authors chose the Us3 locus for TK insertion, which resulted in a significantly attenuated backbone compared to the parental PRV-WT. The authors must provide a clearer scientific defense for this choice, addressing how Us3 disruption impacts the baseline neurovirulence observed in the rPRV-TK-WT group. It is crucial to prove that the observed neuronal replication defects are solely due to TK SUMOylation and not an artifact of disrupting Us3 functions.

5. The authors describe a temperature-shift model in N2a cells. The authors should clarify if this specific protocol was developed in-house or adapted from existing literature, adding proper citations to the Materials and Methods section. Further explanation is needed on why 42℃ is sufficient to induce a latent-like state without compromising cell viability.

6. The study suggests that SUMOylation facilitates nuclear accumulation via recruitment of host factors with SUMO-interacting motifs (SIMs). To strengthen the mechanistic chain, the authors are encouraged to identify or discuss potential SIM-containing host partners (e.g., specific importins or nuclear pore components).

7. In the mouse model, the authors should use immunohistochemistry (IHC) to track the viral spread within the CNS. This would clarify if the attenuation of the 4KR mutant is due to a failure to reach the brain or an inability to replicate efficiently once it arrives.

8. The comparison with HSV-1 TK is useful. Expanding this analysis via multiple sequence alignment with other veterinary alphaherpesviruses (e.g., BHV-1, EHV-1, MDV) would demonstrate whether the K242/K267 regulatory axis is a conserved evolutionary strategy.

**********

**Part III – Minor Issues: Editorial and Data Presentation Modifications**

Please use this section for editorial suggestions as well as relatively minor modifications of existing data that would enhance clarity.

Reviewer #1: When performing the SUMOylation assay, what’s the rationale for only screening Us1, Us3, UL13, TK, or UL48? What’s the selection criteria? By Mass spectrometry using infected cells? Structure analysis? Please include more details and clarification.

Figure 1C, 1D, please indicate the size of the protein band through out the figures. Some WB blots show, but some do not.

Please align the symbols + and – correctly in each WB panel, e.g. Fig1D.

Figure 2D, please provide the structure model ID used for PyMOL modeling.

To examine the biological role of TK SUMOylation, it flows better with the protein stability, localization and deSUMOylating affecting localization.

Figure 7C, how many images were used for quantification? Please show the standard deviation.

The Figure was not correctly called out in the manuscript. Please correct the Figure numbers. Figure 9 is missing.

The H&E staining of infected tissue was not sufficient to show the viral pathogenesis. Please provide staining evidence of viral proteins (TK is preferred), cell markers and SUMO1.

Lack of experimental details. Details of CRISPR/Cas9 system, concentrations of primary and secondary antibody in the methods, should be provided for data reproducibility.

What’s the qPCR condition? Performed on which instrument?

Animal experimental procedures, for example, how tissue was processed for viral titration and other analysis?

Please indicate the number of mice used in each group in the figure legends and in the methods. How is the number of animals determined?

Line 515, add a space before Briefly.

Reviewer #2: Minor Issues: Editorial and Data Presentation Modifications

1. PRV is a large DNA virus encoding 70–100 proteins. Why were only Us1, Us3, UL13, TK, and UL48 examined for SUMOylation? It is not clear whether other PRV-encoded proteins are also SUMOylated. The authors should at least discuss this limitation.

2. In the co immunoprecipitation experiments, the input blots do not show HA-SUMO1 expression. Please include HA SUMO1 input controls to demonstrate equal expression of the SUMO construct across samples.

3. All SUMOylation evidence is derived from overexpression of Flag‑TK and HA‑SUMO. The authors need to determine whether endogenous SUMOylation of TK occurs during actual PRV infection.

4. The conclusion that K242 and K267 are essential for SUMO‑1 conjugation would be strengthened by constructing a recombinant PRV carrying the TK‑K242/267R mutation and testing its SUMOylation in the context of infection. The authors should consider this or at least justify why it was not done.

5. Figure 3 (HSV-1 TK SUMOylation) is peripheral to the main story. The data could be moved to supplementary material or merged into Figure 2 as an extension; it does not warrant a separate main figure.

6. In the nuclear cytoplasmic fractionation experiment (Fig. 4B), the input panels lack nuclear marker controls. Please add these markers to validate fraction purity. Moreover, as in point #4, a recombinant PRV-TK-K242/267R would provide stronger evidence that SUMOylation drives nuclear localization during infection.

7. Figure 5A shows that TK‑K242/267R appears to degrade faster than TK‑WT upon CHX chase. Additionally, in Figure 5B input, Flag‑TK‑K242/267R and Flag‑TK‑4KR levels are visibly lower than Flag‑TK‑WT. These observations contradict the authors’ statement that SUMOylation does not affect TK stability. Please provide an explanation – e.g., quantification with

8. Figure 3B: The sequence alignment between PRV and HSV‑1 TK is used to explain lower SUMOylation efficiency of HSV‑1 TK. However, the two proteins share low overall similarity, and the lack of corresponding lysines is only one possible explanation. This speculation would be more appropriate in the Discussion rather than presented as a definitive result.

9. The manuscript would benefit from professional language editing to improve readability, correct minor grammatical errors, and remove over statements (e.g., “unprecedented”, “first report”).

Reviewer #3: 1. Many immunoblots are overexposed and contain diffuse smears that are difficult to interpret quantitatively.

2. Figures lack densitometric quantification and statistical analysis.

3. NEM or SUMO protease inhibitors during lysis are not described and should be clarified.

4. Scale bars are missing from several microscopy panels.

5. Confocal imaging would improve the interpretation of localization phenotypes.

6. The manuscript should include quantitative nuclear/cytoplasmic localization analysis with replicate statistics.

7. Fractionation experiments require additional nuclear and cytoplasmic purity markers.

8. Figure legends contain occasional formatting inconsistencies and duplicated text.

9. Statistical methods require a more detailed description, including biological vs technical replicates and n values.

10. The nomenclature of mutants and recombinant viruses should be standardized throughout the manuscript.

11. Additional neuronal models or primary neurons would substantially strengthen the biological relevance.

12. The manuscript would benefit from a discussion of whether SUMOylation affects TK interaction with known viral or cellular partners.

Reviewer #4: none

**********

PLOS authors have the option to publish the peer review history of their article (what does this mean?). If published, this will include your full peer review and any attached files.

If you choose “no”, your identity will remain anonymous but your review may still be made public.

**Do you want your identity to be public for this peer review?**  For information about this choice, including consent withdrawal, please see our Privacy Policy.

Reviewer #1: No

Reviewer #2: No

Reviewer #3: No

Reviewer #4: No

[NOTE: If reviewer comments were submitted as an attachment file, they will be attached to this email and accessible via the submission site. Please log into your account, locate the manuscript record, and check for the action link "View Attachments". If this link does not appear, there are no attachment files.]

**Figure resubmission:**

While revising your submission, we strongly recommend that you use PLOS’s NAAS tool (https://ngplosjournals.pagemajik.ai/artanalysis) to test your figure files. NAAS can convert your figure files to the TIFF file type and meet basic requirements (such as print size, resolution), or provide you with a report on issues that do not meet our requirements and that NAAS cannot fix. After uploading your figures to PLOS’s NAAS tool - https://ngplosjournals.pagemajik.ai/artanalysis, NAAS will process the files provided and display the results in the "Uploaded Files" section of the page as the processing is complete. If the uploaded figures meet our requirements (or NAAS is able to fix the files to meet our requirements), the figure will be marked as "fixed" above. If NAAS is unable to fix the files, a red "failed" label will appear above. When NAAS has confirmed that the figure files meet our requirements, please download the file via the download option, and include these NAAS processed figure files when submitting your revised manuscript.

**Reproducibility:**

To enhance the reproducibility of your results, we recommend that authors of applicable studies deposit laboratory protocols in protocols.io, where a protocol can be assigned its own identifier (DOI) such that it can be cited independently in the future. Additionally, PLOS ONE offers an option to publish peer-reviewed clinical study protocols. Read more information on sharing protocols at https://plos.org/protocols?utm_medium=editorial-email&utm_source=authorletters&utm_campaign=protocols

# Author comment: Author response to Decision Letter 1

**DOI:** 10.1371/journal.ppat.1014653.r002

21 Jul 2026

Attachment Submitted filename: Response to reviewers.pdf

# Peer review: Decision Letter 1

**DOI:** 10.1371/journal.ppat.1014653.r003

**Authors:** Pinghui Feng, Alison McBride

22 Sep 2026

Dear Dr. Zhang,

We are pleased to inform that your manuscript, "SUMOylation of Pseudorabies Virus Thymidine Kinase Regulates Its Nuclear Localization and Facilitates Neuronal Replication and Reactivation", has been editorially accepted for publication at PLOS Pathogens. However, Reviewer #2 raised some minor issues that can be addressed by editing your manuscript. Please do so and send a finalized version within a week.

Before your manuscript can be formally accepted and sent to production, you will need to complete our formatting changes, which you will receive by email within a week. Please note that your manuscript will not be scheduled for publication until you have made the required changes. Additionally, please also make changes on the points raised by reviewer #2, which can be done editing your manuscripts and fixing the errors without experiments.

IMPORTANT NOTES

(1) Copyediting and Proofreading: The corresponding author will receive a typeset proof for review, to ensure errors have not been introduced during production. Please review the PDF proof of your manuscript carefully, as this is the last chance to correct any errors. Please note that major changes, or those which affect the scientific understanding of the work, will likely cause delays to the publication date of your manuscript.

(2) Appropriate Figure Files: Please remove all name and figure # text from your figure files. Please also take this time to check that your figures are of high resolution, which will improve the readbility of your figures and help expedite your manuscript's publication. Please note that figures must have been originally created at 300dpi or higher. Do not manually increase the resolution of your files. For instructions on how to properly obtain high quality images, please review our Figure Guidelines, with examples at: http://journals.plos.org/plospathogens/s/figures.

We encourage you to use PACE (http://pace.apexcovantage.com/), a figure-checking tool which helps authors ensure all figures meet PLOS requirements so that figures can publish in the highest quality. PACE is a digital diagnostic and conversion tool for figure files. It will provide information about any failed check(s) and, if able, will automatically convert the figure into an acceptable file that passes quality checks.

To use PACE, you must first register as a user. Then, login and navigate to the UPLOAD tab, where you will find detailed instructions on how to use the tool.

(3) Striking Image: Please upload a striking still image to accompany your article if one is available (you can include a new image or an existing one from within your manuscript). Should your paper be accepted, this image will be considered for our monthly issue image and may also appear on our website to feature your article. Please upload this as a separate file, selecting "striking image" as the file type upon upload. Please also include a separate "Other" file with a caption, including credits and any potential copyright information. Please do not include the caption in the main article file. If your image is from someone other than yourself, please ensure that the artist has read and agreed to the terms and conditions of the Creative Commons Attribution License at http://journals.plos.org/plospathogens/s/content-license. Please note that PLOS cannot publish copyrighted images.

(4) Press Release or Related Media: If you or your institution will be preparing press materials for this manuscript, please inform our press team in advance at plospathogens@plos.org as soon as possible. We ask that you contact us within one week to plan ahead of our fast Production schedule. If you need to know your paper's publication date for related media purposes, you must coordinate with our press team, and your manuscript will remain under a strict press embargo until the publication date and time.

(5) Update your Profile Information: Now that your manuscript has been provisionally accepted, please log into Editorial Manager and update your profile, if needed. Go to http://www.editorialmanager.com/ppathogens, log in, and click on the "Update My Information" link at the top of the page. Please update your user information to ensure an efficient production and billing process.

(6) LaTeX users only: Our staff will ask you to upload a TEX file in addition to the PDF before the paper can be sent to typesetting, so please carefully review our Latex Guidelines http://journals.plos.org/plospathogens/s/latex in the meantime.

Thank you again for supporting Open Access publishing; we are looking forward to publishing your work in PLOS Pathogens.

Best regards,

Pinghui Feng

Academic Editor

PLOS Pathogens

Alison McBride

Section Editor

PLOS Pathogens

Dominique Soldati-Favre

Editor-in-Chief

PLOS Pathogens

***********************************************************

Reviewer's Responses to Questions

**Part I - Summary**

Please use this section to discuss strengths/weaknesses of study, novelty/significance, general execution and scholarship.<br/><br/>

Reviewer #1: The authors made significant improvements and addressed all the concerns.

Reviewer #2: Thank you for the opportunity to evaluate the revised manuscript entitled " SUMOylation of Pseudorabies Virus Thymidine Kinase Regulates Its Nuclear Localization and Facilitates Neuronal Replication and Reactivation" (PPATHOGENS-D-26-00941R1).

The central finding of this revised manuscript—that SUMOylation at K242/K267 directs TK nuclear accumulation and thereby supports neuronal replication and reactivation—is novel, well supported overall, and of clear interest to the readership of PLOS Pathogens. I have no major concerns. Provided the following minor points are addressed, I consider the manuscript acceptable for publication.

Reviewer #3: The authors have substantially addressed my major concerns through additional experiments and appropriate revisions to the manuscript. They have performed additional experiments examining endogenous TK SUMOylation during infection and preservation of TK kinase activity, and the expanded in vivo studies significantly strengthen the conclusions. The authors have also appropriately included the interpretation of the in vitro latency-like/reactivation model and acknowledged limitations of the recombinant virus system. Overall, I am satisfied with the revision and have no additional concerns.

Reviewer #4: I have carefully re-evaluated the revised manuscript and the authors' detailed responses. All of my previous concerns have been satisfactorily addressed. The authors have provided compelling additional data, including endogenous SUMOylation assays under denaturing conditions, Sanger sequencing verification of the recombinant viruses, quantitative subcellular localization analyses with statistical support, and expanded in vivo experiments with increased sample size and proper control viruses (PRV-US3-KO and PRV-TK/US3-KO). They have also clarified the rationale for using the Us3 insertion site, added multiple sequence alignment across alphaherpesviruses, and appropriately moderated overstatements throughout the text. The mechanistic conclusions are now well supported by the experimental evidence. I am satisfied with the revisions and recommend acceptance for publication in PLOS Pathogens.

--------------------

**Part II – Major Issues: Key Experiments Required for Acceptance**

Please use this section to detail the key new experiments or modifications of existing experiments that should be absolutely required to validate study conclusions.<br/><br/>Generally, there should be no more than 3 such required experiments or major modifications for a "Major Revision" recommendation. If more than 3 experiments are necessary to validate the study conclusions, then you are encouraged to recommend "Reject".<br/><br/>

Reviewer #1: (No Response)

Reviewer #2: The revised manuscript incorporates the additional experiments requested previously (Us3-locus control viruses, endogenous SUMOylation analyses, and sequence validation of the recombinant viruses), which considerably reinforce the main conclusions. I require no further key experiments. The outstanding points concern only editorial accuracy and the completeness of methodological descriptions, as detailed in the minor comments below.

Reviewer #3: None

Reviewer #4: None

--------------------

**Part III – Minor Issues: Editorial and Data Presentation Modifications**

Please use this section for editorial suggestions as well as relatively minor modifications of existing data that would enhance clarity.<br/><br/>

Reviewer #1: (No Response)

Reviewer #2: 1.Citation mismatch (Lines 311–313). The sentence "Previous studies have shown that TK is dispensable for the establishment of PRV latency but is critical for viral reactivation in neurons [6]" cites reference 6 (Coen et al., PNAS 1989), which reports exclusively on HSV-1 and contains no data on PRV.

2.Figure numbering error. The in vivo pathogenicity data are cited in the text as Fig. 8 (five instances), but the corresponding legend is labeled "Figure 9," and no Figure 8 legend exists. Please renumber so that text citations and legends correspond. The text refers to 'Fig. 7F, right panel' (Lines 353–354), but the Figure 7 legend and the figure itself contain only panels A–E. Please reconcile panel labeling between text, legend, and figure.

3.Insufficient methodological description of the assays in Figure 7. The figure legend lists "viral gB genome copies (A), IE180 and LAT transcript levels (B), intracellular viral DNA (C), viral protein expression (D), and infectious virus production (E)," yet the Methods do not specify how each measurement was obtained. In particular, the term "extracellular" is used in the Results (Line 339) but not in the legend or Methods. Please specify, for each panel, the sample source (supernatant vs. cell-associated), and ensure consistent terminology throughout.

4.Brain viral loads of PRV-US3-KO relative to PRV-WT (Fig. 8C). In Fig. 8C the brain viral genome copies in PRV-US3-KO-infected mice appear higher than those in PRV-WT-infected mice, which is counterintuitive for a virus lacking an established neurovirulence factor. Whether the difference simply reflects the distinct sampling time points (moribund stage at 7 dpi for PRV-US3-KO versus 6 dpi for PRV-WT), which allowed one additional day of viral replication, and whether neurovirulence in this model is dissociated from the absolute brain viral burden.

5.Sequence validation of recombinant viruses (Fig. S1). The sequence alignment in Fig. S1 is presented without annotation of the four K→R mutation sites, the Us3 insertion junctions, or the identity of the individual sequences, which makes the validation difficult to follow.

Reviewer #3: (No Response)

Reviewer #4: None

--------------------

PLOS authors have the option to publish the peer review history of their article (what does this mean?). If published, this will include your full peer review and any attached files.

If you choose “no”, your identity will remain anonymous but your review may still be made public.

**Do you want your identity to be public for this peer review?**  For information about this choice, including consent withdrawal, please see our Privacy Policy.

Reviewer #1: No

Reviewer #2: No

Reviewer #3: Yes: Subhash Verma

Reviewer #4: No

# Editor report: Acceptance letter

**DOI:** 10.1371/journal.ppat.1014653.r004

**Authors:** Pinghui Feng, Alison McBride

Dear Dr. Zhang,

We are delighted to inform you that your manuscript, "SUMOylation of Pseudorabies Virus Thymidine Kinase Regulates Its Nuclear Localization and Facilitates Neuronal Replication and Reactivation," has been formally accepted for publication in PLOS Pathogens.

We have now passed your article onto the PLOS Production Department who will complete the rest of the pre-publication process. All authors will receive a confirmation email upon publication.

Thank you again for supporting open-access publishing; we are looking forward to publishing your work in PLOS Pathogens.

Best regards,

Dominique Soldati-Favre

Editor-in-Chief

PLOS Pathogens
